# Tung Tree (*Vernicia fordii*) Genome Provides A Resource for Understanding Genome Evolution and Improved Oil Production

**DOI:** 10.1016/j.gpb.2019.03.006

**Published:** 2020-03-26

**Authors:** Lin Zhang, Meilan Liu, Hongxu Long, Wei Dong, Asher Pasha, Eddi Esteban, Wenying Li, Xiaoming Yang, Ze Li, Aixia Song, Duo Ran, Guang Zhao, Yanling Zeng, Hao Chen, Ming Zou, Jingjing Li, Fan Liang, Meili Xie, Jiang Hu, Depeng Wang, Heping Cao, Nicholas J. Provart, Liangsheng Zhang, Xiaofeng Tan

**Affiliations:** ^1^Key Laboratory of Cultivation and Protection for Non-Wood Forest Trees, Ministry of Education, Central South University of Forestry and Technology, Changsha 410004, China; ^2^Key Lab of Non-wood Forest Products of State Forestry Administration, College of Forestry, Central South University of Forestry and Technology, Changsha 410004, China; ^3^Department of Cell and Systems Biology/Centre for the Analysis of Genome Evolution and Function, University of Toronto, Toronto, Ontario M5S 3B2, Canada; ^4^State Key Laboratory of Ecological Pest Control for Fujian and Taiwan Crops, Fujian Provincial Key Laboratory of Haixia Applied Plant Systems Biology, Fujian Agriculture and Forestry University, Fuzhou 350002, China; ^5^College of Forestry, Nanjing Forestry University, Nanjing 210037, China; ^6^Nextomics Biosciences Co., Wuhan 430073, China; ^7^Oil Crop Research Institute, Chinese Academy of Agricultural Sciences, Wuhan 430062, China; ^8^US Department of Agriculture, Agricultural Research Service, Southern Regional Research Center, New Orleans, LA 70124, USA

**Keywords:** Tung tree genome, Tung oil, Genome evolution, Electronic fluorescent pictographic browser, Oil biosynthesis

## Abstract

Tung tree (*Vernicia fordii*) is an economically important woody oil plant that produces **tung oil** rich in eleostearic acid. Here, we report a high-quality chromosome-scale genome sequence of tung tree. The genome sequence was assembled by combining Illumina short reads, Pacific Biosciences single-molecule real-time long reads, and Hi-C sequencing data. The size of **tung tree genome** is 1.12 Gb, with 28,422 predicted genes and over 73% repeat sequences. The *V. fordii* underwent an ancient genome triplication event shared by core eudicots but no further whole-genome duplication in the subsequent ca. 34.55 million years of evolutionary history of the tung tree lineage. Insertion time analysis revealed that repeat-driven genome expansion might have arisen as a result of long-standing long terminal repeat retrotransposon bursts and lack of efficient DNA deletion mechanisms. The genome harbors 88 resistance genes encoding nucleotide-binding sites; 17 of these genes may be involved in early-infection stage of *Fusarium* wilt resistance. Further, 651 oil-related genes were identified, 88 of which are predicted to be directly involved in tung **oil biosynthesis**. Relatively few phosphoenolpyruvate carboxykinase genes, and synergistic effects between transcription factors and oil biosynthesis-related genes might contribute to the high oil content of tung seed. The tung tree genome constitutes a valuable resource for understanding **genome evolution**, as well as for molecular breeding and genetic improvements for oil production.

## Introduction

Tung tree (*Vernicia fordii*), a woody oil plant native to China, is widely distributed in the subtropical area. Tung trees have been planted for tung oil production or ornamental purposes for more than 1000 years in China [1]. They are widely distributed in 16 provinces within China and in many countries, which were also introduced to America, Argentina, Paraguay, and other countries for planting and tung oil production at the beginning of the 20th century [2].

Tung tree taxonomically belongs to the family Euphorbiaceae, along with several other economically important species, including cassava (*Manihot esculenta*), castor bean (*Ricinus communis*), rubber tree (*Hevea brasiliensis*), and physic nut (*Jatropha curcas*). The three major species commonly referred to as tung tree are *V. fordii*, *V. montana*, and *V. cordata*. Of the three, *V. fordii* is the most extensively cultivated species because of its wide geographic distribution, medium stature for easy plantation management, and high-quality oil production.

Tung seed contains 50%−60% tung oil, which is mainly composed (approximately 80% of fatty acid content) of α-eleostearic acid (α-ESA), an unusual fatty acid. α-ESA has three conjugated double bonds (9-*cis*, 11-*trans*, and 13-*trans*), and is hence easily oxidized [3]. Because of its excellent characteristics, tung oil has been widely used as a drying ingredient in paints, varnishes, coating, and finishes since ancient times [2]. Tung oil is also used for the synthesis of thermosetting polymers and resins with superior properties [4], [5], and has been proposed as a potential source of biodiesel [6], [7], [8]. Tung oil had been one of the chief exports to America and Europe until 1980s and experienced a decline in export volume subsequently due to the development of chemical coatings. However, it has been attracting global attention in recent years because of production security, environmental concerns, and negative effect of synthetic chemical coatings on human health [9], [10], [11]. New technologies have been developed to improve the performance of tung oil-based coatings [4], [12], [13].

As an oil crop, economic traits involved in fatty acid biosynthesis and oil accumulation are the targets of improved breeding efficiency of tung tree. However, identification of important genes, gene families, as well as marker loci associated with oil content, fatty acid composition, and fruit yield, has been hampered by a lack of genetic and genomic information. Only a few functional genes, mainly those involved in the formation and regulation of fatty acids, such as the genes encoding fatty acid desaturase (*FAD2*, *FAD3*, and *FADX*) and diacylglycerol acyltransferase (*DGAT*), have been investigated to date [14], [15], [16], [17], [18].

In the present study, we report the sequencing and assembly of *V. fordii* genome, achieved by combining whole-genome shotgun sequencing of Illumina short reads and single-molecule real-time (SMRT) long reads using the Pacific Biosciences (PacBio) platform. We used a Hi-C map to cluster the majority of the assembled contigs onto 11 pseudochromosomes. We also performed evolutionary comparisons and comprehensive transcriptome analysis of genes involved in oil biosynthesis to elucidate the genetic characteristics of oil synthesis and genetic differences with other plant species.

## Results

### Genome sequencing, assembly, and validation

**Figure 1:**
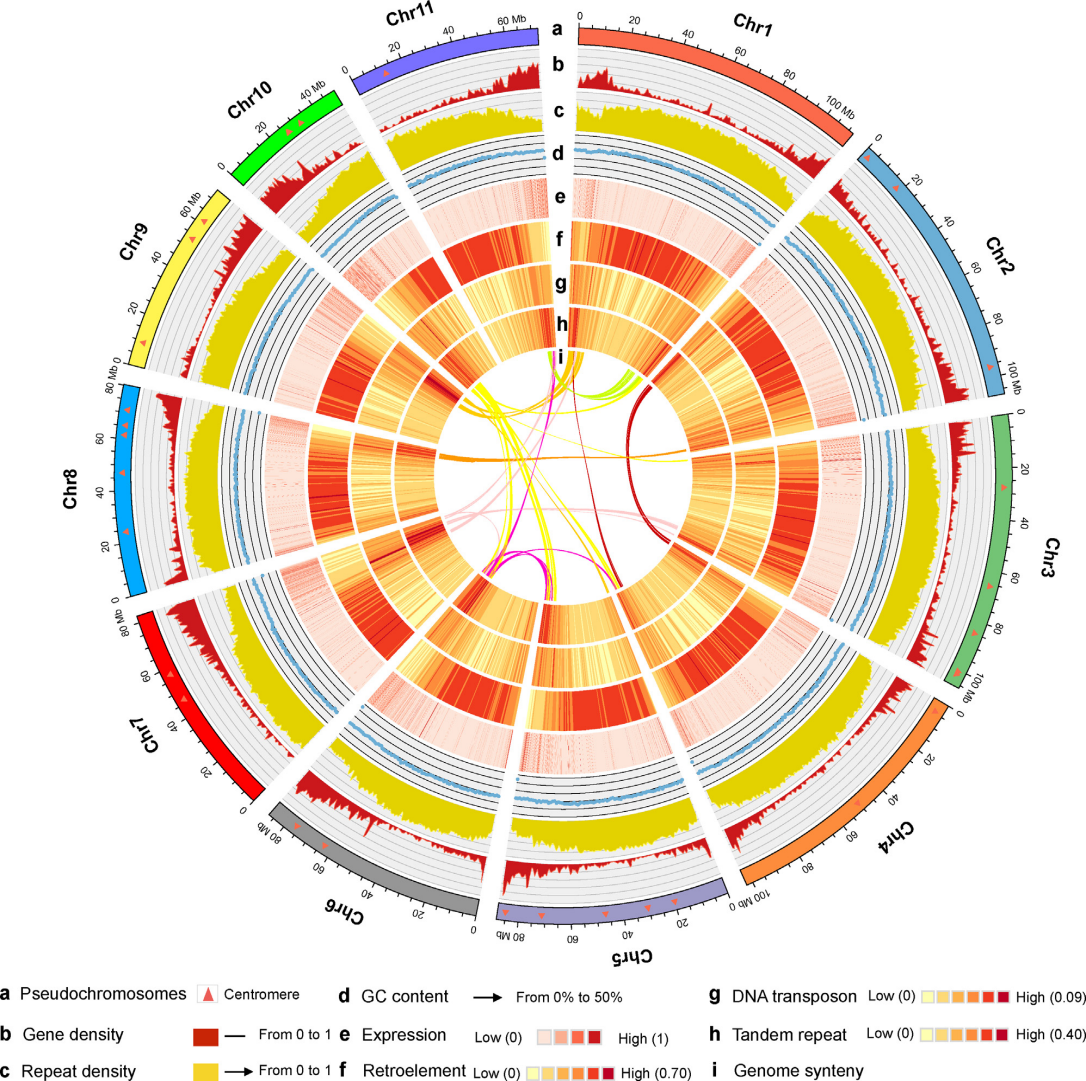
**The genomic landscape of tung tree** The features from outside to inside are pseudochromosomes (**a**), gene density (0–1) (**b**), repeat density (0–1) (**c**), GC content (0%−50%) (**d**), expression (0–1) (**e**), retroelement (0–0.70) (**f**), DNA transposon (0–0.09) (**g**), tandem repeat (0–0.40) (**h**), genome synteny (**i**), Intra-genome collinear blocks with > 20 gene pairs are highlighted with arcs in the middle of the diagram. Different colored line connects matched gene pairs between different chromosomes. Circos was used to construct the diagram. All distributions were drawn using a window size of 1 Mb with the exception of expression, which was drawn using a window of 50 kb. Chr, chromosome.

The self-bred progeny VF1-12 of *V. fordii* cv. Putaotong was used for genome sequencing (File S1). The genome of *V. fordii* was estimated to be 1.31 Gb in size with a low heterozygosity rate of 0.0976% (File S1). After removing low-quality reads, we obtained 177.68 Gb of high-quality data, including 160.21 Gb of Illumina sequencing data and 187.47 Gb of SMRT data, corresponding to 135.73 × coverage of the tung tree genome (Table S1; Figure S2). The assembled tung tree genome, which is 1.12 Gb in size, covering 85% of the estimated genome size, contains 34,773 contigs, with a maximum length of 544.11 kb, and 4577 scaffolds, with a maximum length of 5.09 Mb (Table 1; Table S2). Among them, 3333 contigs and 29,721 scaffolds are over 2 kb long (Table S2). After Hi-C data assessment and assembly, 1.06 Gb (95.15%) of the genome sequences were anchored onto 11 pseudochromosomes, with the scaffold N50 of 87.15 Mb (Table 1; Tables S3 and S4; Figure 1).

**Table 1:** **Statistics of tung tree genome assembly and annotation**.

**Type**	**Parameter**	**Value**
Assembly	Estimated genome size (Gb)	1.31
	Total assembly size (Gb)	1.12
	No. of scaffolds	4577
	Sequences anchored to the Hi-C map (Gb)	1.06
	N50 of scaffolds after Hi-C assembly (Mb)	87.15

Annotation	GC content (%)	31.93
	No. of genes	28,422
	Average gene length (bp)	3785.26
	Average CDS length (bp)	1033.92
	Average No. of exons per gene	4.85
	Average exon length (bp)	213.11
	Average intron length (bp)	714.36
	No. of rRNA genes	116
	No. of tRNA genes	740
	No. of microRNA genes	465
	No. of small nuclear RNAgenes	1414
	Repeat content (%)	73.34
	Total No. of simple sequence repeats identified	66,3931

The Core Eukaryotic Genes Mapping Approach (CEGMA) prediction indicated that 87.9% complete elements and 95.97% partial elements in the tung tree genome could be matched to the 248 most conserved genes (Table S5). The Benchmarking Universal Single-Copy Orthologs (BUSCO) analysis revealed that 1379 (95.7%) of BUSCO genes were complete, of which 1338 (92.9%) and 41 (2.8%) were single-copy and duplicated, respectively (Table S6). RNA sequencing (RNA-seq) analysis revealed that 90.36%, 96.83%, and 93.64% unigenes from male flower, female flower, and seed samples showed good alignment with the assembled tung tree genome, with the mapping rate over 90% (Table S7). Furthermore, 88.3%–95.6% of reads from these five samples were mapped to the genome assembly (Table S8). Validation analysis indicated high quality of the generated tung tree genome assembly.

### Genome annotation

Overall, 28,422 genes were predicted, with an average transcript length of 3785 bp; average CDS length of 1034 bp; average exon number of 4.85 per gene; average exon length of 213 bp; and average intron length of 714 bp (Table 1; Table S9). The GC content was 31.93% across the genome: 41.91% in the coding sequences and 31.16% in intron regions (Table 1; Table S10). BUSCO analysis revealed that 1290 complete BUSCO sequences (89.6%) could be identified from all BUSCO groups, indicating that most of the gene models were complete (Table S11).

Among the predicted 28,422 genes, 23,143 genes (81.4%) were functionally annotated. TrEMBL, SWISS-PROT, and NCBI NR analyses allowed the annotation of 79.6%, 63.8%, and 81.1% of genes, respectively (Table S12). Gene ontology (GO) annotation grouped 12,581 genes into the three categories of molecular function (GO:0003674; 65.97% genes), cellular component (GO:0005575; 20.1% genes), and biological process (GO:0008150; 58.52% genes) (Figure S3). Furthermore, we used Kyoto Encyclopedia of Genes and Genomes (KEGG) to annotate 6835 genes to 235 pathways. Among these pathways, oil biosynthesis and metabolism-related glycerolipid metabolism (ko00561), fatty acid biosynthesis (ko00061), fatty acid elongation (ko00062), and fatty acid degradation (ko00071) were of particular interest in the current study (Table S13).

In addition, we identified several types of non-coding RNAs in the tung tree genome, including 465 microRNA genes, 740 tRNA genes, 116 rRNA genes, and 1414 small nuclear RNA (snRNA) genes (Table S14).

### Gene family evolution and phylogeny

The protein sequences of eight species (*Arabidopsis thaliana*, *Populus trichocarpa*, *Vitis vinifera*, *V. fordii*, *J. curcas*, *R. communis*, *M. esculenta*, and *H. brasiliensis*) were used to identify the gene family by OrthoMCL method [19]. Consequently, 22,991 tung tree genes were clustered into 15,038 gene families, including 635 tung tree-unique families, whereas 5431 tung tree-specific genes were unclustered (Table S15). GO annotation of the tung tree-unique families revealed high enrichment of genes involved in macromolecule metabolic processes (GO:0043170), cellular macromolecule metabolic processes (GO:0044260), and protein metabolic processes (GO:0019538) (Table S16; Figure S4). Furthermore, 933 genes of tung tree-unique families were annotated using KEGG database, of which 586 were mapped to KEGG pathways. The KEGG pathway assignments were enriched in translation (110 genes); carbohydrate metabolism (61); biosynthesis of other secondary metabolites (42); amino acid metabolism (44); folding, sorting, and degradation (44); signal transduction (43); biosynthesis of other secondary metabolites (42); and environmental adaptation (36) (Table S17).

We also identified 11,985 gene families that were shared by the five species (*V. fordii*, *J. curcas*, *R. communis*, *M. esculenta*, and *H. brasiliensis*) of Euphorbiaceae family (Figure S11A). The tung tree shared 13,408, 13,387, 13,519, and 13,216 gene families with *J. curcas*, *H. brasiliensis*, *M. esculenta*, and *R. communis*, respectively, of which 9778 (72.93%), 6643 (49.62%), 7980 (59.03%), and 10,675 (80.77%) gene families exhibited a one-to-one orthologous relationship, accordingly (Figure S5A). In addition, comparison with genomes of *A. thaliana*, *P. trichocarpa*, and *V. vinifera* revealed 3421 gene families specific to Euphorbiaceae (Figure S5B).

**Figure 2:**
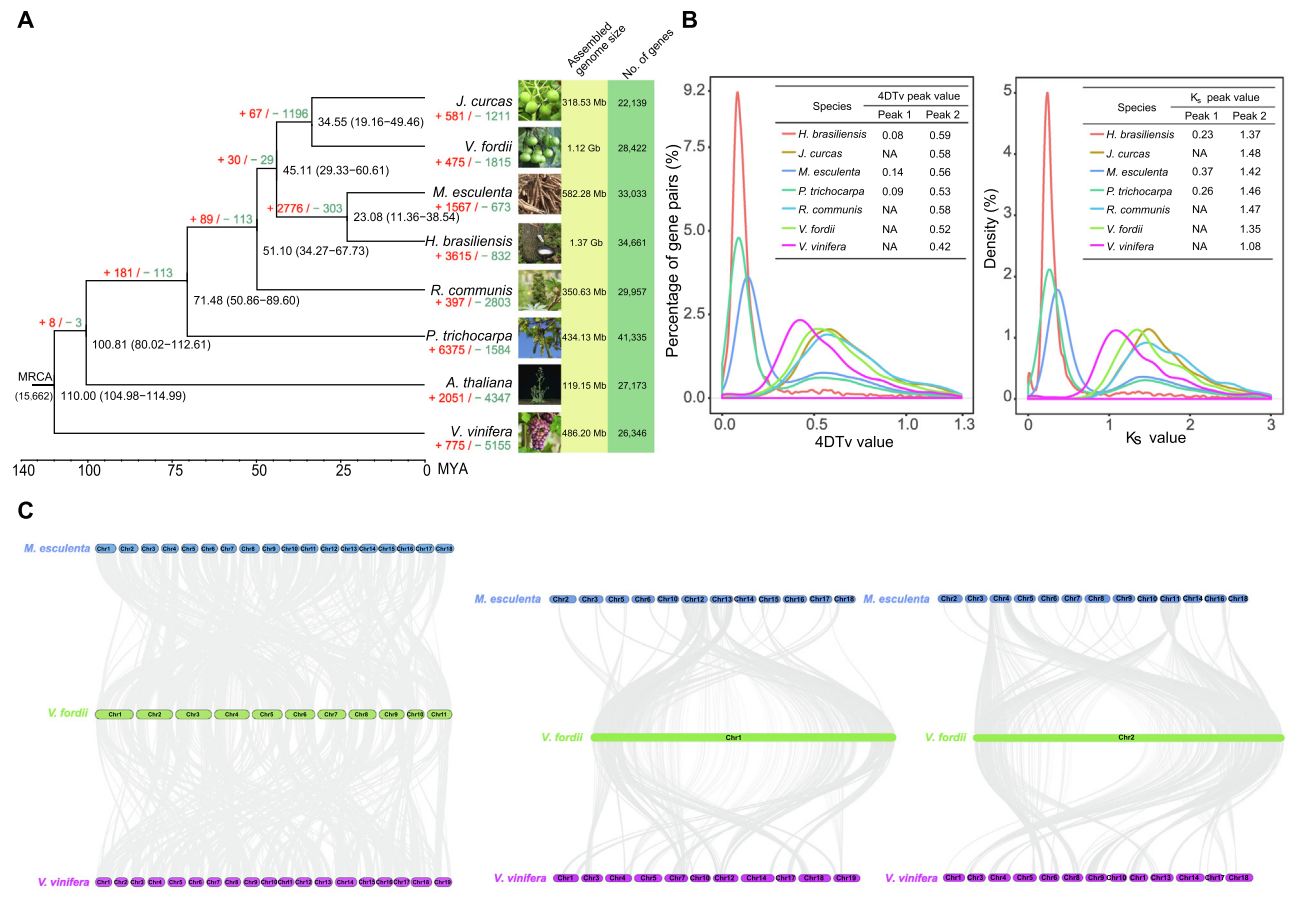
**Evolution of tung tree genome** **A.** Phylogenetic tree of tung tree and 7 other plant species based on orthologues of single-copy gene families. The value at each branch point denotes the estimated divergence time (MYA) with time range provided in the parentheses. The number at the root (15,662) represents the number of gene families in the common ancestor. The values above each branch indicate the numbers of gene family expansion (in red)/contraction (in green) at each round of genome duplication after divergence from the common ancestor. Bootstrap value for each node is 100. **B.** Density distribution of 4DTv and Ks for paralogous genes. The peak value is shown in the inset. “NA” means no peak value. **C.** Collinear relationship of *V. fordii*, *M. esculenta*, and *V. vinifera*. Syntenic blocks determined by using all 11 chromosomes, chromosome 1, and chromosome 2 of *V. fordii* are shown in the left, middle, and right plots, respectively. The gray line connects matched gene pairs. The chromosomes of *V. fordii*, *M. esculenta*, and *V. vinifera* are assigned with green, blue, and purple, respectively. MRCA, most recent common ancestor; MYA, million years ago; 4Dtv, four-fold synonymous third-codon transversion; Ks, the number of synonymous substitutions per synonymous site.

A phylogenetic tree was generated based on 2085 single-gene families in the eight species (Figure 2**A**). We estimated that *V. fordii* and *J. curcas* diverged approximately 34.55 million years ago (MYA) (Figure 2A). The analysis indicated that *V. fordii* is more closely related to *J. curcas* than to *M. esculenta*, *R. communis*, and *H. brasiliensis* in the family Euphorbiaceae, which is consistent with their phylogenetic classification based on morphological characteristics.

The expansion and contraction of gene families in plants occur because plants are subjected to a selection pressure during evolution. These processes thereby play major roles in plant phenotypic diversification [20]. Expansion and contraction analysis of 15,662 gene families as indicated by the phylogenetic tree, produced 475 expanded gene families encompassing 1612 genes and 1815 contracted families in tung tree (Figure 2A). Of the former, 839 gene families were annotated using the GO database. GO annotation revealed highly enriched genes related to macromolecule metabolic processes (GO:0043170), cellular macromolecule metabolic processes (GO:0044260), and nucleotide binding (GO:0000166) (Table S18).

The Ka/Ks ratio (also called ω or dN/dS) represents the ratio of the number of non-synonymous substitutions per non-synonymous site (Ka) to the number of synonymous substitutions per synonymous site (Ks), which is indicative of selective pressure acting on a protein-coding gene. The Ks and Ka values, and the Ka/Ks ratio were determined for each homologous cluster. Consequently, 586 positively selected genes (PSGs) in the tung tree genome were identified, of which 475 were annotated using SWISS-PROT functions (Table S19). GO annotation revealed high enrichment of PSGs related to pigment metabolic processes (GO:0042440), mitochondrial membrane (GO:0031966), and nuclear part (GO:0044428) (Table S20).

### Whole-genome duplication and collinearity

**Figure 3:**
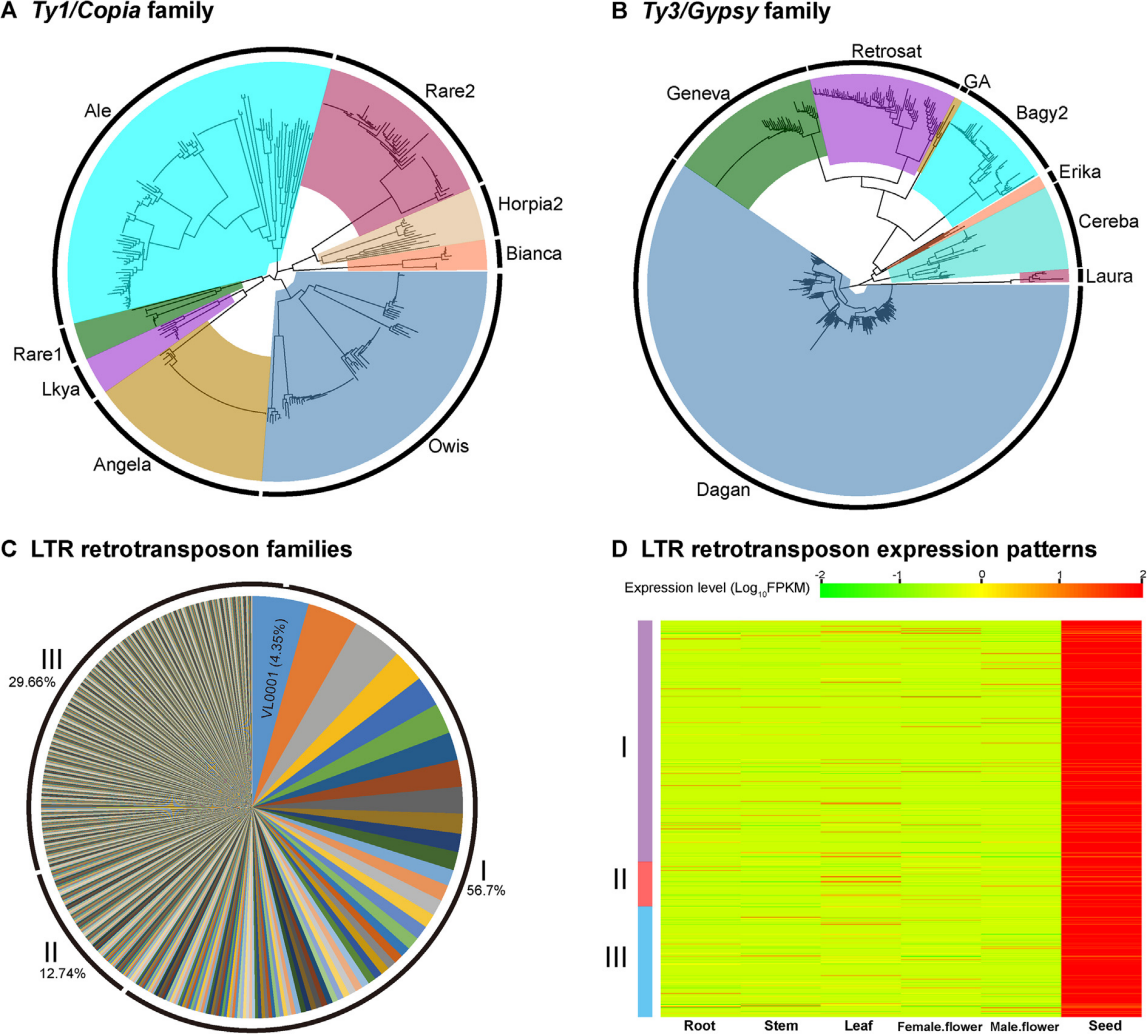
**Analysis of the LTR Retrotransposons in the tung tree genome**. **A.** The neighbor-joining tree based on 347 *Ty1/Copia* sequences. **B.** The neighbor-joining tree based on 622 *Ty3/Gypsy* sequences. **C.** Proportions of LTR retrotransposon families calculated based on their copy numbers in the tung tree genome. **D.** Heat map of expression patterns of 701 LTR retrotransposons (see more details in Table S26). All aligned sequences correspond to the RT domains without premature termination codon. LTR family names and their proportion are indicated. I, II, and III indicate high-copy families (≥ 5 intact members; 89 families), median-copy families (2–4 intact members; 154 families) and single-copy families (887 families), respectively. More details of the data can be found in Table S26 of File S2. LTR, long terminal repeat; RT, reverse transcriptase.

All of the seven species (*P. trichocarpa*, *V. vinifera*, *V. fordii*, *J. curcas*, *R. communis*, *M. esculenta*, and *H. brasiliensis*) showed peak 2, with peak values ranging from 1.08 to 1.48, in four-fold synonymous third-codon transversion (4DTV) analysis, and 0.42 to 0.59, in Ks analysis (Figure 3). However, no peak 1 was observed in *V. fordii*, *J. curcas*, *R. communis*, and *V. vinifera* (Figure 2B). This suggests that only an ancient genome triplication event (*i.e.*, γ event shared by the core eudicots) and no recent independent whole-genome duplication (WGD) events occurred in the subsequent, ca. 34.55-million years of evolutionary history of the tung tree lineage.

Plotting collinear regions identified 122 syntenic blocks containing 2010 collinear gene pairs in the tung tree genome (Figure 1; Table S21). Overall, 3559 genes comprised the collinear gene pairs, accounting for only 12.52% of tung tree genes, a proportion is similar to that in *V. vinifera* (13.91%) but considerably lower than that in *M. esculenta* (33.86%) (Tables S22 and S23). The low collinear rate of tung tree genome suggests that only a small proportion of the genome was duplicated during its evolution, which was consistent with the notion that the genome did not undergo a recent WGD event.

The tung tree genome generally showed one-to-one and one-to-two syntenic relationships with *V. vinifera* (one duplication) and *M. esculenta* (two duplications), respectively (Figure 2C). The tung tree genome shared 694 syntenic blocks containing 22,133 collinear gene pairs with *M. esculenta*, and 589 syntenic blocks containing 14,570 collinear gene pairs with *V. vinifera* (Figure 2C). For most collinear regions between tung tree and *M. esculenta*, one chromosome of tung tree corresponded to two chromosomes of *M. esculenta* (Figure 2C). For instance, VfChr1 of tung tree corresponded to MeChr12 and MeChr13 of cassava. Similarly, VfChr2 corresponded to MeChr4 and MeChr11; VfChr3 corresponded to MeChr7 and MeChr10; VfChr5 corresponded to MeChr1 and MeChr2; and VfChr6 corresponded to MeChr1 and MeChr5. These observations indicate that VfChr1, VfChr2, VfChr3, and VfChr5 of tung tree may have formed by fragmentation and recombination of ancestral chromosomes. The collinear regions between tung tree and *V. vinifera* did not exhibit any marked corresponding chromosome relationships, in contrast to those between tung tree and *M. esculenta*.

### Repeat-driven genome expansion

The tung tree genome was larger than that of physic nut and castor bean, which was mainly attributed to repeat expansion. Repetitive element analysis revealed that the tung tree genome had the highest repeat content (73.34%) among the five sequenced Euphorbiaceae species genomes (Table S24), which was slightly higher than that of the rubber tree (71%) [21], and much higher than that of the castor bean (50.33%) [22], physic nut (49.8%) [23], and cassava (less than 40%) [24]. The repeat sequences were distributed at both ends of each tung tree chromosome (Figure 1). We identified 663,931 simple sequence repeats (SSRs) in the tung tree genome. The annotated SSRs were mostly mononucleotide repeats (39.62%) and dinucleotide repeats (13.38%) (File S2). Retroelements accounted for the majority (51.89%) of the tung tree genome; 50.77% retroelements were long terminal repeat (LTR) retrotransposons (Table S25). Two types of LTR retrotransposons, *Ty1/Copia* (84,180 elements) and *Ty3/Gypsy* (284,597 elements), were most abundant, accounting for 15.13% and 53.46% of the total repeat sequences, respectively (Figure 3A and B; File S2; Table S25).

Kimura analysis indicated that two LTR retrotransposon types (*Ty1/Copia* and *Ty3/Gypsy*) and DNA transposons were almost simultaneously amplified, with similar peaks for amplification bursts (Figure S6). Insertion time analysis of intact LTR retrotransposons indicated that both *Ty1/Copia* and *Ty3/Gypsy* underwent multiple bursts over the last 3–4 MYA, and that they were younger than other unclassified transposable elements (File S2; Figures S7 and S8). In addition, median-copy families and high-copy families were younger than single-copy families (Figure S9). In light of this analysis, the marked expansion in tung tree genome size might be associated with long-standing LTR retrotransposon bursts and a lack of efficient DNA deletion mechanisms. VL0001 was the largest *Ty3/Gypsy* family, with 130 copies, accounting for 7.54% of the high-copy families and 4.35% of LTR retrotransposons (Figure 3C; File S2).

Based on the RNA-seq data, 1738 out of 2991 LTR retrotransposons were expressed in six tissues. *Ty3/Gypsy* LTR retrotransposons generally exhibited higher expression levels than *Ty1/Copia* retrotransposons, ranging from 0.71-fold in the seed to 4.09-fold in the leaf, with approximately twofold higher on average (File S2). Among 1738 LTR retrotransposons, 701 showed the highest expression level in the seed, of which 60.77% belonged to high-copy families (Figure 3D; File S2). This suggests that abundant high-copy LTR retrotransposons might be more active than other LTR retrotransposon families in the developing tung seed. In addition, 184, 204, 244, 148, and 257 LTR retrotransposons exhibited the highest expression levels in the root, stem, leaf, female flower, and male flower, respectively (File S2). Among these LTRs, high-copy LTR families also accounted for the highest proportion in the five tissues.

### The tung tree electronic fluorescent pictographic browser

**Figure 4:**
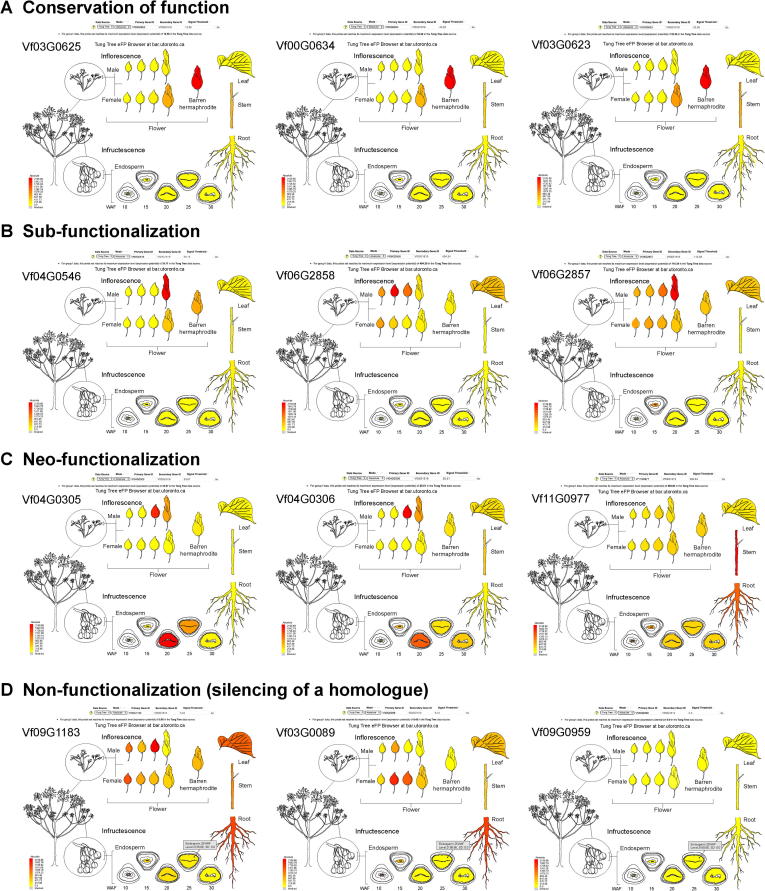
**Functional conservation and diversification of tung tree homologs as visualized with the tung tree eFP browser** eFP browser images showing conservation of function (**A**), sub-functionalization (**B**), neo-functionalization (**C**), and non-functionalization (**D**) of tung tree homologs. In each panel, the expression patterns of three homologs of each gene are shown. In all cases, red represents higher levels of transcript accumulation and yellow represents a lower level of transcript accumulation. From A to D, genes encoding F6′H (from left to right Vf03G0652, Vf00G0634, and Vf03G0623), protein ECERIFERUM (from left to right Vf04G0546, Vf06G2858, and Vf06G2857), PAP (from left to right Vf04G0305, Vf04G0306, and Vf11G0977), and protein LYK5 (from left to right Vf09G1183, Vf03G0089, and Vf09G0959), are shown, respectively. WAF, week after flowering; eFP, electronic fluorescent pictographic; F6′H, feruloyl CoA ortho-hydroxylase; PAP purple acid phosphatase; LYK, lysin motif receptor kinase.

The genome-wide gene identification allowed us to investigate gene expression on a large scale in tung tree. To allow easy access to and enable visualization of the expression levels of tung tree genes, the flowers and seeds at different developmental stages were sampled for RNA-seq analysis (File S3). Based on RNA-seq data from 17 samples, a tung tree electronic fluorescent pictographic (eFP) browser (http://bar.utoronto.ca/efp_tung_tree/cgi-bin/efpWeb.cgi) was devised to permit the visualization of gene expression patterns in “absolute”, “relative”, and “compare” modes in these tissues using the annotated gene IDs (File S3). The search interface generated an eFP colored according to transcript abundance data for the individual tung tree gene in various tissues or organs. As exemplified in Figure S11, the expression pattern of *VfFADX-1* (Vf11G0298), encoding an enzyme that uses linoleic acid (C18:2Δ9,12) as a substrate to produce α-ESA (18:3Δ9,11,13), was consistent with its role in oil biosynthesis. In addition, the tung tree eFP browser could be used for the functional characterization of tung tree gene copies with different expression patterns. For instance, the function of three feruloyl CoA ortho-hydroxylase (F6′H) homologues (Vf03G0652, Vf00G0634, and Vf03G0623) was conserved, as revealed by similar expression patterns of the encoding genes in various tissues/organs (Figure 4; Table S35). Furthermore, among the three purple acid phosphatase (PAP) homologues, Vf11G0977 protein displayed neo-functionalization, *i.e*., functional diversification, and was expressed in the root, unlike the other homologues (Figure 4; Table S35).

### Nucleotide-binding site-coding resistance genes

Disease resistance is one of the most important traits in tung tree breeding programs. *V. fordii* is susceptible to wilt (*Fusarium oxysporum*), black spot (*Mycosphaerella aleuritidis*), and twig dieback (*Nectria aleuritidia*). Information on disease resistance-related genes sheds light on plant resistance mechanisms. Furthermore, identification and characterization of these genes on a genome-wide scale provides a basis for the improvement of disease resistance in tung tree.

**Figure 5:**
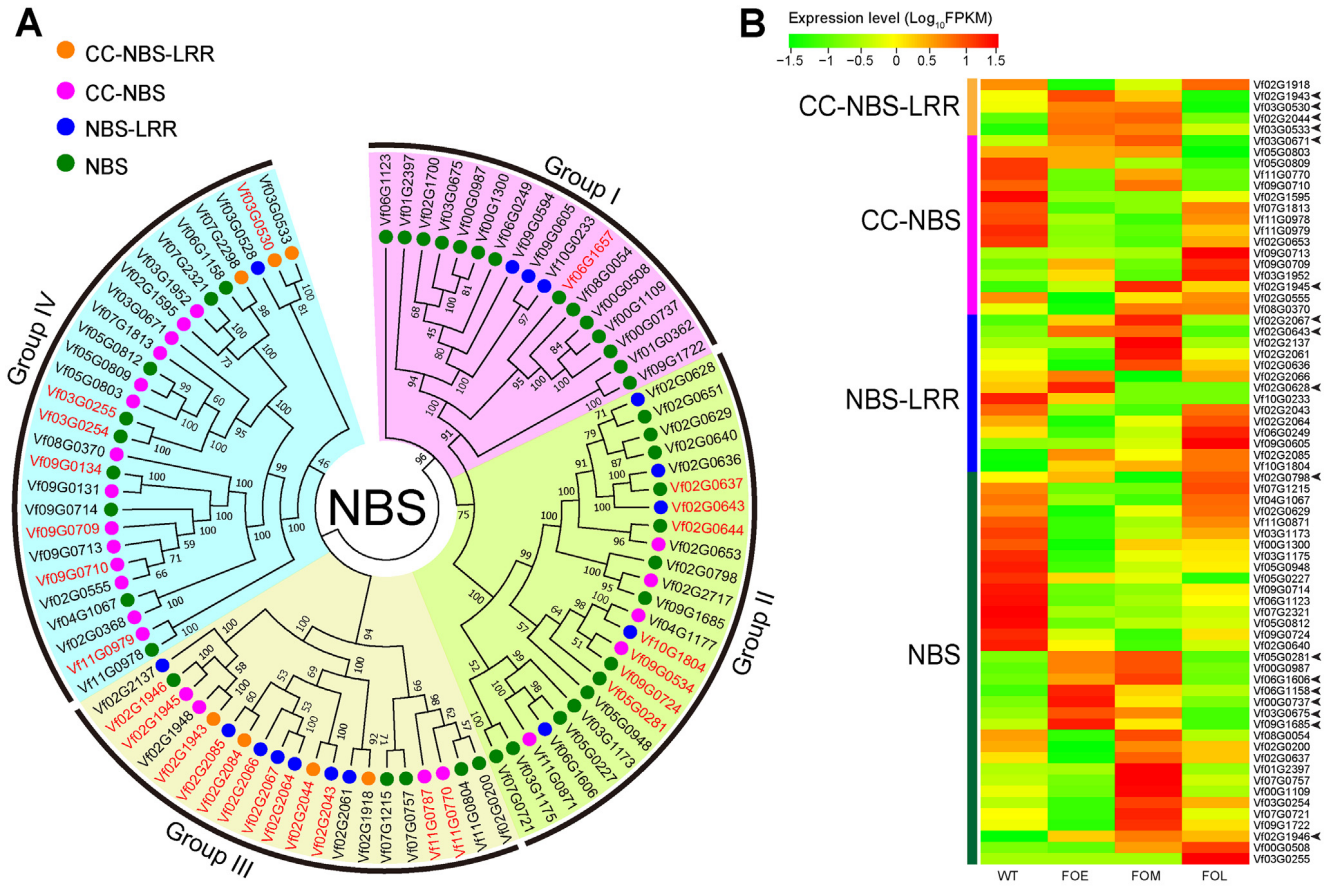
**The NBS-coding genes in tung tree genome** **A.** The maximum-likelihood phylogenetic tree based on 88 tung tree NBS-coding genes. The 88 genes are classified into four groups (group I–IV) according to four clades generated in the tree. Groups I–IV are marked by four different block colors. Dots in green, blue, pink, and orange indicate NBS, NBS-LRR, CC-NBS, and CC-NBS-LRR subfamilies, respectively. Gene IDs in red indicate tandem repeats. **B.** Heat map of expression patterns of tung tree NBS-coding genes. The arrows indicate NBS genes responding to *Fusarium* wilt. FOE, FOM, and FOL represents early, middle, and late stage after *F. oxysporum* infection. NBS, nucleotide-binding site; LRR, leucine-rich repeat; CC, coiled-coil; WT, wild type.

Genes encoding nucleotide-binding sites (NBSs) are the largest class of plant disease-resistance genes. Based on whether they contain a Toll/interleukin-1 receptor (TIR) domain, NBS resistance genes can be further categorized into two subclasses, TIR and non-TIR (File S4). Overall, 88 genes with an NBS domain were identified in the tung tree genome, of which 28 (31.82%) were organized in tandem arrays (Table S36; Figure 5A). The number of NBS-coding genes in *V. fordii* was similar to that in *Z. mays* (107), but markedly lower than that in *R. communis* (232), *M. esculenta* (312), *J. curcas* (275), and *H. brasiliensis* (483) (Table S36). The NBS-coding genes were classified into four subfamilies, namely, 23 coiled-coil (CC)-NBS genes, 16 NBS-leucine-rich repeat (LRR) genes, 7 CC-NBS-LRR genes, and 42 NBS genes; however, they did not form four independent classes in the phylogenetic tree (Figure 5A). Intriguingly, none of the tung tree NBS-coding resistance genes belong to the TIR subclass (Table S36).

The NBS genes were distributed nonrandomly across all (11) chromosomes (Figure S12). More than 85% NBS genes were clustered in groups; the clusters were most abundant on chromosomes 2, 9, and 3 (Figure S12). Enrichment of NBS genes in the corresponding genomic regions indicates that the evolution of resistance genes might have involved tandem duplication and divergence of linked gene families, as described in other plant genomes, such as those of the rubber tree [25] and pear [26]. RNA-seq analysis revealed differential expression patterns of all tung tree NBS genes in the root after *F. oxysporum* infection (Figure 5B). The expression level of 17 genes (eight NBS, three NBS-LRR, two CC-NBS, and four CC-NBS-LRR genes) increased at an early stage after *F. oxysporum* infection (FOE) and decreased at a late infection stage after *F. oxysporum* infection (FOL) (Figure 5B). These observations suggest that these genes may be involved in a protective mechanism against the pathogen shortly after an infection.

### Evolution of genes involved in oil biosynthesis

**Figure 6:**
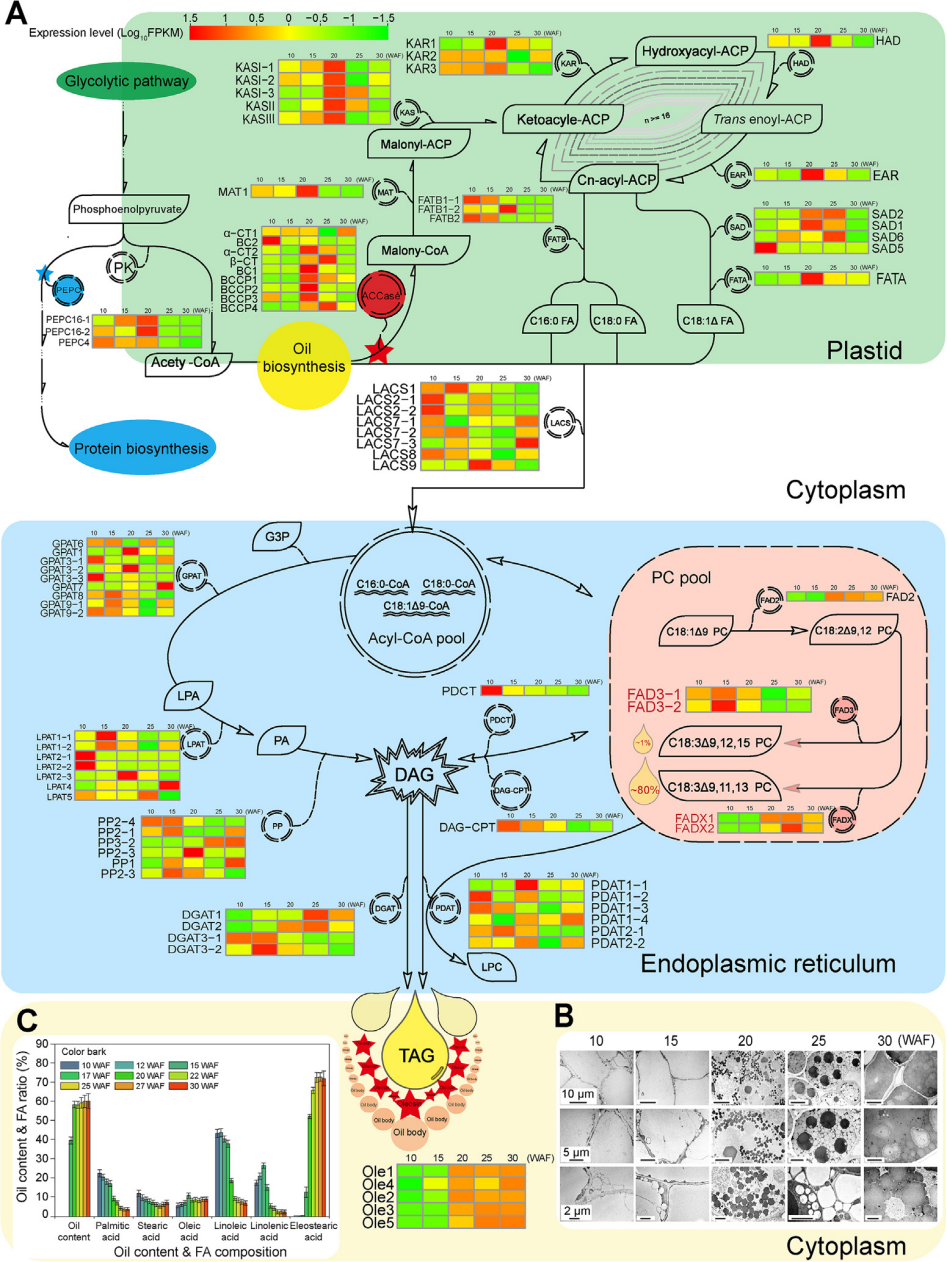
**Network of genes involved in tung oil biosynthesis** **A.** Tung oil biosynthesis pathway. Tung oil biosynthesis is catalyzed by 18 enzymatic steps with multiple isozymes in each step. Acetyl-CoA is converted into C16 and C18 fatty acids in the plastid. TAG is synthesized in the endoplasmic reticulum and packed in the oil bodies. Metabolites are described in the black box, and enzymes are circled between two metabolite boxes. The expression levels of oil-biosynthesis genes are presented with the heat map. The scale bar of relative expression levels are shown at the top left. **B.** Oil droplet development in tung tree seeds after flowering. Images in the top and middle rows showing the oil droplet development were taken in multicellular visual field and single cell visual field, respectively. Images in the bottom row show the alterations in oil droplet shape over time. **C.** Tung oil and FA accumulation profiles. Tung oil was extracted from the endosperms and converted to methyl esters by potassium hydroxide-methanol solution. Fatty acids were separated and quantified by GC. Oil content = (oil quantity/dry endosperm quantity) × 100%. FA ratio = (individual fatty acid peak under the curve/total fatty acid peaks under the curve) × 100%. PEPC, phosphoenolpyruvate carboxylase; PK, pyruvate kinase; ACCase, acetyl CoA carboxylase; α/β-CT, acetyl-coenzyme A carboxylase carboxyl transferase subunit alpha/ beta; BCCP, biotin carboxyl carrier protein; BC, biotin carboxylase; MAT, malonyl-CoA transacylases; KAS, ketoacyl-ACP synthase; KAR, ketoacyl-ACP reductase; HAD, hydroxyacyl-ACP dehydrase; EAR, enoyl-ACP reductase; FAT, fatty-ACP thioesterase; SAD, stearoyl-ACP desaturase; FA, fatty acid; LACS, long-chain acyl-CoA synthetase; G3P, glycerol-3-phosphate; GPAT, glycerol-3-phosphate acyltransferase; LPA, lysophosphatidic acid; LPAT, lysophosphatidic acid acyltransferase; PA, phosphatidic acid; PP, phosphatidate phosphatase DAG, diacylglycerol; PDCT, phosphatidylcholine; DAG-CPT, CDP-choline-diacylglycerol cholinephosphotransferase; PC, phosphatidylcholine; FAD, fatty-acid desaturase; DAGT, diacylglycerol *O*-acyltransferase; PDAT, phospholipid-DAG acyltransferase; LPC, lyso-phosphatidylcholine. TAG, triacylglycerol; Ole, oleosin; ACP, acyl carrier protein.

Tung oil is the most important product derived from tung tree. Tung oil biosynthesis starts from acetyl-CoA and involves 18 enzymatic steps catalyzed by multiple isozymes (Figure 6A). The oil is packed in subcellular structures called oil bodies or lipid droplets (Figure 6B; File S5). We observed that tung seed oil droplets formed following the pattern of α-ESA accumulation in the seed (Figure 6B and C). No visible oil droplet was observed in the seed at 10 weeks after flowering (WAF) and small oil droplets were observed at 15 WAF. The number and size of oil droplets were markedly increased in mature seeds (20, 25, and 30 WAF seeds).

Tung oil biosynthesis in the seed started in mid-June (10 WAF), increased rapidly up to 25 WAF, with the oil content of 59.22% (Figure 6C), and ended by 30 WAF. Oleic acid (C18:1Δ9) was a minor oil component, whereas linoleic acid (C18:2Δ9,12) was the major oil component (43%) in young seed (10 and 15 WAF). The levels of both fatty acids gradually decreased in mature seed. Accumulation of linoleic acid and α-ESA (α-C18:3Δ9,11,13) exhibited opposite patterns in the developing tung seed (Figure 6C) because linoleic acid acts as a substrate for the synthesis of α-ESA and α-linolenic acid (α-ALA; C18:3Δ9,12,15). α-ESA synthesis started after 15 WAF and then increased rapidly to reach up to 72.35% of seed oil following seed ripening (Figure 6C). α-ALA accumulation was observed in 10 WAF seeds; the compound was a minor oil component during the entire developmental process. We used these developmental patterns of α-ESA biosynthesis and oil droplet formation as the criteria for selecting seed stages for the ensuing transcriptomic analysis.

Among 23,143 genes annotated in the tung tree genome, 651 genes were related to oil biosynthesis (Table S37). Among them, 88 genes were predicted to be directly involved in oil biosynthesis (Figure 6A; File S6; Table S38), a number considerably higher than the number of sequences for tung oil-related genes deposited in the GenBank databases (29 genes). The identified genes belong to 18 families; their expression profiles are shown in Figure 6A. The number of tung oil-related genes was within the range of that in other plant species, *i.e.*, 91 genes in *J. curcas*, 84 genes in *R. communis*, 87 genes in *A. thaliana*, 105 genes in *S. indicum*, and 210 genes in *G.* max (Table S38).

Several key genes for oil biosynthesis have been extensively studied, including acetyl-CoA carboxylase (ACCase), FAD, DGAT, and oleosin (OLE) genes (Figure 6A). In the current study, we identified one additional *DGAT3* and two additional FAD genes in addition to those reported previously. We also reported for the first time six phospholipid: diacylglycerol acyltransferase (PDAT) genes in the tung tree genome (Figure 6A).

ACCase and phosphoenolpyruvate carboxykinase (PEPC) are most likely the key enzymes determining the metabolic pathways that lead to oil or protein biosynthesis in the seed (Figure 6A) [27]. We identified nine ACCase genes in the tung tree genome that had high expression levels in the mid-late developmental stages of tung seed (Figure 6A). In comparison, the soybean genome harbors 10 ACCase genes, and other species harbor 6–7 such genes (Table S38). We also identified three PEPC genes in the tung tree genome, which were expressed in the early developmental stages of tung seed (Figure 6A; Table S38). In addition, the soybean genome harbors 16 PEPC genes and other species harbor more PEPC genes than tung tree. Compared with soybean, whose seed has a high protein content (approximately 40%) and low oil content (approximately 20%), the relatively fewer PEPC genes in the tung tree genome might explain the high oil (approximately 60%) and low protein content (approximately 5%) in tung seed, probably contributing to carbon flow toward fatty acid biosynthesis therein.

**Figure 7:**
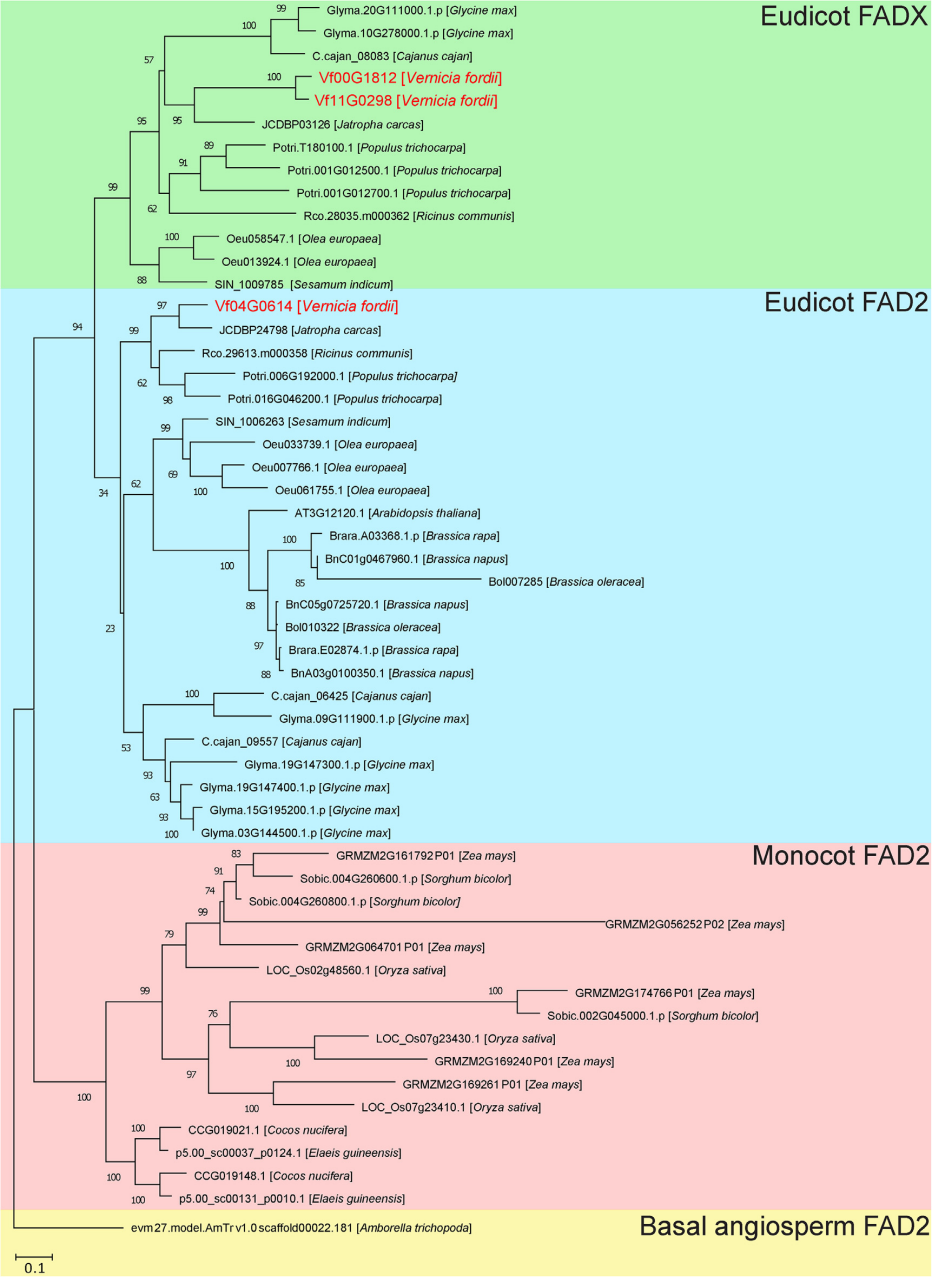
**Phylogeny of FAD2 and FADX proteins** A maximum-likelihood phylogenetic tree was constructed from FAD protein sequences. The taxon names in the phylogenetic tree are indicated after gene ID. The clades are marked by four different block colors in the tree. For the last one (yellow), a basal angiosperm, *Amborella trichopoda*, was used as an outgroup. The monocot FAD2, eudicot FAD2, and eudicot FADX clades are marked in red, blue, and green, respectively.

The FAD protein family catalyzes the desaturation of fatty acids [6] and is therefore responsible for polyunsaturated lipid synthesis in the developing seed of oil crops. FAD2 and FAD3 are the main enzymes responsible for the Δ12 linoleic acid and Δ15 linolenic acid desaturation, respectively. We identified one *FAD2*, two *FAD3*, and two *FADX* genes in the tung tree genome (Table S38). *FAD2* and *FADX-1* were highly expressed in the mid-late stages of seed development, whereas *FAD3* was highly expressed in the early stages of seed development (Figure 6A). FADX, a divergent FAD2, converts linoleic acid to α-ESA [15], but the evolutionary relationship between FADX and FAD2 remains unclear. According to the phylogenetic tree generated in the current study (Figure 7), the FAD2/X clade is divided into two clades (FAD2 and FADX) in eudicot plants, suggesting that the two clades have arisen by gene duplication in eudicot ancestors. The eudicot ancestors experienced a γ WGD event, and gene duplication is likely to be retained by a WGD event. Further synteny analysis revealed that *FAD2* and FAD*X* genes were likely generated by a WGD event (Table S39), which corresponded to the γ WGD event shared by core eudicots. Notably, many genes from the FADX clade were lost in such species as the members of Brassicaceae.

DGAT protein family catalyzes the last step of triacylglycerol (TAG) biosynthesis and is regarded as the rate-limiting step of TAG accumulation. Three DGAT genes were reported in tung tree in previous studies [14], [18]. *DGAT2* was proposed to be the most important *DGAT* gene for TAG biosynthesis in tung tree seed. The transcriptomics analysis preformed in the current study revealed the expression of four DGAT genes (*DGAT1*, *DGAT2*, and two *DGAT3* genes) in tung seed (Figure 6A; Table S40). We confirmed that *DGAT2* was the most highly expressed DGAT gene in tung seed, which corresponded to oil accumulation (20–30 WAF), but *DGAT3-1* was the dominant form of DGAT gene in immature seed (10–15 WAF) and other tissues, including the stem, root, leaf, and female flower (Figure 6A; Table S40).

Recently, it has become apparent that TAG synthesis is also catalyzed by PDAT [28]. We reported here for the first time that the tung tree genome encodes five PDAT genes. *PDAT1-1* and *PDAT1-4* were mainly expressed in the mid-late stages of seed development, while the other three PDAT genes were mainly expressed in the early stages of seed development (Figure 6A).

OLEs are the major proteins in plant oil bodies. Genome-wide phylogenetic analysis and a multiple sequence alignment demonstrated that the five tung OLE genes represented five OLE subfamilies. All tung OLEs contain the proline knot motif (PX5SPX3P) shared by 65 OLEs from 19 tree species [29]. We confirmed the presence of five tung tree OLE genes coding for small hydrophobic proteins. These five OLE genes were highly expressed in the mid-late stage of tung seed development (Figure 6A; Table S40).

Furthermore, we identified eight long chain fatty aycl-CoA synthetase (LACS) genes in the tung tree genome, of which *LACS1* and *LACS2* were most highly expressed at an early stage of seed development, while *LACS7*, *LACS8*, and *LACS9* were highly expressed in mid-late stages of seed development (Figure 6A). In addition, nine glycerol-3-phosphateacyltransferase (GPAT), seven lysophosphatidic acid acyltransferase (LPAT), and six phosphatidate phosphatase (PP) genes were identified in the tung tree genome. The expression levels of some of these genes were higher in the early stage of seed development than at late stages of seed development, and *vice versa* (Figure 6A; Table S40).

**Figure 8:**
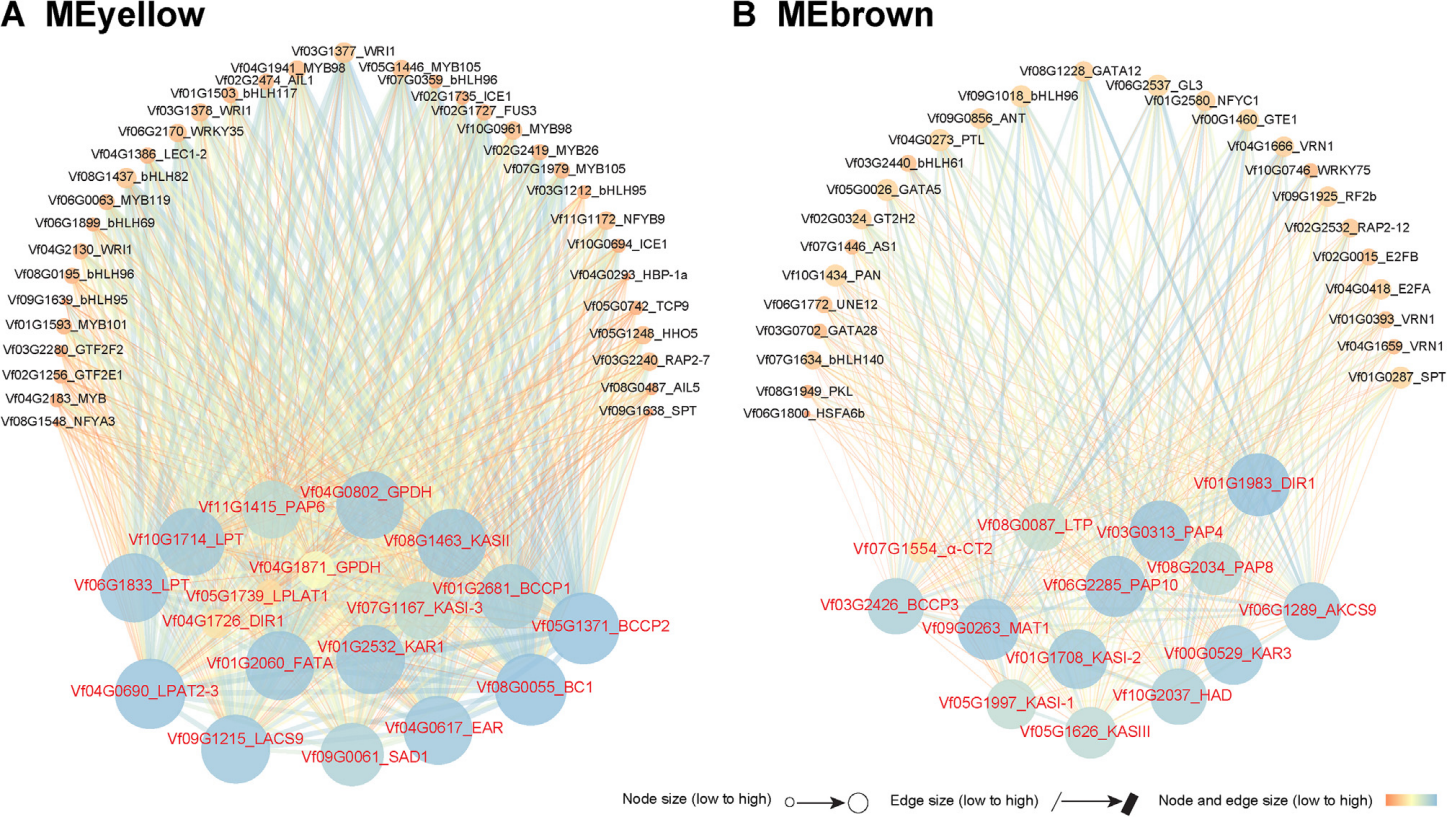
**Co-expression networks of tung tree oil biosynthesis-related genes and transcription factors at the transcriptome level** The two modules showed significant co-expression events in the seed at 20 WAF when oil biosynthesis-related genes were highly enriched. In total, 18 and 13 oil biosynthesis-related genes were identified in MEyellow **A.** and MEbrown **B.**, respectively. These 31 genes are colored in red, and their adjacent genes encoding transcription factors are colored in black. Node size of a gene represents the number of the adjacent genes connecting this gene. Edge size indicates the weight value of two genes (Tables S41 and 42). MEyellow, yellow module; MEbrown, brown module.

To explore the possible synergistic effects among the genes involved in oil accumulation, we performed a weighted correlation network analysis of transcript expression in the seed at five developmental stages (FPKM ≥ 1) (File S7). We identified 10 co-expression modules for each seed sample. The oil accumulation was rapidly increased in the tung seed at 20 WAF. Interestingly, two modules, brown module (MEbrown, containing 1156 genes) and yellow module (MEyellow, containing 908 genes) showed significant co-expression events at 20 WAF when oil biosynthesis-related genes were highly enriched with Pearson correlation coefficient (PCC) ≥ 0.8 and *P* ≤ 0.1 (Tables S41 and 42; Figures S18 and S19). In the MEyellow and MEbrown modules, 18 and 13 genes, respectively, were identified as playing pivotal roles in fatty acid synthesis and oil accumulation, *e.g*., genes for fatty acid synthases (FASs), the upstream rate-limiting enzyme ACCase subunits (α-CT, BCCP-1, BCCP-2, BCCP-2, and BC-1), and genes related to TAG assembly (*e.g*., GPDH and LPAT) (Figure 8). A number of transcription factors were also identified in the two modules and co-expression networks (Figure 8), including WRINKLED1 (*WRI1*), FUSCA3 (*FUS3*), LEAFY COTYLEDON1 (*LEC1*), and ABSCISIC ACID INSENSITIVE3 (*ABI3*), which reportedly facilitate oil accumulation by interacting with each other or with oil biosynthesis-related genes [30], [31], [32], [33], [34]. We selected four genes encoding tung tree transcription factors (*FUS3*, *ABI3*, *LEC1-1*, and *LEC1-2*) for a yeast two-hybrid assay (File S8), and observed an interaction between FUS3 and LEC1-2 (Figure S20). Gene co-expression network analysis indicated that transcription factors and oil biosynthesis-related genes have synergistic effects in oil biosynthesis, which may contribute to the high oil content in tung seed.

## Discussion

Whole genomes of an increasing number of plant species are being sequenced because of a rapid development of new sequencing technologies in recent years. The genome information provides a useful resource for enabling better understanding of a plant’s evolutionary history and for exploring important genes to uncover the mechanisms controlling various traits during long-term evolution. As an economically important tree species, tung tree has been cultivated and utilized for thousands of years. Tung oil has a great potential for the production of environmentally friendly coatings with low volatile organic compounds (VOCs). However, production of tung oil on an industrial scale is hampered by low yield. The genome sequencing efforts summarized in the current study would facilitate the breeding of elite cultivars with yield-related traits, including fruit setting rate and seed oil content.

The large amount of repeat sequences and low GC content in the tung tree genome made sequencing of the genome using whole-genome sequencing strategies involving next-generation sequencing technology challenging, even though the heterozygosity of the tung tree genome is markedly low. To overcome the challenge of high repeat content, we generated long reads from 10-kb and 20-kb libraries via PacBio sequencing. We then used the Hi-C map to generate a chromosome-scale assembly of the tung tree genome. Among the analyzed members of the family Euphorbiaceae in this study, the genomes of rubber tree and cassava, but not those of tung tree, physic nut, and castor bean, were found to undergo a recent WGD event, although these plants all share an ancient WGD event. Interestingly, the rubber tree and cassava genomes harbor more genes than the other three species (Figure 2A). The recent WGD event has caused chromosomal rearrangements, fissions, or fusions, and is one of the reasons for gene family expansion [20]. It may have contributed more genes in rubber tree and cassava than in tung tree, physic nut, and castor bean.

The size of tung tree genome exceeds that of physic nut and castor bean genomes. In most cases, genome expansions are caused by repeated sequence insertion, *e.g*., as observed in tea tree [35], rubber tree [21], and ginkgo (*Ginkgo biloba*) [36]. Similar to these three species, *Ty3/Gypsy* families contributed the most to the tung tree genome expansion. Based on the insertion time analysis performed in the current study, we propose that a lack of efficient mechanisms deleting repeated DNA sequences might have resulted in long-term and continuous LTR retrotransposon bursts and growth, eventually leading to the whole-genome size expansion in tung tree. This is also consistent with the findings for tea tree and Norway spruce [37]. Moreover, we found that different LTR retrotransposon families were differentially expressed in various tissues, confirming the retrotransposon activity in the tung tree genome.

The eFP browser is a useful tool for visualizing gene expression in several plant species, including *A. thaliana*, *P. trichocarpa*, *G.* max, *Solanum tuberosum*, *Solanum lycopersicum*, *Camelina sativa*, *Fragaria vesca*, and others [38], [39], [40], [41]. Based on the tung tree genome sequences generated in the current study, we created the tung tree eFP browser to display tung tree RNA-seq data from 17 different tissues and stages. The use of this eFP browser should facilitate further studies on tung tree and other Euphorbiaceae plants.

Plant disease resistance has always been a research hotspot. NBS genes are the largest class of plant disease resistance genes. They confer the plant the ability to resist the invasion of exogenous pathogens, including bacteria, fungi, and viruses [42]. Genes encoding the TIR domain-containing NBSs are widely distributed in dicots but not monocots, whereas they have been lost from the tung tree genome. To date, loss of genes encoding the TIR domain-containing NBSs from a dicot genome has only been reported for tung tree and sesame [24]. This finding provides a new paradigm to investigate the evolution of disease resistance genes in plants. CC is the functional domain of many proteins and the CC structure plays an important role in protein–protein interaction [43]. LRR constitutes the signal region in the transmembrane domain and its loss can result in loss of function of the harboring proteins [44]. In the current study, the highest proportion of CC-NBS-LRR genes (4/7, 57.14%) responded to *F. oxysporum* infection at an early infection stage, suggesting that CC and LRR domains may play more important roles than other domains in pathogen resistance.

Tung tree is a highly efficient photosynthetic tree with a high photosynthesis rate. Sucrose, the major photosynthesis product, is synthesized in the chloroplast and exported to the sink tissues, such as the seed, for seed development and metabolite accumulation. Sucrose is converted into hexose phosphate, triose phosphate, PEP, and pyruvate. PEP is a key intermediate metabolite for the synthesis of both fatty acids and proteins. It is converted into pyruvate by a pyruvate kinase (PK). Pyruvate is subsequently converted into acyl-CoA and enters the fatty acid biosynthesis pathway via a reaction catalyzed by ACCase. In contrast, PEP is catalyzed by PEPC to produce oxaloacetic acid, which is subsequently used for protein synthesis. Therefore, ACCase and PEPC are probably the key enzymes determining the metabolic pathways toward oil or protein biosynthesis in the seed [27]. We identified nine ACCase genes in the tung tree genome that were highly expressed in the mid-late developmental seed stages. This is indicative of their importance in tung oil biosynthesis. The soybean genome harbors 10 ACCase genes and other species harbor 6–7 such genes (Table S38). We also identified three PEPC genes in the tung tree genome that were highly expressed in the early developmental stages of tung seed. In contrast, the soybean harbors 16 *PEPC* genes and other species harbor more PEPC genes than tung tree (Table S38). Because soybean has more PEPC genes and higher protein/lower oil content of the seed than tung tree, it is possible that the fewer PEPC genes present in tung tree divert relatively less carbon flow toward protein biosynthesis than in soybean, with a resultant high oil/low protein content of tung seed.

Tung oil is the major economically important product from tung tree. Identification and characterization of tung oil biosynthesis genes is essential for improving tung oil production and its economic value. Interestingly, we identified an additional *FADX* gene in the tung tree genome, *FADX-2*, which might be generated by gene duplication and then undergo sub-functionalization, based on the different expression patterns of *FADX-1* and *FADX-2*. In comparison with *FADX-2*, *FADX-1* was the dominant form responsible for α-ESA synthesis in the developing seed of tung tree. We also identified nine ACCase, four DGAT, seven FAD, six PDAT, five OLE, eight LACS, nine GPAT, seven LPAT, and six PP genes in the tung tree genome. The current study has thus provided a more complete picture of genes involved in tung oil biosynthesis than previously achieved. The number of tung oil-synthesizing genes is comparable to that in other species except that soybean has many more genes (Table S38). This suggests a lack of expansion of these genes in tung tree. Therefore, the amount and types of oils produced in various species may not be directly related to the number of genes involved in oil biosynthesis.

We also used transcriptomic analysis to evaluate the expression profiles of all the aforementioned genes. The analysis indicated that the expression patterns of some of the most important genes are well coordinated with oil biosynthesis and accumulation in tung tree seed. Specifically, *DGAT2* was the most highly expressed *DGAT* gene in tung seed, but *DGAT3-1* was the dominant form of *DGAT* in immature seed and other tissues including the stem, root, leaf, and female flower, which is in agreement with our previous studies [14], [18]. *FAD2* and *FADX* were highly expressed at mid-late stages of seed development, whereas *FAD3* was most highly expressed at the early stages of seed development, which is also in agreement with published results [15]. All (five) *OLE* genes were highly expressed at mid-late stage of tung seed development, similar to our previous findings [30]. The expression analysis provides novel insights into the potential role of *PDAT* genes in tung oil biosynthesis. It was revealed that *PDAT1-1*, *1*-*4*, and *2*-*2* were highly expressed at mid-late stages of seed development, with the other three *PDAT* genes highly expressed at the early stages of seed development, an observation that has not been reported before. Gene co-expression analysis in the current study revealed that oil biosynthesis-related genes were enriched in two significant modules only at 20 WAF when seed oil starts to accumulate rapidly. The enriched oil biosynthesis-related genes include most of *FAS* genes, some of TAG biosynthesis genes, and some transcription factor genes. The complete gene co-expression networks provide insights into oil biosynthesis by revealing gene-gene synergistic functions.

In conclusion, the current study provides whole-genome sequence information, eFP browser, and extensive RNA-seq data for tung tree. These critical pieces of information should be useful as valuable resources for functional genomics studies and tree improvement of economically important traits, such as oil content and disease resistance in the tung tree.

## Materials and methods

### Plant material

The self-bred progeny VF1-12 of the elite *V. fordii* cv. Putaotong was used for whole-genome sequencing in the current study (File S1). Young leaves were collected from VF1-12 in the spring for genome sequencing. Young plantlets were used for Hi-C library construction and sequencing. For the study, 17 fresh tissues, including the stem, root, male flower, female flower, and seed, at different developmental stages were collected for RNA-seq. The developing seeds were also used for oil content determinations and fatty acid analysis.

### Whole-genome sequencing, assembly, and assessment

The tung tree genome size was determined by using modified Lander-Waterman algorithm, *i.e.*, a formula G = Bnum/Bdepth = Knum/Kdepth [45]. Heterozygosity was determined by the k-mer distribution and GenomeScope [46]. Nuclear DNA was isolated from fresh leaf tissues by using a DNeasy Plant Mini kit (catalog No. CA69104, Qiagen, Dusseldorf, Germany). A series of DNA libraries were constructed and sequenced using an Illumina HiSeq 2000 sequencing platform (Illumina, San Diego, CA) (File S9). In addition, SMRTbell template libraries of 20 kb were constructed and sequenced using the PacBio RSII. After removing low-quality reads, the whole-genome assembly of tung tree was performed using a hierarchical assembly strategy because of the homozygosity of the genome and the presence of highly repetitive sequences (File S10). The genome completeness was assessed by CEGMA [47], BUSCO analysis [48], and RNA-seq read mapping [49].

### Hi-C data preparation and contig clustering

The Hi-C library was prepared using standard procedures [50]. Raw Hi-C data were generated using HiSeq2500 sequencing platform (Illumina) and then were processed to filter low-quality reads and trim adapters. Clean reads were mapped to the assembled scaffolds by using BWA-aln after truncating the putative Hi-C junctions in sequence reads [51]. HiC-Pro software (version 2.7.1) was used to filter invalid ligation read pairs, including dangling ends, as well as self-ligation, re-ligation, and dumped products. Finally, the scaffolds were clustered, ordered, and orientated onto the chromosomes using the valid read pairs by LACHESIS (http://shendurelab.github.io/LACHESIS/).

### Genome annotation

Gene prediction was conducted using *de novo* prediction, homology information, and RNA-seq data (File S11). Gene functions were assigned according to the best match derived from the alignments to proteins annotated in SWISS-PROT and TrEMBL databases using Blastp, and the pathway in which the gene might be involved was annotated by KAAS [52]. Motifs and domains were annotated using InterProScan (Version 5.2-45.0) [53], by searching against publicly available databases in InterPro [54]. The rRNA, snRNA, and miRNA genes were predicted by Infernal software using the Rfam database. The rRNA subunits were identified by RNAmmer [55], based on hidden Markov models. The tRNA genes were predicted with tRNAscan-SE [56] by applying eukaryote parameters. A *de novo* and homology-based approach was used to identify repetitive sequence and transposable elements in the tung tree genome.

### Evolutionary analysis

Phylogeny of eight species (*V. fordii*, *A. thaliana*, *V. vinifera*, *P. trichocarpa*, *J. curcas*, *R. communis*, *M. esculenta*, and *H. brasiliensis*) was constructed based on single-copy gene families by using the maximum likelihood method (File S12). Genome sequences of *V. vinifera* (Genoscope.12X), *P. trichocarpa* (v3.0), *R. communis* (v0.1), and *M. esculenta* (v6.1) were downloaded from Phytozome v10 Database (http:phytozome.jgi.doe.gov/pz/portal.html). Genome sequences of *A. thaliana*, *J. curcas*, and *H. brasiliensis* were downloaded from the TAIR10 website (https://www.arabidopsis.org/index.jsp), Jatropha Genome Database (http://www.kazusa.or.jp/jatropha/), and Rtg database (http://www.4a.biotec.or.th/rubber/), respectively. The divergence times were estimated based on all single-copy genes and 4-fold degenerate sites using the program MCMCTree in the PAML package [57]. The neutral evolutionary rate was calculated by Bayes estimation with Markov Chain Monte Carlo algorithm. Gene families that underwent expansion or contraction were identified using the Computational Analysis of gene Family Evolution (CAFÉ) program [58]. The selection pressure on tung tree in the phylogenetic tree was calculated by CodeML. The significance of the identified PSGs was verified using the Chi-square test. WGD events were identified by 4DTv and synonymous Ks analysis.

## Data availability

The data of genome sequencing, Hi-C and RNA-seq of tung tree have been deposited in the Genome Sequence Archive [59] at the BIG Data Center, Beijing Institute of Genomics (BIG), Chinese Academy of Sciences (GSA:CRA001732), and are publicly accessible at https://bigd.big.ac.cn/gsa/. These data have also been deposited in NCBI (BioProject: PRJNA503685, PRJNA445350, and PRJNA483508).

## Authors’ contributions

XT and LZ conceived and supervised the project. XT, LZ, and HPC conceived the idea and designed the study. XT, LZ, HL, ML, ZL, YZ, and HC prepared the experimental materials. HL, ML, LZ, HPC, LSZ, AS, DR, GZ, MZ, JL, FL, JH, DW, MX, XY, and WD performed the data analysis. AP, EE, WL, and NJP performed eFP browser construction. WL and ML designed and drew the figures. LZ and HPC drafted the manuscript; LZ, HPC, NJP, and LSZ revised the manuscript. All authors read and approved the final manuscript.

## Competing interests

The authors have declared no competing interests.

## References

[1] Liu M., Li W., Zhao G., Fan X., Long H., Fan Y. (2019). New insights of salicylic acid into stamen abortion of female flowers in tung tree (*Vernicia fordii*). Front Genet.

[2] Zhang L., Jia B., Tan X., Thammina C.S., Long H., Liu M. (2014). Fatty acid profile and unigene-derived simple sequence repeat markers in tung tree (*Vernicia fordii*). PLoS One.

[3] Liu M., Long H., Li W., Shi M., Cao H., Zhang L. (2019). Boosting C16 fatty acid biosynthesis of Escherichia coli, yeast and tobacco by Tung tree (*Vernicia fordii* Hemsl.) beta-hydroxyacyl-acyl carrier protein dehydratase gene. Ind Crop Prod.

[4] Huang Y., Pang L., Wang H., Zhong R., Zeng Z., Yang J. (2013). Synthesis and properties of UV-curable tung oil based resins via modification of Diels-Alder reaction, nonisocyanate polyurethane and acrylates. Prog Org Coat.

[5] Liu C., Shang Q., Jia P., Dai Y., Zhou Y., Liu Z. (2016). Tung oil-based unsaturated co-ester macromonomer for thermosetting polymers: synergetic synthesis and copolymerization with styrene. ACS Sustain Chem Eng.

[6] Park J.Y., Kim D.K., Wang Z.M., Lu P., Park S.C., Lee J.S. (2008). Production and characterization of biodiesel from tung oil. Appl Biochem Biotechnol.

[7] Shang Q., Lei J., Jiang W., Lu H., Liang B. (2012). Production of tung oil biodiesel and variation of fuel properties during storage. Appl Biochem Biotechnol.

[8] Chen Y.H., Chen J.H., Luo Y.M. (2012). Complementary biodiesel combination from tung and medium-chain fatty acid oils. Renew Energy.

[9] Meininghaus R., Gunnarsen L., Knudsen H.N. (2000). Diffusion and sorption of volatile organic compounds in building materials-impact on indoor air quality. Environ Sci Technol.

[10] Tsakas M.P., Siskos A.P., Siskos P.A. (2011). Indoor air pollutants and the impact on human health, chemistry, emission control, radioactive pollution and indoor air quality. InTech.

[11] Wei W., Zhang Y., Xiong J., Li M. (2012). A standard reference for chamber testing of material VOC emissions: design principle and performance. Atmos Environ.

[12] Yang X., Zhang S., Li W. (2015). The performance of biodegradable tung oil coatings. Prog Org Coat.

[13] Yoo Y., Youngblood J.P. (2017). Tung oil wood finishes with improved weathering, durability, and scratch performance by addition of cellulose nanocrystals. ACS Appl Mater Interfaces.

[14] Shockey J.M., Gidda S.K., Chapital D.C., Kuan J.C., Dhanoa P.K., Bland J.M. (2006). Tung tree DGAT1 and DGAT2 have nonredundant functions in triacylglycerol biosynthesis and are localized to different subdomains of the endoplasmic reticulum. Plant Cell.

[15] Dyer J.M., Chapital D.C., Kuan J.C., Mullen R.T., Turner C., McKeon T.A. (2002). Molecular analysis of a bifunctional fatty acid conjugase/desaturase from tung. Implications for the evolution of plant fatty acid diversity. Plant Physiol.

[16] Shockey J.M., Dhanoa P.K., Dupuy T., Chapital D.C., Mullen R.T., Dyer J.M. (2005). Cloning, functional analysis, and subcellular localization of two isoforms of NADH:cytochrome b5 reductase from developing seeds of tung (*Vernicia fordii*). Plant Sci.

[17] Cao H., Chapital D.C., Howard O.D., Deterding L.J., Mason C.B., Shockey J.M. (2012). Expression and purification of recombinant tung tree diacylglycerol acyltransferase 2. Appl Microbiol Biotechnol.

[18] Cao H., Shockey J.M., Klasson K.T., Chapital D.C., Mason C.B., Scheffler B.E. (2013). Developmental regulation of diacylglycerol acyltransferase family gene expression in tung tree tissues. PLoS One.

[19] Li L., Stoeckert C.J., Roos D.S. (2003). OrthoMCL: identification of ortholog groups for eukaryotic genomes. Genome Res.

[20] Zhang L., Li X., Ma B., Gao Q., Du H., Han Y. (2017). The tartary buckwheat genome provides insights into rutin biosynthesis and abiotic stress tolerance. Mol Plant.

[21] Tang C., Yang M., Fang Y., Luo Y., Gao S., Xiao X. (2016). The rubber tree genome reveals new insights into rubber production and species adaptation. Nat Plants.

[22] Chan A.P., Crabtree J., Zhao Q., Lorenzi H., Orvis J., Puiu D. (2010). Draft genome sequence of the oilseed species *Ricinus communis*. Nat Biotechnol.

[23] Wu P., Zhou C., Cheng S., Wu Z., Lu W., Han J. (2015). Integrated genome sequence and linkage map of physic nut (*Jatropha curcas* L.), a biodiesel plant. Plant J.

[24] Wang L., Yu S., Tong C., Zhao Y., Liu Y., Song C. (2014). Genome sequencing of the high oil crop sesame provides insight into oil biosynthesis. Genome Biol.

[25] Pootakham W., Sonthirod C., Naktang C., Ruang-Areerate P., Yoocha T., Sangsrakru D. (2017). *De novo* hybrid assembly of the rubber tree genome reveals evidence of paleotetraploidy in *Hevea* species. Sci Rep.

[26] Wu J., Wang Z., Shi Z., Zhang S., Ming R., Zhu S. (2013). The genome of the pear (*Pyrus bretschneideri* Rehd.). Genome Res.

[27] Chen J., Lang C., Hu Z., Liu Z., Huang R. (1999). Antisense PEP gene regulates to ratio of and protein and lipid content in *Brassica napus* seeds. J Agr Biotechnol.

[28] Pan X., Peng F.Y., Weselake R.J. (2015). Genome-Wide analysis of phospholipid: diacylglycerol acyltransferase (PDAT) genes in plants reveals the eudicot-wide PDAT gene expansion and altered selective pressures acting on the core eudicot PDAT paralogs. Plant Physiol.

[29] Cao H., Zhang L., Tan X., Long H., Shockey J.M. (2014). Identification, classification and differential expression of oleosin genes in tung tree (*Vernicia fordii*). PLoS One.

[30] Baud S., Wuilleme S., To A., Rochat C., Lepiniec L. (2009). Role of WRINKLED1 in the transcriptional regulation of glycolytic and fatty acid biosynthetic genes in Arabidopsis. Plant J.

[31] Sugliani M., Rajjou L., Clerkx E.J.M., Koornneef M., Soppe W.J.J. (2009). Natural modifiers of seed longevity in the *Arabidopsis* mutants *abscisic acid insensitive3-5 (abi3-*5) and *leafy cotyledon1-*3 *(lec1*-3). New Phytol.

[32] Kirkbride R.C., Fischer R.L., Harada J.J. (2013). *LEAFY COTYLEDON1*, a key regulator of seed development, is expressed in vegetative and sexual propagules of *Selaginella moellendorffii*. PLoS One.

[33] Rikiishi K., Maekawa M. (2014). Seed maturation regulators are related to the control of seed dormancy in wheat (*Triticum aestivum* L.). PLoS One.

[34] Huang M., Hu Y., Liu X., Li Y., Hou X. (2015). *Arabidopsis* LEAFY COTYLEDON1 controls cell fate determination during post-embryonic development. Front Plant Sci.

[35] Xia E.H., Zhang H.B., Sheng J., Li K., Zhang Q.J., Kim C. (2017). The tea tree genome provides insights into tea flavor and independent evolution of caffeine biosynthesis. Mol Plant.

[36] Guan R., Zhao Y., Zhang H., Fan G., Liu X., Zhou W. (2016). Draft genome of the living fossil *Ginkgo biloba*. GigaScience.

[37] Nystedt B., Street N.R., Wetterbom A., Zuccolo A., Lin Y.C., Scofield D.G. (2013). The Norway spruce genome sequence and conifer genome evolution. Nature.

[38] Patel R.V., Nahal H.K., Breit R., Provart N.J. (2012). BAR expressolog identification: expression profile similarity ranking of homologous genes in plant species. Plant J.

[39] Winter D., Baxter I., Vinegar B., Nahal H., Ammar R., Wilson G.V. (2007). An “Electronic Fluorescent Pictograph” browser for exploring and analyzing large-scale biological data sets. PLoS One.

[40] Kagale S., Nixon J., Khedikar Y., Pasha A., Provart N.J., Clarke W.E. (2016). The developmental transcriptome atlas of the biofuel crop *Camelina sativa*. Plant J.

[41] Hawkins C., Caruana J., Li J., Zawora C., Darwish O., Wu J. (2017). An eFP browser for visualizing strawberry fruit and flower transcriptomes. Hortic Res.

[42] Meyers B.C., Kozik A., Griego A., Kuang H., Michelmore R.W. (2003). Genome-wide analysis of NBS-LRR-encoding genes in *Arabidopsis*. Plant Cell.

[43] Liu J., Liu X., Dai L., Wang G. (2007). Recent progress in elucidating the structure, function and evolution of disease resistance genes in plants. J Genet Genomics.

[44] Gassmann W., Hinsch M.E., Staskawicz B.J. (1999). The *Arabidopsis* RPS4 bacterial-resistance gene is a member of the TIR-NBS-LRR family of disease-resistance genes. Plant J.

[45] Wendl M.C., Barbazuk W.B. (2005). Extension of Lander-Waterman theory for sequencing filtered DNA libraries. BMC Bioinformatics.

[46] Vurture G.W., Sedlazeck F.J., Nattestad M., Underwood C.J., Fang H., Gurtowski J. (2017). GenomeScope: fast reference-free genome profiling from short reads. Bioinformatics.

[47] Parra G., Bradnam K., Korf I. (2007). CEGMA: a pipeline to accurately annotate core genes in eukaryotic genomes. Bioinformatics.

[48] Simão F.A., Waterhouse R.M., Ioannidis P., Kriventseva E.V., Zdobnov E.M. (2015). BUSCO: assessing genome assembly and annotation completeness with single-copy orthologs. Bioinformatics.

[49] Trapnell C., Pachter L., Salzberg S.L. (2009). TopHat: discovering splice junctions with RNA-Seq. Bioinformatics.

[50] Lieberman-Aiden E., van Berkum N.L., Williams L., Imakaev M., Ragoczy T., Telling A. (2009). Comprehensive mapping of long-range interactions reveals folding principles of the human genome. Science.

[51] Li H., Durbin R. (2009). Fast and accurate short read alignment with Burrows-Wheeler transform. Bioinformatics.

[52] Moriya Y., Itoh M., Okuda S., Yoshizawa A.C., Kanehisa M. (2007). KAAS: an automatic genome annotation and pathway reconstruction server. Nucleic Acids Res.

[53] Zdobnov E.M., Apweiler R. (2001). InterProScan–an integration platform for the signature-recognition methods in InterPro. Bioinformatics.

[54] Hunter S., Apweiler R., Attwood T.K., Bairoch A., Bateman A., Binns D. (2009). InterPro: the integrative protein signature database. Nucleic Acids Res.

[55] Lagesen K., Hallin P., Rødland E.A., Stærfeldt H.H., Rognes T., Ussery D.W. (2007). RNAmmer: consistent and rapid annotation of ribosomal RNA genes. Nucleic Acids Res.

[56] Lowe T.M., Eddy S.R. (1997). tRNAscan-SE: a program for improved detection of transfer RNA genes in genomic sequence. Nucleic Acids Res.

[57] Yang Z. (2007). PAML 4: phylogenetic analysis by maximum likelihood. Mol Biol Evol.

[58] De Bie T., Cristianini N., Demuth J.P., Hahn M.W. (2006). CAFE: a computational tool for the study of gene family evolution. Bioinformatics.

[59] Wang Y., Song F., Zhu J., Zhang S., Yang Y., Chen T. (2017). GSA: genome sequence archive. Genomics Proteomics Bioinformatics.

