# Supplementary material for: Tung Tree (Vernicia fordii) Genome Provides A Resource for Understanding Genome Evolution and Improved Oil Production
Source: Genomics Proteomics Bioinformatics. 2020 Mar 26;17(6):558–75. doi: 10.1016/j.gpb.2019.03.006 (PMC7212303; doi:10.1016/j.gpb.2019.03.006)
Supplement: Supplementary data 1 [file mmc1.docx]

**File S1 Self-pollination and genome survey**

To obtain plants with low heterozygosity for whole genome sequencing, we conducted self-pollination of an elite tung tree (*Vernicia fordii*) cv. Putaotong at Central South University of Forestry and Technology Germplasm Repository (Yongshun, Hunan). A five-year-old tree was used for control pollination in April, 2012 and a total of 258 seeds were obtained and finally 61 seedlings were produced. The 61 self-bred progenies of ‘Putaotong’ were then used for heterozygosity estimation by 27 SSR markers including 20 genomic SSR markers from Xu et al. [1] and 7 EST-SSR markers from Jia and colleagues [2]. Consequently, 15 progenies (‘VFPPT1-1’, ‘VFPPT1-4’, ‘VFPPT1-5’, ‘VFPPT1-9’, ‘VFPPT1-12’, ‘VFPPT2-16’, ‘VFPPT2-17’, ‘VFPPT3-2’, ‘VFPPT3-3’, ‘VFPPT4-3’, ‘VFPPT4-6’, ‘VFPPT5-3’, ‘VFPPT5-4’, ‘VFPPT5-5’, and ‘VFPPT5-7’) were found to harbor most homozygous loci (25 in each plant) and preliminarily estimated to be low heterozygosity. The ‘VF1-12’ was used for genome survey and whole genome sequencing. A total of 36.51 Gb data from a library of short insert sizes (500 bp) was used to estimate the genome size by a modified Lander-Waterman algorithm i.e. a formula G =Bnum/Bdepth = Knum/Kdepth [3]. In this formula, G, Bnum, Bdepth, Knum, and Kdepth refer to genome size, the total read number, the depth of bases, the total number of K-mer, and the overall depth of K-mer, respectively. Knum is calculated by N × (L – K + 1) where N, L, and K represent the number of K-mer, the length of reads, and the size of K-mer, respectively. A single peak indicated that the sample genome harbored low heterozygosity level. The heterozygous rate was estimated with GenomeScope [4].

**References**

[1] Xu W, Yang Q, Huai H, Liu A. Microsatellite marker development in tung trees (*Vernicia montana* and *V. fordii*, Euphorbiaceae). Am J Bot 2011;98:e226−8.

[2] Jia B, Lin Q, Tan X, Li Z, Long H, Xiang H, et al. Development of EST-SSR markers and their use for genetic diversity analysis in tung tree (*Vernicia fordii* (Hemsl.) Airy Shaw). J Plant Genet Resources 2016;17:625−36.

[3] Wendl MC, Barbazuk WB. Extension of Lander-Waterman theory for sequencing filtered DNA libraries. BMC Bioinformatics 2005;6:245.

[4] Vurture GW, Sedlazeck FJ, Nattestad M, Underwood CJ, Fang H, Gurtowski J, et al. GenomeScope: fast reference-free genome profiling from short reads. Bioinformatics 2017;33:2202−4.


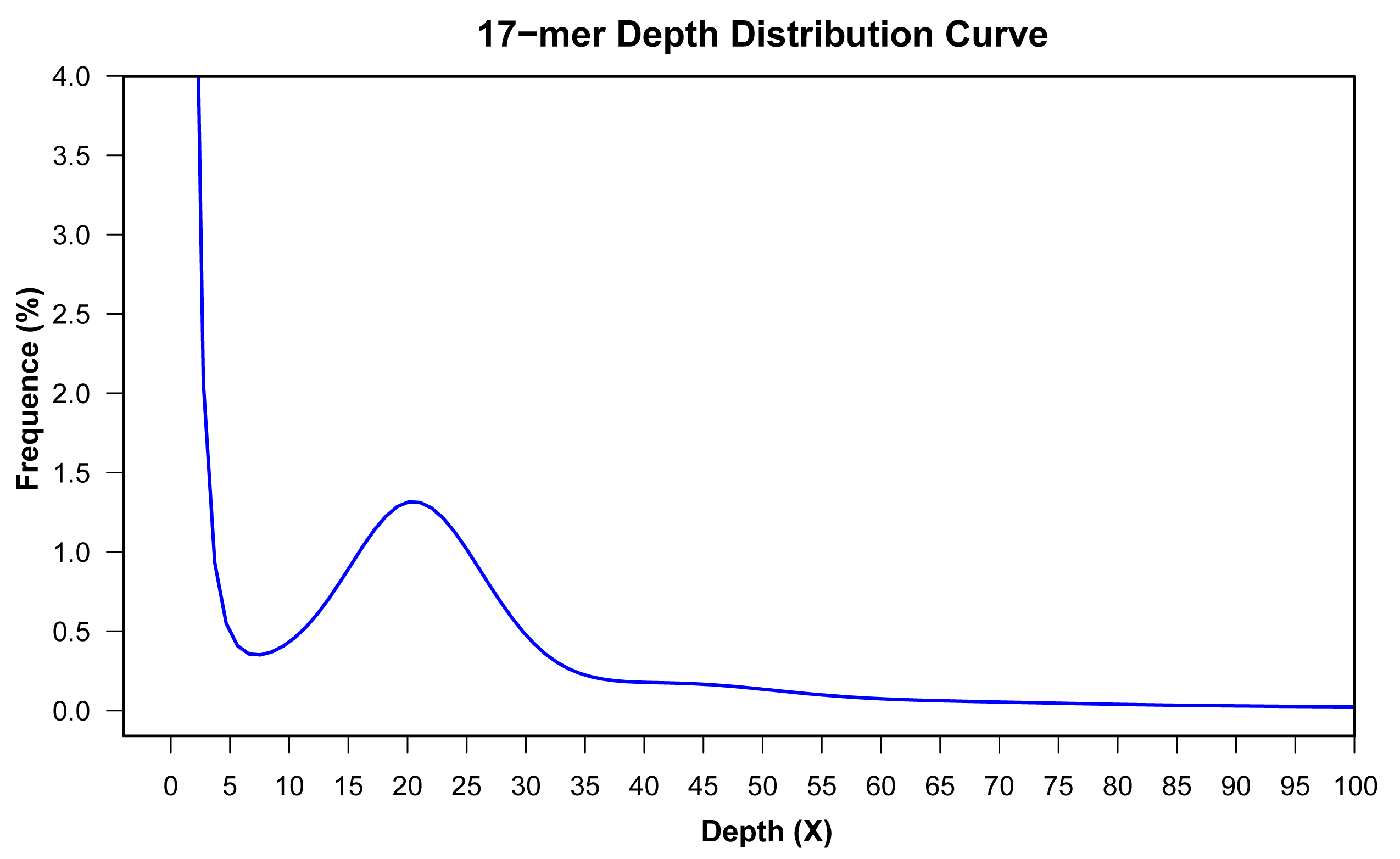


**Figure S1 The k-mer analysis to estimate the tung tree genome size**

The figure shows frequency of 17 k-mers which are 17 bp sequences from the reads (after filtering) of short-insert size libraries. We identified 30,007,782,380 k-mers using 36.51 Gb data. The tung tree genome size was estimated to be 1.31 Gb by (total k-mer number)/(the volume peak).
