# Supplementary material for: Tung Tree (Vernicia fordii) Genome Provides A Resource for Understanding Genome Evolution and Improved Oil Production
Source: Genomics Proteomics Bioinformatics. 2020 Mar 26;17(6):558–75. doi: 10.1016/j.gpb.2019.03.006 (PMC7212303; doi:10.1016/j.gpb.2019.03.006)
Supplement: Supplementary data 2 [file mmc2.docx]

**File S2 Repeat sequence analysis**

A *de novo* and homology-based approach was used to identify repetitive sequence and transposable elements (TEs) in the tung tree genome. A *de novo* repeat library by Repeat Modeler was constructed to generate the consensus sequences and classification information for each repeat family [1]. Repeat Masker was then applied for DNA-level identification using the *de novo* library and two databases, Repbase [2] and Mips-Redat [3]. At the protein level, Repeat Protein Mask was used to perform WU-BLATX against the TE protein database. The overlapping TEs belonging to the same type of repeats were collated and combined according to their coordinates in the genome. Tandem repeats were annotated with TRF (Tandem Repeat Finder) [4].

**Identification of simple sequence repeats (SSRs)**

We searched the simple sequence repeats (SSRs) from the tung tree genome sequences by using MIcroSAtellite Identification Tool (MISA). We identified 663,931 SSRs with the density of 593.49 SSRs per Mb across the whole tung tree genome, of which compound format SSRs were 172,997, accounting for 26.06%. The minimum repeat unit size for mononucleotide was set at ten, for dinucleotide at six, and for tri-, tetra-, penta-, and hexa-nucleotide at five. In total, we identified six types of SSRs including mono-, di-, tri-, tetra-, penta-, and hexa-nucleotide. The annotated SSRs were mostly mononucleotide (263,069; 39.62%), and dinucleotide (88,805; 13.38%), and less trinucleotide (36,459; 5.49%), tetranucleotide (4884; 0.74%), and pentanucleotide (2073; 0.31%), and hexanucleotide (639; 0.10%). These SSRs will provide valuable genetic markers to assist tung tree breeding programs.

**Intact LTR-RT identification and insertion time estimation**

The long-terminal repeat retrotransposons (LTR-RTs) in the whole tung tree genome were identified with LTR-finder software. To classify the types of LTR-RTs, the repeat regions were masked using RepeatMasker and the LTR-RTs were located to RepeatMasker results. LTR insertion time was estimated by computing the nucleotide substitution rate of the two LTRs in an intact LTR-RT since they were assumed to be identical at the retroelement insertion time [5]. LTRs in each pair were aligned to calculate the substitution rate by the baseml algorithm of the PAML package with the TN93 model. The insertion time was counted by the formula of T=*K*/2r. T: element insertion time; r: synonymous mutation/site/year; *K*: the divergence between the LTRs and consensus sequence in the TE library. The substitution rate was 1.3×10^-8^ substitutions per site per year, 2-fold higher than the synonymous substitution rate of the coding region (6.5×10^-9^).

All intact LTR retrotransposons were classified into families by using BLASTClust and all-to-all BLAST of 5′ LTR sequences, followed by manual inspection [6]. The family classification standard was considered acceptable if more than 50% of the 5′ LTR mapped and sequence identify exceeded 80%. As a result, we classified 2991 intact retrotransposon elements into a total of 1130 families which included 89 multi-member families (≥5 intact members), 154 median-copy families (2−4 intact members), and 887 single-copy families (Table S26).

All intact LTR retrotransposons were classified into *Ty1/Copia*, *Ty3/Gypsy* and unclassified groups according to both RT gene similarity and the order of ORFs using PFAM [7]. The RT sequences were retrieved from each retrotransposon element and further checked by homology searches using ClustalW [8] against the published RTs that were downloaded from the Gypsy Database (GyDB) [9].

**Phylogenetic analysis of LTR retrotransposon families**

We extracted nucleotide sequences of RTs from intact LTR retrotransposon elements and conducted alignments of amino acid sequences of RTs using ClustalW2 [8]. Consequently, unrooted neighbor-joining (NJ) phylogenetic trees were generated by using MEGA 6 [10]. In total, 347 were grouped into *Ty1*/*Copia* families, including Ale, Angela, Bianca, Horpia2, Lkya, Owis, Rare1, and Rare2, of which Ale was most abundant (115) accounting for 33.14% (Figure 3A and B; Table S43). A total of 622 were *Ty3*/*Gypsy* sequences, including Bagy2, Cereba, Dagan, Erika, GA, Geneva, Laura, and Retrosat, of which Dagan was most abundant (373) accounting for 59.97% (Figure 3A and B; Table S27).

**Expression of LTR retrotransposons**

To understand the activation of the most abundant LTR retrotransposons in the tung tree genome, expression levels of LTR retrotransposons were estimated by computing the number of reads in LTR retrotransposons within RNA-Seq datasets from six tissues including 10 samples (root, stem, young leaf, female flower, male flower, and seed at five developing stages) generated in this study. Transcriptome data from five seed samples were normalized to be used for expression calculation. The transcriptomes were masked by RepeatMasker (version 4.0.5) using the same TE library as used for annotation of the tung tree genome. TopHat2 [11] (version 2.0.5) and StringTie (version 1.3.0) [12] were used to count the number of reads in RNA-Seq data (Table S28).

Based on our RNA-seq data, we found 1738 out of the total 2991 LTR retrotransposons exhibited expression across six tissues and different LTR retrotransposon families exhibited distinct expression patterns. Generally, *Ty3/Gypsy* LTR retrotransposons exhibited higher expression levels than *Ty1*/*Copia* retrotransposons, ranging from 0.71-fold in seed to 4.09-fold in leaf with approximately two-fold higher on average (Table S28). Among the 1738 LTR retrotransposons, 701 showed the highest expression level in seeds, of which 60.77% belongs to high-copy families (Figure 3D). However, VL0631, a single-copy LTR retrotransposon, was the most highly expressed in seeds (Table S29). In the top 29 LTR retrotransposons highly expressed in seeds (FMKM value ≥ 1), high-copy families, median-copy families and sing-copy families are 3, 6, and 20, accounting for 10.34%, 20.69%, and 68.97%, respectively (Table S29). In addition, 184, 204, 244, 148, and 257 LTR retrotransposons exhibited the highest expression levels in root, stem, leaf, female flower and male flower, respectively (Figure S10). Among these genes, similar to seeds, high-copy LTR families also accounted for the highest proportion in the other five tissues and single-copy families accounted for the highest proportion in the most highly expressed LTR retrotransposons (Tables S30−S34; Figure S33).

**References**

[1] Bedell JA, Korf I, Gish W. MaskerAid a performance enhancement to RepeatMasker. Bioinformatics 2000;16:1040−1.

[2] Jurka J, Kapitonov VV, Pavlicek A, Klonowski P, Kohany O, Walichiewicz J. Repbase Update, a database of eukaryotic repetitive elements. Cytogenet Genome Res 2005;110:462−7.

[3] Spannagl M, Noubibou O, Haase D, Yang L, Gundlach H, Hindemitt T, et al. MIPSPlantsDB--plant database resource for integrative and comparative plant genome research. Nucleic Acids Res 2007;35:D834−40.

[4] Benson G. Tandem repeats finder: a program to analyze DNA sequence. Nucleic Acids Res 1999;27:573.

[5] SanMiguel P, Gaut BS, Tikhonov A, Nakajima Y, Bennetzen JL. The paleontology of intergene retrotransposons of maize. Nat Genet 1998;20:43−5.

[6] Llorens C, Futami R, Covelli L, Dominguez-Escriba L, Viu JM, Tamarit D, et al. The Gypsy Database (GyDB) of mobile genetic elements: release 2.0. Nucleic Acids Res 2011;39:D70−4.

[7] Finn RD, Coggill P, Eberhardt RY, Eddy SR, Mistry J, Mitchell AL, et al. The Pfam protein families database: towards a more sustainable future. Nucleic Acids Res 2015;44:D279−85.

[8] Larkin MA, Blackshields G, Brown NP, Chenna R, McGettigan PA, McWilliam H, et al. Clustal W and Clustal X version 2.0. Bioinformatics 2007;23:2947−8.

[9] Krzywinski M, Schein J, Birol I, Connors J, Gascoyne R, Horsman D, et al. Circos: an information aesthetic for comparative genomics. Genome Res 2009;19:1639−45.

[10] Tamura K, Stecher G, Peterson D, Filipski A, Kumar S. MEGA6: Molecular evolutionary genetics analysis version 6.0. Mol Biol Evol 2013;30:2725−9.

[11] Kim D, Langmead B, Salzberg SL. HISAT: a fast spliced aligner with low memory requirements. Nat Methods 2015;12:357−60.

[12] Pertea M, Pertea GM, Antonescu CM, Chang T-C, Mendell JT, Salzberg SL. StringTie enables improved reconstruction of a transcriptome from RNA-seq reads. Nat Biotechnol 2015;33:290−5.


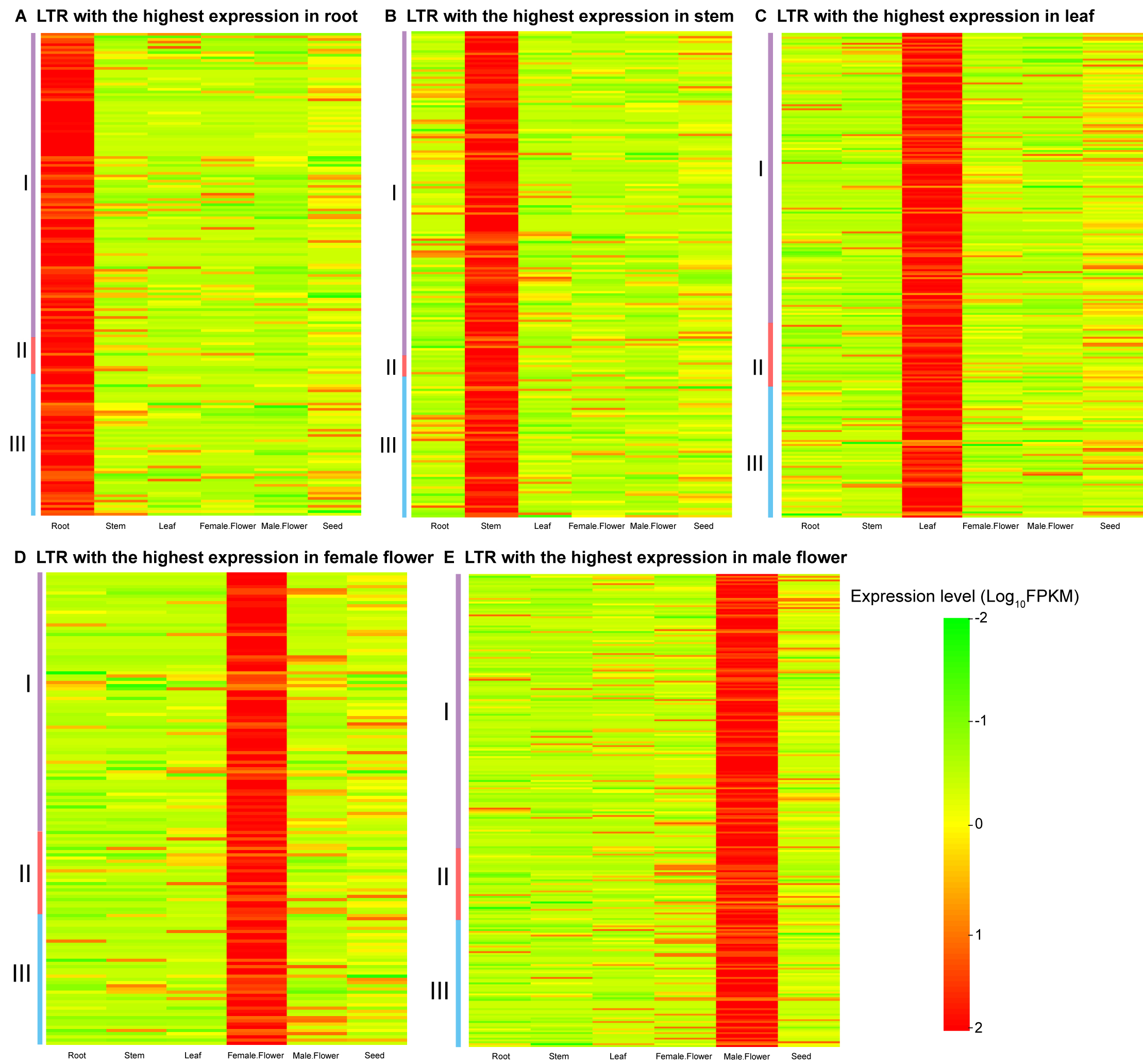


**Figure S10 Heat map of expression patterns of tung tree LTR retrotransposons**

**A.−E.** indicates LTR retrotransposons showing the highest expression level in root, stem, leaf, female flower, and male flower, respectively

**Table S26 Classification of LTR families**

| **LTR families** | **Copy number** | **Proportion (%)** |
| --- | --- | --- |
| VL0001 | 130 | 4.346372451 |
| VL0002 | 117 | 3.911735206 |
| VL0003 | 114 | 3.811434303 |
| VL0004 | 75 | 2.507522568 |
| VL0005 | 70 | 2.340354397 |
| VL0006 | 70 | 2.340354397 |
| VL0007 | 64 | 2.139752591 |
| VL0008 | 61 | 2.039451688 |
| VL0009 | 61 | 2.039451688 |
| VL0010 | 46 | 1.537947175 |
| VL0011 | 42 | 1.404212638 |
| VL0012 | 42 | 1.404212638 |
| VL0013 | 37 | 1.237044467 |
| VL0014 | 37 | 1.237044467 |
| VL0015 | 37 | 1.237044467 |
| VL0016 | 32 | 1.069876296 |
| VL0017 | 32 | 1.069876296 |
| VL0018 | 30 | 1.003009027 |
| VL0019 | 23 | 0.768973587 |
| VL0020 | 21 | 0.702106319 |
| VL0021 | 20 | 0.668672685 |
| VL0022 | 20 | 0.668672685 |
| VL0023 | 18 | 0.601805416 |
| VL0024 | 18 | 0.601805416 |
| VL0025 | 17 | 0.568371782 |
| VL0026 | 17 | 0.568371782 |
| VL0027 | 16 | 0.534938148 |
| VL0028 | 15 | 0.501504514 |
| VL0029 | 14 | 0.468070879 |
| VL0030 | 13 | 0.434637245 |
| VL0031 | 13 | 0.434637245 |
| VL0032 | 13 | 0.434637245 |
| VL0033 | 13 | 0.434637245 |
| VL0034 | 12 | 0.401203611 |
| VL0035 | 12 | 0.401203611 |
| VL0036 | 12 | 0.401203611 |
| VL0037 | 12 | 0.401203611 |
| VL0038 | 10 | 0.334336342 |
| VL0039 | 9 | 0.300902708 |
| VL0040 | 9 | 0.300902708 |
| VL0041 | 9 | 0.300902708 |
| VL0042 | 9 | 0.300902708 |
| VL0043 | 8 | 0.267469074 |
| VL0044 | 8 | 0.267469074 |
| VL0045 | 8 | 0.267469074 |
| VL0046 | 8 | 0.267469074 |
| VL0047 | 8 | 0.267469074 |
| VL0048 | 7 | 0.23403544 |
| VL0049 | 7 | 0.23403544 |
| VL0050 | 7 | 0.23403544 |
| VL0051 | 7 | 0.23403544 |
| VL0052 | 7 | 0.23403544 |
| VL0053 | 7 | 0.23403544 |
| VL0054 | 6 | 0.200601805 |
| VL0055 | 6 | 0.200601805 |
| VL0056 | 6 | 0.200601805 |
| VL0057 | 6 | 0.200601805 |
| VL0058 | 6 | 0.200601805 |
| VL0059 | 6 | 0.200601805 |
| VL0060 | 6 | 0.200601805 |
| VL0061 | 6 | 0.200601805 |
| VL0062 | 6 | 0.200601805 |
| VL0063 | 6 | 0.200601805 |
| VL0064 | 6 | 0.200601805 |
| VL0065 | 6 | 0.200601805 |
| VL0066 | 6 | 0.200601805 |
| VL0067 | 6 | 0.200601805 |
| VL0068 | 6 | 0.200601805 |
| VL0069 | 6 | 0.200601805 |
| VL0070 | 6 | 0.200601805 |
| VL0071 | 6 | 0.200601805 |
| VL0072 | 6 | 0.200601805 |
| VL0073 | 5 | 0.167168171 |
| VL0074 | 5 | 0.167168171 |
| VL0075 | 5 | 0.167168171 |
| VL0076 | 5 | 0.167168171 |
| VL0077 | 5 | 0.167168171 |
| VL0078 | 5 | 0.167168171 |
| VL0079 | 5 | 0.167168171 |
| VL0080 | 5 | 0.167168171 |
| VL0081 | 5 | 0.167168171 |
| VL0082 | 5 | 0.167168171 |
| VL0083 | 5 | 0.167168171 |
| VL0084 | 5 | 0.167168171 |
| VL0085 | 5 | 0.167168171 |
| VL0086 | 5 | 0.167168171 |
| VL0087 | 5 | 0.167168171 |
| VL0088 | 5 | 0.167168171 |
| VL0089 | 5 | 0.167168171 |
| VL0090 | 4 | 0.133734537 |
| VL0091 | 4 | 0.133734537 |
| VL0092 | 4 | 0.133734537 |
| VL0093 | 4 | 0.133734537 |
| VL0094 | 4 | 0.133734537 |
| VL0095 | 4 | 0.133734537 |
| VL0096 | 4 | 0.133734537 |
| VL0097 | 4 | 0.133734537 |
| VL0098 | 4 | 0.133734537 |
| VL0099 | 4 | 0.133734537 |
| VL0100 | 4 | 0.133734537 |
| VL0101 | 4 | 0.133734537 |
| VL0102 | 4 | 0.133734537 |
| VL0103 | 4 | 0.133734537 |
| VL0104 | 4 | 0.133734537 |
| VL0105 | 4 | 0.133734537 |
| VL0106 | 4 | 0.133734537 |
| VL0107 | 4 | 0.133734537 |
| VL0108 | 3 | 0.100300903 |
| VL0109 | 3 | 0.100300903 |
| VL0110 | 3 | 0.100300903 |
| VL0111 | 3 | 0.100300903 |
| VL0112 | 3 | 0.100300903 |
| VL0113 | 3 | 0.100300903 |
| VL0114 | 3 | 0.100300903 |
| VL0115 | 3 | 0.100300903 |
| VL0116 | 3 | 0.100300903 |
| VL0117 | 3 | 0.100300903 |
| VL0118 | 3 | 0.100300903 |
| VL0119 | 3 | 0.100300903 |
| VL0120 | 3 | 0.100300903 |
| VL0121 | 3 | 0.100300903 |
| VL0122 | 3 | 0.100300903 |
| VL0123 | 3 | 0.100300903 |
| VL0124 | 3 | 0.100300903 |
| VL0125 | 3 | 0.100300903 |
| VL0126 | 3 | 0.100300903 |
| VL0127 | 3 | 0.100300903 |
| VL0128 | 3 | 0.100300903 |
| VL0129 | 3 | 0.100300903 |
| VL0130 | 3 | 0.100300903 |
| VL0131 | 3 | 0.100300903 |
| VL0132 | 3 | 0.100300903 |
| VL0133 | 3 | 0.100300903 |
| VL0134 | 3 | 0.100300903 |
| VL0135 | 3 | 0.100300903 |
| VL0136 | 3 | 0.100300903 |
| VL0137 | 3 | 0.100300903 |
| VL0138 | 3 | 0.100300903 |
| VL0139 | 3 | 0.100300903 |
| VL0140 | 3 | 0.100300903 |
| VL0141 | 3 | 0.100300903 |
| VL0142 | 3 | 0.100300903 |
| VL0143 | 3 | 0.100300903 |
| VL0144 | 3 | 0.100300903 |
| VL0145 | 2 | 0.066867268 |
| VL0146 | 2 | 0.066867268 |
| VL0147 | 2 | 0.066867268 |
| VL0148 | 2 | 0.066867268 |
| VL0149 | 2 | 0.066867268 |
| VL0150 | 2 | 0.066867268 |
| VL0151 | 2 | 0.066867268 |
| VL0152 | 2 | 0.066867268 |
| VL0153 | 2 | 0.066867268 |
| VL0154 | 2 | 0.066867268 |
| VL0155 | 2 | 0.066867268 |
| VL0156 | 2 | 0.066867268 |
| VL0157 | 2 | 0.066867268 |
| VL0158 | 2 | 0.066867268 |
| VL0159 | 2 | 0.066867268 |
| VL0160 | 2 | 0.066867268 |
| VL0161 | 2 | 0.066867268 |
| VL0162 | 2 | 0.066867268 |
| VL0163 | 2 | 0.066867268 |
| VL0164 | 2 | 0.066867268 |
| VL0165 | 2 | 0.066867268 |
| VL0166 | 2 | 0.066867268 |
| VL0167 | 2 | 0.066867268 |
| VL0168 | 2 | 0.066867268 |
| VL0169 | 2 | 0.066867268 |
| VL0170 | 2 | 0.066867268 |
| VL0171 | 2 | 0.066867268 |
| VL0172 | 2 | 0.066867268 |
| VL0173 | 2 | 0.066867268 |
| VL0174 | 2 | 0.066867268 |
| VL0175 | 2 | 0.066867268 |
| VL0176 | 2 | 0.066867268 |
| VL0177 | 2 | 0.066867268 |
| VL0178 | 2 | 0.066867268 |
| VL0179 | 2 | 0.066867268 |
| VL0180 | 2 | 0.066867268 |
| VL0181 | 2 | 0.066867268 |
| VL0182 | 2 | 0.066867268 |
| VL0183 | 2 | 0.066867268 |
| VL0184 | 2 | 0.066867268 |
| VL0185 | 2 | 0.066867268 |
| VL0186 | 2 | 0.066867268 |
| VL0187 | 2 | 0.066867268 |
| VL0188 | 2 | 0.066867268 |
| VL0189 | 2 | 0.066867268 |
| VL0190 | 2 | 0.066867268 |
| VL0191 | 2 | 0.066867268 |
| VL0192 | 2 | 0.066867268 |
| VL0193 | 2 | 0.066867268 |
| VL0194 | 2 | 0.066867268 |
| VL0195 | 2 | 0.066867268 |
| VL0196 | 2 | 0.066867268 |
| VL0197 | 2 | 0.066867268 |
| VL0198 | 2 | 0.066867268 |
| VL0199 | 2 | 0.066867268 |
| VL0200 | 2 | 0.066867268 |
| VL0201 | 2 | 0.066867268 |
| VL0202 | 2 | 0.066867268 |
| VL0203 | 2 | 0.066867268 |
| VL0204 | 2 | 0.066867268 |
| VL0205 | 2 | 0.066867268 |
| VL0206 | 2 | 0.066867268 |
| VL0207 | 2 | 0.066867268 |
| VL0208 | 2 | 0.066867268 |
| VL0209 | 2 | 0.066867268 |
| VL0210 | 2 | 0.066867268 |
| VL0211 | 2 | 0.066867268 |
| VL0212 | 2 | 0.066867268 |
| VL0213 | 2 | 0.066867268 |
| VL0214 | 2 | 0.066867268 |
| VL0215 | 2 | 0.066867268 |
| VL0216 | 2 | 0.066867268 |
| VL0217 | 2 | 0.066867268 |
| VL0218 | 2 | 0.066867268 |
| VL0219 | 2 | 0.066867268 |
| VL0220 | 2 | 0.066867268 |
| VL0221 | 2 | 0.066867268 |
| VL0222 | 2 | 0.066867268 |
| VL0223 | 2 | 0.066867268 |
| VL0224 | 2 | 0.066867268 |
| VL0225 | 2 | 0.066867268 |
| VL0226 | 2 | 0.066867268 |
| VL0227 | 2 | 0.066867268 |
| VL0228 | 2 | 0.066867268 |
| VL0229 | 2 | 0.066867268 |
| VL0230 | 2 | 0.066867268 |
| VL0231 | 2 | 0.066867268 |
| VL0232 | 2 | 0.066867268 |
| VL0233 | 2 | 0.066867268 |
| VL0234 | 2 | 0.066867268 |
| VL0235 | 2 | 0.066867268 |
| VL0236 | 2 | 0.066867268 |
| VL0237 | 2 | 0.066867268 |
| VL0238 | 2 | 0.066867268 |
| VL0239 | 2 | 0.066867268 |
| VL0240 | 2 | 0.066867268 |
| VL0241 | 2 | 0.066867268 |
| VL0242 | 2 | 0.066867268 |
| VL0243 | 2 | 0.066867268 |
| VL0244 | 1 | 0.033433634 |
| VL0245 | 1 | 0.033433634 |
| VL0246 | 1 | 0.033433634 |
| VL0247 | 1 | 0.033433634 |
| VL0248 | 1 | 0.033433634 |
| VL0249 | 1 | 0.033433634 |
| VL0250 | 1 | 0.033433634 |
| VL0251 | 1 | 0.033433634 |
| VL0252 | 1 | 0.033433634 |
| VL0253 | 1 | 0.033433634 |
| VL0254 | 1 | 0.033433634 |
| VL0255 | 1 | 0.033433634 |
| VL0256 | 1 | 0.033433634 |
| VL0257 | 1 | 0.033433634 |
| VL0258 | 1 | 0.033433634 |
| VL0259 | 1 | 0.033433634 |
| VL0260 | 1 | 0.033433634 |
| VL0261 | 1 | 0.033433634 |
| VL0262 | 1 | 0.033433634 |
| VL0263 | 1 | 0.033433634 |
| VL0264 | 1 | 0.033433634 |
| VL0265 | 1 | 0.033433634 |
| VL0266 | 1 | 0.033433634 |
| VL0267 | 1 | 0.033433634 |
| VL0268 | 1 | 0.033433634 |
| VL0269 | 1 | 0.033433634 |
| VL0270 | 1 | 0.033433634 |
| VL0271 | 1 | 0.033433634 |
| VL0272 | 1 | 0.033433634 |
| VL0273 | 1 | 0.033433634 |
| VL0274 | 1 | 0.033433634 |
| VL0275 | 1 | 0.033433634 |
| VL0276 | 1 | 0.033433634 |
| VL0277 | 1 | 0.033433634 |
| VL0278 | 1 | 0.033433634 |
| VL0279 | 1 | 0.033433634 |
| VL0280 | 1 | 0.033433634 |
| VL0281 | 1 | 0.033433634 |
| VL0282 | 1 | 0.033433634 |
| VL0283 | 1 | 0.033433634 |
| VL0284 | 1 | 0.033433634 |
| VL0285 | 1 | 0.033433634 |
| VL0286 | 1 | 0.033433634 |
| VL0287 | 1 | 0.033433634 |
| VL0288 | 1 | 0.033433634 |
| VL0289 | 1 | 0.033433634 |
| VL0290 | 1 | 0.033433634 |
| VL0291 | 1 | 0.033433634 |
| VL0292 | 1 | 0.033433634 |
| VL0293 | 1 | 0.033433634 |
| VL0294 | 1 | 0.033433634 |
| VL0295 | 1 | 0.033433634 |
| VL0296 | 1 | 0.033433634 |
| VL0297 | 1 | 0.033433634 |
| VL0298 | 1 | 0.033433634 |
| VL0299 | 1 | 0.033433634 |
| VL0300 | 1 | 0.033433634 |
| VL0301 | 1 | 0.033433634 |
| VL0302 | 1 | 0.033433634 |
| VL0303 | 1 | 0.033433634 |
| VL0304 | 1 | 0.033433634 |
| VL0305 | 1 | 0.033433634 |
| VL0306 | 1 | 0.033433634 |
| VL0307 | 1 | 0.033433634 |
| VL0308 | 1 | 0.033433634 |
| VL0309 | 1 | 0.033433634 |
| VL0310 | 1 | 0.033433634 |
| VL0311 | 1 | 0.033433634 |
| VL0312 | 1 | 0.033433634 |
| VL0313 | 1 | 0.033433634 |
| VL0314 | 1 | 0.033433634 |
| VL0315 | 1 | 0.033433634 |
| VL0316 | 1 | 0.033433634 |
| VL0317 | 1 | 0.033433634 |
| VL0318 | 1 | 0.033433634 |
| VL0319 | 1 | 0.033433634 |
| VL0320 | 1 | 0.033433634 |
| VL0321 | 1 | 0.033433634 |
| VL0322 | 1 | 0.033433634 |
| VL0323 | 1 | 0.033433634 |
| VL0324 | 1 | 0.033433634 |
| VL0325 | 1 | 0.033433634 |
| VL0326 | 1 | 0.033433634 |
| VL0327 | 1 | 0.033433634 |
| VL0328 | 1 | 0.033433634 |
| VL0329 | 1 | 0.033433634 |
| VL0330 | 1 | 0.033433634 |
| VL0331 | 1 | 0.033433634 |
| VL0332 | 1 | 0.033433634 |
| VL0333 | 1 | 0.033433634 |
| VL0334 | 1 | 0.033433634 |
| VL0335 | 1 | 0.033433634 |
| VL0336 | 1 | 0.033433634 |
| VL0337 | 1 | 0.033433634 |
| VL0338 | 1 | 0.033433634 |
| VL0339 | 1 | 0.033433634 |
| VL0340 | 1 | 0.033433634 |
| VL0341 | 1 | 0.033433634 |
| VL0342 | 1 | 0.033433634 |
| VL0343 | 1 | 0.033433634 |
| VL0344 | 1 | 0.033433634 |
| VL0345 | 1 | 0.033433634 |
| VL0346 | 1 | 0.033433634 |
| VL0347 | 1 | 0.033433634 |
| VL0348 | 1 | 0.033433634 |
| VL0349 | 1 | 0.033433634 |
| VL0350 | 1 | 0.033433634 |
| VL0351 | 1 | 0.033433634 |
| VL0352 | 1 | 0.033433634 |
| VL0353 | 1 | 0.033433634 |
| VL0354 | 1 | 0.033433634 |
| VL0355 | 1 | 0.033433634 |
| VL0356 | 1 | 0.033433634 |
| VL0357 | 1 | 0.033433634 |
| VL0358 | 1 | 0.033433634 |
| VL0359 | 1 | 0.033433634 |
| VL0360 | 1 | 0.033433634 |
| VL0361 | 1 | 0.033433634 |
| VL0362 | 1 | 0.033433634 |
| VL0363 | 1 | 0.033433634 |
| VL0364 | 1 | 0.033433634 |
| VL0365 | 1 | 0.033433634 |
| VL0366 | 1 | 0.033433634 |
| VL0367 | 1 | 0.033433634 |
| VL0368 | 1 | 0.033433634 |
| VL0369 | 1 | 0.033433634 |
| VL0370 | 1 | 0.033433634 |
| VL0371 | 1 | 0.033433634 |
| VL0372 | 1 | 0.033433634 |
| VL0373 | 1 | 0.033433634 |
| VL0374 | 1 | 0.033433634 |
| VL0375 | 1 | 0.033433634 |
| VL0376 | 1 | 0.033433634 |
| VL0377 | 1 | 0.033433634 |
| VL0378 | 1 | 0.033433634 |
| VL0379 | 1 | 0.033433634 |
| VL0380 | 1 | 0.033433634 |
| VL0381 | 1 | 0.033433634 |
| VL0382 | 1 | 0.033433634 |
| VL0383 | 1 | 0.033433634 |
| VL0384 | 1 | 0.033433634 |
| VL0385 | 1 | 0.033433634 |
| VL0386 | 1 | 0.033433634 |
| VL0387 | 1 | 0.033433634 |
| VL0388 | 1 | 0.033433634 |
| VL0389 | 1 | 0.033433634 |
| VL0390 | 1 | 0.033433634 |
| VL0391 | 1 | 0.033433634 |
| VL0392 | 1 | 0.033433634 |
| VL0393 | 1 | 0.033433634 |
| VL0394 | 1 | 0.033433634 |
| VL0395 | 1 | 0.033433634 |
| VL0396 | 1 | 0.033433634 |
| VL0397 | 1 | 0.033433634 |
| VL0398 | 1 | 0.033433634 |
| VL0399 | 1 | 0.033433634 |
| VL0400 | 1 | 0.033433634 |
| VL0401 | 1 | 0.033433634 |
| VL0402 | 1 | 0.033433634 |
| VL0403 | 1 | 0.033433634 |
| VL0404 | 1 | 0.033433634 |
| VL0405 | 1 | 0.033433634 |
| VL0406 | 1 | 0.033433634 |
| VL0407 | 1 | 0.033433634 |
| VL0408 | 1 | 0.033433634 |
| VL0409 | 1 | 0.033433634 |
| VL0410 | 1 | 0.033433634 |
| VL0411 | 1 | 0.033433634 |
| VL0412 | 1 | 0.033433634 |
| VL0413 | 1 | 0.033433634 |
| VL0414 | 1 | 0.033433634 |
| VL0415 | 1 | 0.033433634 |
| VL0416 | 1 | 0.033433634 |
| VL0417 | 1 | 0.033433634 |
| VL0418 | 1 | 0.033433634 |
| VL0419 | 1 | 0.033433634 |
| VL0420 | 1 | 0.033433634 |
| VL0421 | 1 | 0.033433634 |
| VL0422 | 1 | 0.033433634 |
| VL0423 | 1 | 0.033433634 |
| VL0424 | 1 | 0.033433634 |
| VL0425 | 1 | 0.033433634 |
| VL0426 | 1 | 0.033433634 |
| VL0427 | 1 | 0.033433634 |
| VL0428 | 1 | 0.033433634 |
| VL0429 | 1 | 0.033433634 |
| VL0430 | 1 | 0.033433634 |
| VL0431 | 1 | 0.033433634 |
| VL0432 | 1 | 0.033433634 |
| VL0433 | 1 | 0.033433634 |
| VL0434 | 1 | 0.033433634 |
| VL0435 | 1 | 0.033433634 |
| VL0436 | 1 | 0.033433634 |
| VL0437 | 1 | 0.033433634 |
| VL0438 | 1 | 0.033433634 |
| VL0439 | 1 | 0.033433634 |
| VL0440 | 1 | 0.033433634 |
| VL0441 | 1 | 0.033433634 |
| VL0442 | 1 | 0.033433634 |
| VL0443 | 1 | 0.033433634 |
| VL0444 | 1 | 0.033433634 |
| VL0445 | 1 | 0.033433634 |
| VL0446 | 1 | 0.033433634 |
| VL0447 | 1 | 0.033433634 |
| VL0448 | 1 | 0.033433634 |
| VL0449 | 1 | 0.033433634 |
| VL0450 | 1 | 0.033433634 |
| VL0451 | 1 | 0.033433634 |
| VL0452 | 1 | 0.033433634 |
| VL0453 | 1 | 0.033433634 |
| VL0454 | 1 | 0.033433634 |
| VL0455 | 1 | 0.033433634 |
| VL0456 | 1 | 0.033433634 |
| VL0457 | 1 | 0.033433634 |
| VL0458 | 1 | 0.033433634 |
| VL0459 | 1 | 0.033433634 |
| VL0460 | 1 | 0.033433634 |
| VL0461 | 1 | 0.033433634 |
| VL0462 | 1 | 0.033433634 |
| VL0463 | 1 | 0.033433634 |
| VL0464 | 1 | 0.033433634 |
| VL0465 | 1 | 0.033433634 |
| VL0466 | 1 | 0.033433634 |
| VL0467 | 1 | 0.033433634 |
| VL0468 | 1 | 0.033433634 |
| VL0469 | 1 | 0.033433634 |
| VL0470 | 1 | 0.033433634 |
| VL0471 | 1 | 0.033433634 |
| VL0472 | 1 | 0.033433634 |
| VL0473 | 1 | 0.033433634 |
| VL0474 | 1 | 0.033433634 |
| VL0475 | 1 | 0.033433634 |
| VL0476 | 1 | 0.033433634 |
| VL0477 | 1 | 0.033433634 |
| VL0478 | 1 | 0.033433634 |
| VL0479 | 1 | 0.033433634 |
| VL0480 | 1 | 0.033433634 |
| VL0481 | 1 | 0.033433634 |
| VL0482 | 1 | 0.033433634 |
| VL0483 | 1 | 0.033433634 |
| VL0484 | 1 | 0.033433634 |
| VL0485 | 1 | 0.033433634 |
| VL0486 | 1 | 0.033433634 |
| VL0487 | 1 | 0.033433634 |
| VL0488 | 1 | 0.033433634 |
| VL0489 | 1 | 0.033433634 |
| VL0490 | 1 | 0.033433634 |
| VL0491 | 1 | 0.033433634 |
| VL0492 | 1 | 0.033433634 |
| VL0493 | 1 | 0.033433634 |
| VL0494 | 1 | 0.033433634 |
| VL0495 | 1 | 0.033433634 |
| VL0496 | 1 | 0.033433634 |
| VL0497 | 1 | 0.033433634 |
| VL0498 | 1 | 0.033433634 |
| VL0499 | 1 | 0.033433634 |
| VL0500 | 1 | 0.033433634 |
| VL0501 | 1 | 0.033433634 |
| VL0502 | 1 | 0.033433634 |
| VL0503 | 1 | 0.033433634 |
| VL0504 | 1 | 0.033433634 |
| VL0505 | 1 | 0.033433634 |
| VL0506 | 1 | 0.033433634 |
| VL0507 | 1 | 0.033433634 |
| VL0508 | 1 | 0.033433634 |
| VL0509 | 1 | 0.033433634 |
| VL0510 | 1 | 0.033433634 |
| VL0511 | 1 | 0.033433634 |
| VL0512 | 1 | 0.033433634 |
| VL0513 | 1 | 0.033433634 |
| VL0514 | 1 | 0.033433634 |
| VL0515 | 1 | 0.033433634 |
| VL0516 | 1 | 0.033433634 |
| VL0517 | 1 | 0.033433634 |
| VL0518 | 1 | 0.033433634 |
| VL0519 | 1 | 0.033433634 |
| VL0520 | 1 | 0.033433634 |
| VL0521 | 1 | 0.033433634 |
| VL0522 | 1 | 0.033433634 |
| VL0523 | 1 | 0.033433634 |
| VL0524 | 1 | 0.033433634 |
| VL0525 | 1 | 0.033433634 |
| VL0526 | 1 | 0.033433634 |
| VL0527 | 1 | 0.033433634 |
| VL0528 | 1 | 0.033433634 |
| VL0529 | 1 | 0.033433634 |
| VL0530 | 1 | 0.033433634 |
| VL0531 | 1 | 0.033433634 |
| VL0532 | 1 | 0.033433634 |
| VL0533 | 1 | 0.033433634 |
| VL0534 | 1 | 0.033433634 |
| VL0535 | 1 | 0.033433634 |
| VL0536 | 1 | 0.033433634 |
| VL0537 | 1 | 0.033433634 |
| VL0538 | 1 | 0.033433634 |
| VL0539 | 1 | 0.033433634 |
| VL0540 | 1 | 0.033433634 |
| VL0541 | 1 | 0.033433634 |
| VL0542 | 1 | 0.033433634 |
| VL0543 | 1 | 0.033433634 |
| VL0544 | 1 | 0.033433634 |
| VL0545 | 1 | 0.033433634 |
| VL0546 | 1 | 0.033433634 |
| VL0547 | 1 | 0.033433634 |
| VL0548 | 1 | 0.033433634 |
| VL0549 | 1 | 0.033433634 |
| VL0550 | 1 | 0.033433634 |
| VL0551 | 1 | 0.033433634 |
| VL0552 | 1 | 0.033433634 |
| VL0553 | 1 | 0.033433634 |
| VL0554 | 1 | 0.033433634 |
| VL0555 | 1 | 0.033433634 |
| VL0556 | 1 | 0.033433634 |
| VL0557 | 1 | 0.033433634 |
| VL0558 | 1 | 0.033433634 |
| VL0559 | 1 | 0.033433634 |
| VL0560 | 1 | 0.033433634 |
| VL0561 | 1 | 0.033433634 |
| VL0562 | 1 | 0.033433634 |
| VL0563 | 1 | 0.033433634 |
| VL0564 | 1 | 0.033433634 |
| VL0565 | 1 | 0.033433634 |
| VL0566 | 1 | 0.033433634 |
| VL0567 | 1 | 0.033433634 |
| VL0568 | 1 | 0.033433634 |
| VL0569 | 1 | 0.033433634 |
| VL0570 | 1 | 0.033433634 |
| VL0571 | 1 | 0.033433634 |
| VL0572 | 1 | 0.033433634 |
| VL0573 | 1 | 0.033433634 |
| VL0574 | 1 | 0.033433634 |
| VL0575 | 1 | 0.033433634 |
| VL0576 | 1 | 0.033433634 |
| VL0577 | 1 | 0.033433634 |
| VL0578 | 1 | 0.033433634 |
| VL0579 | 1 | 0.033433634 |
| VL0580 | 1 | 0.033433634 |
| VL0581 | 1 | 0.033433634 |
| VL0582 | 1 | 0.033433634 |
| VL0583 | 1 | 0.033433634 |
| VL0584 | 1 | 0.033433634 |
| VL0585 | 1 | 0.033433634 |
| VL0586 | 1 | 0.033433634 |
| VL0587 | 1 | 0.033433634 |
| VL0588 | 1 | 0.033433634 |
| VL0589 | 1 | 0.033433634 |
| VL0590 | 1 | 0.033433634 |
| VL0591 | 1 | 0.033433634 |
| VL0592 | 1 | 0.033433634 |
| VL0593 | 1 | 0.033433634 |
| VL0594 | 1 | 0.033433634 |
| VL0595 | 1 | 0.033433634 |
| VL0596 | 1 | 0.033433634 |
| VL0597 | 1 | 0.033433634 |
| VL0598 | 1 | 0.033433634 |
| VL0599 | 1 | 0.033433634 |
| VL0600 | 1 | 0.033433634 |
| VL0601 | 1 | 0.033433634 |
| VL0602 | 1 | 0.033433634 |
| VL0603 | 1 | 0.033433634 |
| VL0604 | 1 | 0.033433634 |
| VL0605 | 1 | 0.033433634 |
| VL0606 | 1 | 0.033433634 |
| VL0607 | 1 | 0.033433634 |
| VL0608 | 1 | 0.033433634 |
| VL0609 | 1 | 0.033433634 |
| VL0610 | 1 | 0.033433634 |
| VL0611 | 1 | 0.033433634 |
| VL0612 | 1 | 0.033433634 |
| VL0613 | 1 | 0.033433634 |
| VL0614 | 1 | 0.033433634 |
| VL0615 | 1 | 0.033433634 |
| VL0616 | 1 | 0.033433634 |
| VL0617 | 1 | 0.033433634 |
| VL0618 | 1 | 0.033433634 |
| VL0619 | 1 | 0.033433634 |
| VL0620 | 1 | 0.033433634 |
| VL0621 | 1 | 0.033433634 |
| VL0622 | 1 | 0.033433634 |
| VL0623 | 1 | 0.033433634 |
| VL0624 | 1 | 0.033433634 |
| VL0625 | 1 | 0.033433634 |
| VL0626 | 1 | 0.033433634 |
| VL0627 | 1 | 0.033433634 |
| VL0628 | 1 | 0.033433634 |
| VL0629 | 1 | 0.033433634 |
| VL0630 | 1 | 0.033433634 |
| VL0631 | 1 | 0.033433634 |
| VL0632 | 1 | 0.033433634 |
| VL0633 | 1 | 0.033433634 |
| VL0634 | 1 | 0.033433634 |
| VL0635 | 1 | 0.033433634 |
| VL0636 | 1 | 0.033433634 |
| VL0637 | 1 | 0.033433634 |
| VL0638 | 1 | 0.033433634 |
| VL0639 | 1 | 0.033433634 |
| VL0640 | 1 | 0.033433634 |
| VL0641 | 1 | 0.033433634 |
| VL0642 | 1 | 0.033433634 |
| VL0643 | 1 | 0.033433634 |
| VL0644 | 1 | 0.033433634 |
| VL0645 | 1 | 0.033433634 |
| VL0646 | 1 | 0.033433634 |
| VL0647 | 1 | 0.033433634 |
| VL0648 | 1 | 0.033433634 |
| VL0649 | 1 | 0.033433634 |
| VL0650 | 1 | 0.033433634 |
| VL0651 | 1 | 0.033433634 |
| VL0652 | 1 | 0.033433634 |
| VL0653 | 1 | 0.033433634 |
| VL0654 | 1 | 0.033433634 |
| VL0655 | 1 | 0.033433634 |
| VL0656 | 1 | 0.033433634 |
| VL0657 | 1 | 0.033433634 |
| VL0658 | 1 | 0.033433634 |
| VL0659 | 1 | 0.033433634 |
| VL0660 | 1 | 0.033433634 |
| VL0661 | 1 | 0.033433634 |
| VL0662 | 1 | 0.033433634 |
| VL0663 | 1 | 0.033433634 |
| VL0664 | 1 | 0.033433634 |
| VL0665 | 1 | 0.033433634 |
| VL0666 | 1 | 0.033433634 |
| VL0667 | 1 | 0.033433634 |
| VL0668 | 1 | 0.033433634 |
| VL0669 | 1 | 0.033433634 |
| VL0670 | 1 | 0.033433634 |
| VL0671 | 1 | 0.033433634 |
| VL0672 | 1 | 0.033433634 |
| VL0673 | 1 | 0.033433634 |
| VL0674 | 1 | 0.033433634 |
| VL0675 | 1 | 0.033433634 |
| VL0676 | 1 | 0.033433634 |
| VL0677 | 1 | 0.033433634 |
| VL0678 | 1 | 0.033433634 |
| VL0679 | 1 | 0.033433634 |
| VL0680 | 1 | 0.033433634 |
| VL0681 | 1 | 0.033433634 |
| VL0682 | 1 | 0.033433634 |
| VL0683 | 1 | 0.033433634 |
| VL0684 | 1 | 0.033433634 |
| VL0685 | 1 | 0.033433634 |
| VL0686 | 1 | 0.033433634 |
| VL0687 | 1 | 0.033433634 |
| VL0688 | 1 | 0.033433634 |
| VL0689 | 1 | 0.033433634 |
| VL0690 | 1 | 0.033433634 |
| VL0691 | 1 | 0.033433634 |
| VL0692 | 1 | 0.033433634 |
| VL0693 | 1 | 0.033433634 |
| VL0694 | 1 | 0.033433634 |
| VL0695 | 1 | 0.033433634 |
| VL0696 | 1 | 0.033433634 |
| VL0697 | 1 | 0.033433634 |
| VL0698 | 1 | 0.033433634 |
| VL0699 | 1 | 0.033433634 |
| VL0700 | 1 | 0.033433634 |
| VL0701 | 1 | 0.033433634 |
| VL0702 | 1 | 0.033433634 |
| VL0703 | 1 | 0.033433634 |
| VL0704 | 1 | 0.033433634 |
| VL0705 | 1 | 0.033433634 |
| VL0706 | 1 | 0.033433634 |
| VL0707 | 1 | 0.033433634 |
| VL0708 | 1 | 0.033433634 |
| VL0709 | 1 | 0.033433634 |
| VL0710 | 1 | 0.033433634 |
| VL0711 | 1 | 0.033433634 |
| VL0712 | 1 | 0.033433634 |
| VL0713 | 1 | 0.033433634 |
| VL0714 | 1 | 0.033433634 |
| VL0715 | 1 | 0.033433634 |
| VL0716 | 1 | 0.033433634 |
| VL0717 | 1 | 0.033433634 |
| VL0718 | 1 | 0.033433634 |
| VL0719 | 1 | 0.033433634 |
| VL0720 | 1 | 0.033433634 |
| VL0721 | 1 | 0.033433634 |
| VL0722 | 1 | 0.033433634 |
| VL0723 | 1 | 0.033433634 |
| VL0724 | 1 | 0.033433634 |
| VL0725 | 1 | 0.033433634 |
| VL0726 | 1 | 0.033433634 |
| VL0727 | 1 | 0.033433634 |
| VL0728 | 1 | 0.033433634 |
| VL0729 | 1 | 0.033433634 |
| VL0730 | 1 | 0.033433634 |
| VL0731 | 1 | 0.033433634 |
| VL0732 | 1 | 0.033433634 |
| VL0733 | 1 | 0.033433634 |
| VL0734 | 1 | 0.033433634 |
| VL0735 | 1 | 0.033433634 |
| VL0736 | 1 | 0.033433634 |
| VL0737 | 1 | 0.033433634 |
| VL0738 | 1 | 0.033433634 |
| VL0739 | 1 | 0.033433634 |
| VL0740 | 1 | 0.033433634 |
| VL0741 | 1 | 0.033433634 |
| VL0742 | 1 | 0.033433634 |
| VL0743 | 1 | 0.033433634 |
| VL0744 | 1 | 0.033433634 |
| VL0745 | 1 | 0.033433634 |
| VL0746 | 1 | 0.033433634 |
| VL0747 | 1 | 0.033433634 |
| VL0748 | 1 | 0.033433634 |
| VL0749 | 1 | 0.033433634 |
| VL0750 | 1 | 0.033433634 |
| VL0751 | 1 | 0.033433634 |
| VL0752 | 1 | 0.033433634 |
| VL0753 | 1 | 0.033433634 |
| VL0754 | 1 | 0.033433634 |
| VL0755 | 1 | 0.033433634 |
| VL0756 | 1 | 0.033433634 |
| VL0757 | 1 | 0.033433634 |
| VL0758 | 1 | 0.033433634 |
| VL0759 | 1 | 0.033433634 |
| VL0760 | 1 | 0.033433634 |
| VL0761 | 1 | 0.033433634 |
| VL0762 | 1 | 0.033433634 |
| VL0763 | 1 | 0.033433634 |
| VL0764 | 1 | 0.033433634 |
| VL0765 | 1 | 0.033433634 |
| VL0766 | 1 | 0.033433634 |
| VL0767 | 1 | 0.033433634 |
| VL0768 | 1 | 0.033433634 |
| VL0769 | 1 | 0.033433634 |
| VL0770 | 1 | 0.033433634 |
| VL0771 | 1 | 0.033433634 |
| VL0772 | 1 | 0.033433634 |
| VL0773 | 1 | 0.033433634 |
| VL0774 | 1 | 0.033433634 |
| VL0775 | 1 | 0.033433634 |
| VL0776 | 1 | 0.033433634 |
| VL0777 | 1 | 0.033433634 |
| VL0778 | 1 | 0.033433634 |
| VL0779 | 1 | 0.033433634 |
| VL0780 | 1 | 0.033433634 |
| VL0781 | 1 | 0.033433634 |
| VL0782 | 1 | 0.033433634 |
| VL0783 | 1 | 0.033433634 |
| VL0784 | 1 | 0.033433634 |
| VL0785 | 1 | 0.033433634 |
| VL0786 | 1 | 0.033433634 |
| VL0787 | 1 | 0.033433634 |
| VL0788 | 1 | 0.033433634 |
| VL0789 | 1 | 0.033433634 |
| VL0790 | 1 | 0.033433634 |
| VL0791 | 1 | 0.033433634 |
| VL0792 | 1 | 0.033433634 |
| VL0793 | 1 | 0.033433634 |
| VL0794 | 1 | 0.033433634 |
| VL0795 | 1 | 0.033433634 |
| VL0796 | 1 | 0.033433634 |
| VL0797 | 1 | 0.033433634 |
| VL0798 | 1 | 0.033433634 |
| VL0799 | 1 | 0.033433634 |
| VL0800 | 1 | 0.033433634 |
| VL0801 | 1 | 0.033433634 |
| VL0802 | 1 | 0.033433634 |
| VL0803 | 1 | 0.033433634 |
| VL0804 | 1 | 0.033433634 |
| VL0805 | 1 | 0.033433634 |
| VL0806 | 1 | 0.033433634 |
| VL0807 | 1 | 0.033433634 |
| VL0808 | 1 | 0.033433634 |
| VL0809 | 1 | 0.033433634 |
| VL0810 | 1 | 0.033433634 |
| VL0811 | 1 | 0.033433634 |
| VL0812 | 1 | 0.033433634 |
| VL0813 | 1 | 0.033433634 |
| VL0814 | 1 | 0.033433634 |
| VL0815 | 1 | 0.033433634 |
| VL0816 | 1 | 0.033433634 |
| VL0817 | 1 | 0.033433634 |
| VL0818 | 1 | 0.033433634 |
| VL0819 | 1 | 0.033433634 |
| VL0820 | 1 | 0.033433634 |
| VL0821 | 1 | 0.033433634 |
| VL0822 | 1 | 0.033433634 |
| VL0823 | 1 | 0.033433634 |
| VL0824 | 1 | 0.033433634 |
| VL0825 | 1 | 0.033433634 |
| VL0826 | 1 | 0.033433634 |
| VL0827 | 1 | 0.033433634 |
| VL0828 | 1 | 0.033433634 |
| VL0829 | 1 | 0.033433634 |
| VL0830 | 1 | 0.033433634 |
| VL0831 | 1 | 0.033433634 |
| VL0832 | 1 | 0.033433634 |
| VL0833 | 1 | 0.033433634 |
| VL0834 | 1 | 0.033433634 |
| VL0835 | 1 | 0.033433634 |
| VL0836 | 1 | 0.033433634 |
| VL0837 | 1 | 0.033433634 |
| VL0838 | 1 | 0.033433634 |
| VL0839 | 1 | 0.033433634 |
| VL0840 | 1 | 0.033433634 |
| VL0841 | 1 | 0.033433634 |
| VL0842 | 1 | 0.033433634 |
| VL0843 | 1 | 0.033433634 |
| VL0844 | 1 | 0.033433634 |
| VL0845 | 1 | 0.033433634 |
| VL0846 | 1 | 0.033433634 |
| VL0847 | 1 | 0.033433634 |
| VL0848 | 1 | 0.033433634 |
| VL0849 | 1 | 0.033433634 |
| VL0850 | 1 | 0.033433634 |
| VL0851 | 1 | 0.033433634 |
| VL0852 | 1 | 0.033433634 |
| VL0853 | 1 | 0.033433634 |
| VL0854 | 1 | 0.033433634 |
| VL0855 | 1 | 0.033433634 |
| VL0856 | 1 | 0.033433634 |
| VL0857 | 1 | 0.033433634 |
| VL0858 | 1 | 0.033433634 |
| VL0859 | 1 | 0.033433634 |
| VL0860 | 1 | 0.033433634 |
| VL0861 | 1 | 0.033433634 |
| VL0862 | 1 | 0.033433634 |
| VL0863 | 1 | 0.033433634 |
| VL0864 | 1 | 0.033433634 |
| VL0865 | 1 | 0.033433634 |
| VL0866 | 1 | 0.033433634 |
| VL0867 | 1 | 0.033433634 |
| VL0868 | 1 | 0.033433634 |
| VL0869 | 1 | 0.033433634 |
| VL0870 | 1 | 0.033433634 |
| VL0871 | 1 | 0.033433634 |
| VL0872 | 1 | 0.033433634 |
| VL0873 | 1 | 0.033433634 |
| VL0874 | 1 | 0.033433634 |
| VL0875 | 1 | 0.033433634 |
| VL0876 | 1 | 0.033433634 |
| VL0877 | 1 | 0.033433634 |
| VL0878 | 1 | 0.033433634 |
| VL0879 | 1 | 0.033433634 |
| VL0880 | 1 | 0.033433634 |
| VL0881 | 1 | 0.033433634 |
| VL0882 | 1 | 0.033433634 |
| VL0883 | 1 | 0.033433634 |
| VL0884 | 1 | 0.033433634 |
| VL0885 | 1 | 0.033433634 |
| VL0886 | 1 | 0.033433634 |
| VL0887 | 1 | 0.033433634 |
| VL0888 | 1 | 0.033433634 |
| VL0889 | 1 | 0.033433634 |
| VL0890 | 1 | 0.033433634 |
| VL0891 | 1 | 0.033433634 |
| VL0892 | 1 | 0.033433634 |
| VL0893 | 1 | 0.033433634 |
| VL0894 | 1 | 0.033433634 |
| VL0895 | 1 | 0.033433634 |
| VL0896 | 1 | 0.033433634 |
| VL0897 | 1 | 0.033433634 |
| VL0898 | 1 | 0.033433634 |
| VL0899 | 1 | 0.033433634 |
| VL0900 | 1 | 0.033433634 |
| VL0901 | 1 | 0.033433634 |
| VL0902 | 1 | 0.033433634 |
| VL0903 | 1 | 0.033433634 |
| VL0904 | 1 | 0.033433634 |
| VL0905 | 1 | 0.033433634 |
| VL0906 | 1 | 0.033433634 |
| VL0907 | 1 | 0.033433634 |
| VL0908 | 1 | 0.033433634 |
| VL0909 | 1 | 0.033433634 |
| VL0910 | 1 | 0.033433634 |
| VL0911 | 1 | 0.033433634 |
| VL0912 | 1 | 0.033433634 |
| VL0913 | 1 | 0.033433634 |
| VL0914 | 1 | 0.033433634 |
| VL0915 | 1 | 0.033433634 |
| VL0916 | 1 | 0.033433634 |
| VL0917 | 1 | 0.033433634 |
| VL0918 | 1 | 0.033433634 |
| VL0919 | 1 | 0.033433634 |
| VL0920 | 1 | 0.033433634 |
| VL0921 | 1 | 0.033433634 |
| VL0922 | 1 | 0.033433634 |
| VL0923 | 1 | 0.033433634 |
| VL0924 | 1 | 0.033433634 |
| VL0925 | 1 | 0.033433634 |
| VL0926 | 1 | 0.033433634 |
| VL0927 | 1 | 0.033433634 |
| VL0928 | 1 | 0.033433634 |
| VL0929 | 1 | 0.033433634 |
| VL0930 | 1 | 0.033433634 |
| VL0931 | 1 | 0.033433634 |
| VL0932 | 1 | 0.033433634 |
| VL0933 | 1 | 0.033433634 |
| VL0934 | 1 | 0.033433634 |
| VL0935 | 1 | 0.033433634 |
| VL0936 | 1 | 0.033433634 |
| VL0937 | 1 | 0.033433634 |
| VL0938 | 1 | 0.033433634 |
| VL0939 | 1 | 0.033433634 |
| VL0940 | 1 | 0.033433634 |
| VL0941 | 1 | 0.033433634 |
| VL0942 | 1 | 0.033433634 |
| VL0943 | 1 | 0.033433634 |
| VL0944 | 1 | 0.033433634 |
| VL0945 | 1 | 0.033433634 |
| VL0946 | 1 | 0.033433634 |
| VL0947 | 1 | 0.033433634 |
| VL0948 | 1 | 0.033433634 |
| VL0949 | 1 | 0.033433634 |
| VL0950 | 1 | 0.033433634 |
| VL0951 | 1 | 0.033433634 |
| VL0952 | 1 | 0.033433634 |
| VL0953 | 1 | 0.033433634 |
| VL0954 | 1 | 0.033433634 |
| VL0955 | 1 | 0.033433634 |
| VL0956 | 1 | 0.033433634 |
| VL0957 | 1 | 0.033433634 |
| VL0958 | 1 | 0.033433634 |
| VL0959 | 1 | 0.033433634 |
| VL0960 | 1 | 0.033433634 |
| VL0961 | 1 | 0.033433634 |
| VL0962 | 1 | 0.033433634 |
| VL0963 | 1 | 0.033433634 |
| VL0964 | 1 | 0.033433634 |
| VL0965 | 1 | 0.033433634 |
| VL0966 | 1 | 0.033433634 |
| VL0967 | 1 | 0.033433634 |
| VL0968 | 1 | 0.033433634 |
| VL0969 | 1 | 0.033433634 |
| VL0970 | 1 | 0.033433634 |
| VL0971 | 1 | 0.033433634 |
| VL0972 | 1 | 0.033433634 |
| VL0973 | 1 | 0.033433634 |
| VL0974 | 1 | 0.033433634 |
| VL0975 | 1 | 0.033433634 |
| VL0976 | 1 | 0.033433634 |
| VL0977 | 1 | 0.033433634 |
| VL0978 | 1 | 0.033433634 |
| VL0979 | 1 | 0.033433634 |
| VL0980 | 1 | 0.033433634 |
| VL0981 | 1 | 0.033433634 |
| VL0982 | 1 | 0.033433634 |
| VL0983 | 1 | 0.033433634 |
| VL0984 | 1 | 0.033433634 |
| VL0985 | 1 | 0.033433634 |
| VL0986 | 1 | 0.033433634 |
| VL0987 | 1 | 0.033433634 |
| VL0988 | 1 | 0.033433634 |
| VL0989 | 1 | 0.033433634 |
| VL0990 | 1 | 0.033433634 |
| VL0991 | 1 | 0.033433634 |
| VL0992 | 1 | 0.033433634 |
| VL0993 | 1 | 0.033433634 |
| VL0994 | 1 | 0.033433634 |
| VL0995 | 1 | 0.033433634 |
| VL0996 | 1 | 0.033433634 |
| VL0997 | 1 | 0.033433634 |
| VL0998 | 1 | 0.033433634 |
| VL0999 | 1 | 0.033433634 |
| VL1000 | 1 | 0.033433634 |
| VL1001 | 1 | 0.033433634 |
| VL1002 | 1 | 0.033433634 |
| VL1003 | 1 | 0.033433634 |
| VL1004 | 1 | 0.033433634 |
| VL1005 | 1 | 0.033433634 |
| VL1006 | 1 | 0.033433634 |
| VL1007 | 1 | 0.033433634 |
| VL1008 | 1 | 0.033433634 |
| VL1009 | 1 | 0.033433634 |
| VL1010 | 1 | 0.033433634 |
| VL1011 | 1 | 0.033433634 |
| VL1012 | 1 | 0.033433634 |
| VL1013 | 1 | 0.033433634 |
| VL1014 | 1 | 0.033433634 |
| VL1015 | 1 | 0.033433634 |
| VL1016 | 1 | 0.033433634 |
| VL1017 | 1 | 0.033433634 |
| VL1018 | 1 | 0.033433634 |
| VL1019 | 1 | 0.033433634 |
| VL1020 | 1 | 0.033433634 |
| VL1021 | 1 | 0.033433634 |
| VL1022 | 1 | 0.033433634 |
| VL1023 | 1 | 0.033433634 |
| VL1024 | 1 | 0.033433634 |
| VL1025 | 1 | 0.033433634 |
| VL1026 | 1 | 0.033433634 |
| VL1027 | 1 | 0.033433634 |
| VL1028 | 1 | 0.033433634 |
| VL1029 | 1 | 0.033433634 |
| VL1030 | 1 | 0.033433634 |
| VL1031 | 1 | 0.033433634 |
| VL1032 | 1 | 0.033433634 |
| VL1033 | 1 | 0.033433634 |
| VL1034 | 1 | 0.033433634 |
| VL1035 | 1 | 0.033433634 |
| VL1036 | 1 | 0.033433634 |
| VL1037 | 1 | 0.033433634 |
| VL1038 | 1 | 0.033433634 |
| VL1039 | 1 | 0.033433634 |
| VL1040 | 1 | 0.033433634 |
| VL1041 | 1 | 0.033433634 |
| VL1042 | 1 | 0.033433634 |
| VL1043 | 1 | 0.033433634 |
| VL1044 | 1 | 0.033433634 |
| VL1045 | 1 | 0.033433634 |
| VL1046 | 1 | 0.033433634 |
| VL1047 | 1 | 0.033433634 |
| VL1048 | 1 | 0.033433634 |
| VL1049 | 1 | 0.033433634 |
| VL1050 | 1 | 0.033433634 |
| VL1051 | 1 | 0.033433634 |
| VL1052 | 1 | 0.033433634 |
| VL1053 | 1 | 0.033433634 |
| VL1054 | 1 | 0.033433634 |
| VL1055 | 1 | 0.033433634 |
| VL1056 | 1 | 0.033433634 |
| VL1057 | 1 | 0.033433634 |
| VL1058 | 1 | 0.033433634 |
| VL1059 | 1 | 0.033433634 |
| VL1060 | 1 | 0.033433634 |
| VL1061 | 1 | 0.033433634 |
| VL1062 | 1 | 0.033433634 |
| VL1063 | 1 | 0.033433634 |
| VL1064 | 1 | 0.033433634 |
| VL1065 | 1 | 0.033433634 |
| VL1066 | 1 | 0.033433634 |
| VL1067 | 1 | 0.033433634 |
| VL1068 | 1 | 0.033433634 |
| VL1069 | 1 | 0.033433634 |
| VL1070 | 1 | 0.033433634 |
| VL1071 | 1 | 0.033433634 |
| VL1072 | 1 | 0.033433634 |
| VL1073 | 1 | 0.033433634 |
| VL1074 | 1 | 0.033433634 |
| VL1075 | 1 | 0.033433634 |
| VL1076 | 1 | 0.033433634 |
| VL1077 | 1 | 0.033433634 |
| VL1078 | 1 | 0.033433634 |
| VL1079 | 1 | 0.033433634 |
| VL1080 | 1 | 0.033433634 |
| VL1081 | 1 | 0.033433634 |
| VL1082 | 1 | 0.033433634 |
| VL1083 | 1 | 0.033433634 |
| VL1084 | 1 | 0.033433634 |
| VL1085 | 1 | 0.033433634 |
| VL1086 | 1 | 0.033433634 |
| VL1087 | 1 | 0.033433634 |
| VL1088 | 1 | 0.033433634 |
| VL1089 | 1 | 0.033433634 |
| VL1090 | 1 | 0.033433634 |
| VL1091 | 1 | 0.033433634 |
| VL1092 | 1 | 0.033433634 |
| VL1093 | 1 | 0.033433634 |
| VL1094 | 1 | 0.033433634 |
| VL1095 | 1 | 0.033433634 |
| VL1096 | 1 | 0.033433634 |
| VL1097 | 1 | 0.033433634 |
| VL1098 | 1 | 0.033433634 |
| VL1099 | 1 | 0.033433634 |
| VL1100 | 1 | 0.033433634 |
| VL1101 | 1 | 0.033433634 |
| VL1102 | 1 | 0.033433634 |
| VL1103 | 1 | 0.033433634 |
| VL1104 | 1 | 0.033433634 |
| VL1105 | 1 | 0.033433634 |
| VL1106 | 1 | 0.033433634 |
| VL1107 | 1 | 0.033433634 |
| VL1108 | 1 | 0.033433634 |
| VL1109 | 1 | 0.033433634 |
| VL1110 | 1 | 0.033433634 |
| VL1111 | 1 | 0.033433634 |
| VL1112 | 1 | 0.033433634 |
| VL1113 | 1 | 0.033433634 |
| VL1114 | 1 | 0.033433634 |
| VL1115 | 1 | 0.033433634 |
| VL1116 | 1 | 0.033433634 |
| VL1117 | 1 | 0.033433634 |
| VL1118 | 1 | 0.033433634 |
| VL1119 | 1 | 0.033433634 |
| VL1120 | 1 | 0.033433634 |
| VL1121 | 1 | 0.033433634 |
| VL1122 | 1 | 0.033433634 |
| VL1123 | 1 | 0.033433634 |
| VL1124 | 1 | 0.033433634 |
| VL1125 | 1 | 0.033433634 |
| VL1126 | 1 | 0.033433634 |
| VL1127 | 1 | 0.033433634 |
| VL1128 | 1 | 0.033433634 |
| VL1129 | 1 | 0.033433634 |
| VL1130 | 1 | 0.033433634 |

**Table S27 Classification of Ty1/Copia and Ty3/Gypsy families**

| **Super family** |  | **Number** | **Percentage (%)** |
| --- | --- | --- | --- |
| Ty1/Copia | Ale | 115 | 33.14 |
|  | Angela | 49 | 14.12 |
|  | Bianca | 8 | 2.31 |
|  | Horpia2 | 14 | 4.03 |
|  | Lkya | 10 | 2.88 |
|  | Owis | 91 | 26.22 |
|  | Rare1 | 10 | 2.88 |
|  | Rare2 | 50 | 14.41 |
| Ty3/Gypsy | Bagy2 | 50 | 8.04 |
|  | Cereba | 40 | 6.43 |
|  | Dagan | 373 | 59.97 |
|  | Erika | 6 | 0.96 |
|  | GA | 4 | 0.64 |
|  | Geneva | 73 | 11.74 |
|  | Laura | 6 | 0.96 |
|  | Retrosat | 70 | 11.25 |

**Table S28 The percentage of reads derived from LTR retrotransposons measured by RNA-seq from six tissues in tung tree**

| **Sample** | **Total reads** | **Mapped reads (%)** | **LTR** | | **Gypsy** | | **Copia** | | **Other LTR RT** | |
| --- | --- | --- | --- | --- | --- | --- | --- | --- | --- | --- |
|  |  |  | **Reads** | **Percentage** | **Reads** | **Percentage** | **Reads** | **Percentage** | **Reads** | **Percentage** |
| Root | 64,936,708 | 91.67 | 41,718 | 0.0642% | 7430 | 0.0194% | 12,626 | 0.0114% | 21,662 | 0.0334% |
| Stem | 50,995,280 | 79.44 | 30,285 | 0.0594% | 3421 | 0.0203% | 10,335 | 0.0067% | 16,529 | 0.0324% |
| Leaf | 76,372,384 | 89.94 | 41,669 | 0.0546% | 4733 | 0.0254% | 19,375 | 0.0062% | 17,561 | 0.0230% |
| Female flower | 74,438,216 | 90.57 | 69,533 | 0.0934% | 5210 | 0.0237% | 17,610 | 0.0070% | 46,713 | 0.0628% |
| Male flower | 63,252,746 | 88.61 | 59,982 | 0.0948% | 5040 | 0.0300% | 18,994 | 0.0080% | 35,948 | 0.0568% |
| Seed | 789,108,408 | 95.95 | 449,631 | 0.0173% | 129,526 | 0.0164% | 136,577 | 0.0233% | 183,528 | 0.0570% |

**Table S29 Expression quantity (FPKM value) of the top 29 tung tree LTR families highly expressed in seed**

| **Gene** | **Root** | **Stem** | **Leaf** | **Female flower** | **Male flower** | **Seed** |
| --- | --- | --- | --- | --- | --- | --- |
| VL0631 | 8.092319 | 8.006303 | 6.414458 | 1.706871 | 5.312487 | 11.08873 |
| VL0883 | 2.209019 | 2.624494 | 5.973387 | 5.386334 | 5.130156 | 7.22597 |
| VL0861 | 1.883799 | 1.8369 | 2.148359 | 2.253322 | 2.129104 | 5.863583 |
| VL0496 | 0.728858 | 0.730687 | 1.933841 | 1.143417 | 0.780329 | 3.269679 |
| VL0088 | 0.58881 | 0.888904 | 0.986239 | 1.034833 | 1.173811 | 3.120569 |
| VL0552 | 0.918293 | 0.925285 | 1.686557 | 0.871957 | 1.764536 | 2.9408 |
| VL0826 | 0.254741 | 0.522089 | 0.521843 | 2.50044 | 2.9506 | 2.636805 |
| VL0150 | 1.580628 | 2.054055 | 2.050102 | 0.648338 | 0.531715 | 2.619298 |
| VL0835 | 0.918091 | 1.421359 | 6.239324 | 0 | 0.016514 | 2.447857 |
| VL0520 | 0.137844 | 22.16072 | 4.908813 | 38.10004 | 5.935157 | 2.382111 |
| VL1071 | 2.438324 | 2.098244 | 1.679527 | 1.96772 | 1.670929 | 2.354918 |
| VL0782 | 0.008761 | 0.048932 | 0 | 0.075398 | 0 | 2.219964 |
| VL0460 | 0.799925 | 0.650949 | 0.811581 | 0.624721 | 0.008957 | 2.135695 |
| VL0188 | 1.13376 | 1.125293 | 1.998828 | 1.406752 | 1.544601 | 1.630482 |
| VL0012 | 2.136787 | 1.559183 | 0.605148 | 0.170329 | 0.155143 | 1.587106 |
| VL0524 | 0.44412 | 0.362364 | 0.331538 | 0.4539 | 0.166061 | 1.452035 |
| VL0236 | 0.360006 | 0.363647 | 0.75151 | 0.541554 | 0.640357 | 1.401164 |
| VL0848 | 0.236198 | 0.384418 | 0.752909 | 0.446161 | 0.966846 | 1.375178 |
| VL0228 | 0.411259 | 0.148656 | 0.602734 | 1.032288 | 0.977943 | 1.374741 |
| VL0003 | 0.219942 | 0.176796 | 0.185389 | 0.058865 | 0.064364 | 1.362043 |
| VL1127 | 17.07058 | 10.77815 | 0.151209 | 0.235101 | 0.048466 | 1.306228 |
| VL1026 | 2.377122 | 2.809219 | 4.572779 | 79.75539 | 115.9007 | 1.237784 |
| VL0276 | 0.00115 | 0.176157 | 2.926163 | 0.337224 | 0.073071 | 1.218812 |
| VL1113 | 0.954345 | 0.741692 | 1.215123 | 0.629325 | 0.884435 | 1.190684 |
| VL0107 | 0.779902 | 1.33176 | 1.306248 | 2.333978 | 2.805971 | 1.189859 |
| VL0562 | 0.580446 | 0.407536 | 0.311459 | 0.176183 | 0.17664 | 1.169551 |
| VL0377 | 2.206839 | 2.500936 | 1.86184 | 3.710045 | 4.156882 | 1.064139 |
| VL0703 | 0.848481 | 3.126913 | 1.454458 | 4.096578 | 1.107515 | 1.014236 |
| VL0105 | 0.682184 | 1.519819 | 1.845069 | 1.104327 | 1.054452 | 1.013158 |

**Table S30 Expression quantity (FPKM value) of the top 21 tung tree LTR families highly expressed in root**

| **Gene** | **Root** | **Stem** | **Leaf** | **Female flower** | **Male flower** | **Seed** |
| --- | --- | --- | --- | --- | --- | --- |
| VL1115 | 21.33359 | 7.457783 | 6.243737 | 6.065106 | 4.342405 | 0.12242 |
| VL1127 | 17.07058 | 10.77815 | 0.151209 | 0.235101 | 0.048466 | 1.306228 |
| VL0631 | 8.092319 | 8.006303 | 6.414458 | 1.706871 | 5.312487 | 11.08873 |
| VL0454 | 6.310483 | 3.795426 | 0.513461 | 0.31047 | 0.170469 | 0.954286 |
| VL0661 | 2.74045 | 1.578605 | 2.516884 | 5.676684 | 3.820723 | 0.203869 |
| VL1071 | 2.438324 | 2.098244 | 1.679527 | 1.96772 | 1.670929 | 2.354918 |
| VL1026 | 2.377122 | 2.809219 | 4.572779 | 79.75539 | 115.9007 | 1.237784 |
| VL0883 | 2.209019 | 2.624494 | 5.973387 | 5.386334 | 5.130156 | 7.22597 |
| VL0377 | 2.206839 | 2.500936 | 1.86184 | 3.710045 | 4.156882 | 1.064139 |
| VL0012 | 2.136787 | 1.559183 | 0.605148 | 0.170329 | 0.155143 | 1.587106 |
| VL0220 | 2.072145 | 1.675573 | 0.348069 | 0.348467 | 0.218255 | 0.842588 |
| VL0861 | 1.883799 | 1.8369 | 2.148359 | 2.253322 | 2.129104 | 5.863583 |
| VL0150 | 1.580628 | 2.054055 | 2.050102 | 0.648338 | 0.531715 | 2.619298 |
| VL0551 | 1.513242 | 0.351403 | 0.023586 | 0.041536 | 0.02113 | 0.013707 |
| VL0938 | 1.503375 | 0.14105 | 0.087448 | 0.050588 | 0.0766 | 0.233974 |
| VL0656 | 1.47918 | 0.350603 | 2.154012 | 0.006411 | 0.007419 | 0.025392 |
| VL0039 | 1.195776 | 0.880303 | 0.050356 | 1.928643 | 1.986594 | 0.030409 |
| VL0188 | 1.13376 | 1.125293 | 1.998828 | 1.406752 | 1.544601 | 1.630482 |
| VL0015 | 1.082185 | 0.423298 | 0.125069 | 0.045023 | 0.076431 | 0.220365 |
| VL0922 | 1.053553 | 0.017271 | 0.118915 | 0.79605 | 0 | 0.650855 |
| VL0912 | 1.028603 | 0.59906 | 0 | 0 | 0 | 0.702665 |

**Table S31 Expression quantity (FPKM value) of the top 23 tung tree LTR families highly expressed in stem**

| **Gene** | **Root** | **Stem** | **Leaf** | **Female flower** | **Male flower** | **Seed** |
| --- | --- | --- | --- | --- | --- | --- |
| VL0520 | 0.137844 | 22.16072 | 4.908813 | 38.10004 | 5.935157 | 2.382111 |
| VL0259 | 0.912291 | 16.09971 | 5.661364 | 0.3524 | 0.150702 | 0.562424 |
| VL1127 | 17.07058 | 10.77815 | 0.151209 | 0.235101 | 0.048466 | 1.306228 |
| VL0631 | 8.092319 | 8.006303 | 6.414458 | 1.706871 | 5.312487 | 11.08873 |
| VL1115 | 21.33359 | 7.457783 | 6.243737 | 6.065106 | 4.342405 | 0.12242 |
| VL0454 | 6.310483 | 3.795426 | 0.513461 | 0.31047 | 0.170469 | 0.954286 |
| VL0703 | 0.848481 | 3.126913 | 1.454458 | 4.096578 | 1.107515 | 1.014236 |
| VL1026 | 2.377122 | 2.809219 | 4.572779 | 79.75539 | 115.9007 | 1.237784 |
| VL0883 | 2.209019 | 2.624494 | 5.973387 | 5.386334 | 5.130156 | 7.22597 |
| VL0377 | 2.206839 | 2.500936 | 1.86184 | 3.710045 | 4.156882 | 1.064139 |
| VL1071 | 2.438324 | 2.098244 | 1.679527 | 1.96772 | 1.670929 | 2.354918 |
| VL0150 | 1.580628 | 2.054055 | 2.050102 | 0.648338 | 0.531715 | 2.619298 |
| VL1028 | 0.313923 | 2.031457 | 0.985075 | 0.074385 | 0.733173 | 0.153328 |
| VL0861 | 1.883799 | 1.8369 | 2.148359 | 2.253322 | 2.129104 | 5.863583 |
| VL0220 | 2.072145 | 1.675573 | 0.348069 | 0.348467 | 0.218255 | 0.842588 |
| VL0661 | 2.74045 | 1.578605 | 2.516884 | 5.676684 | 3.820723 | 0.203869 |
| VL0012 | 2.136787 | 1.559183 | 0.605148 | 0.170329 | 0.155143 | 1.587106 |
| VL0105 | 0.682184 | 1.519819 | 1.845069 | 1.104327 | 1.054452 | 1.013158 |
| VL0321 | 0.858037 | 1.428517 | 0.4515 | 1.050623 | 1.260532 | 0.119997 |
| VL0835 | 0.918091 | 1.421359 | 6.239324 | 0 | 0.016514 | 2.447857 |
| VL0107 | 0.779902 | 1.33176 | 1.306248 | 2.333978 | 2.805971 | 1.189859 |
| VL0188 | 1.13376 | 1.125293 | 1.998828 | 1.406752 | 1.544601 | 1.630482 |
| VL0814 | 0.04675 | 1.095052 | 0 | 0 | 0.003295 | 0.01613 |

**Table S32 Expression quantity (FPKM value) of the top 28 tung tree LTR families highly expressed in leaf**

| **Gene** | **Root** | **Stem** | **Leaf** | **Female flower** | **Male flower** | **Seed** |
| --- | --- | --- | --- | --- | --- | --- |
| VL0631 | 8.092319 | 8.006303 | 6.414458 | 1.706871 | 5.312487 | 11.08873 |
| VL1115 | 21.33359 | 7.457783 | 6.243737 | 6.065106 | 4.342405 | 0.12242 |
| VL0835 | 0.918091 | 1.421359 | 6.239324 | 0 | 0.016514 | 2.447857 |
| VL0883 | 2.209019 | 2.624494 | 5.973387 | 5.386334 | 5.130156 | 7.22597 |
| VL0259 | 0.912291 | 16.09971 | 5.661364 | 0.3524 | 0.150702 | 0.562424 |
| VL0520 | 0.137844 | 22.16072 | 4.908813 | 38.10004 | 5.935157 | 2.382111 |
| VL1026 | 2.377122 | 2.809219 | 4.572779 | 79.75539 | 115.9007 | 1.237784 |
| VL0276 | 0.00115 | 0.176157 | 2.926163 | 0.337224 | 0.073071 | 1.218812 |
| VL0661 | 2.74045 | 1.578605 | 2.516884 | 5.676684 | 3.820723 | 0.203869 |
| VL0656 | 1.47918 | 0.350603 | 2.154012 | 0.006411 | 0.007419 | 0.025392 |
| VL0861 | 1.883799 | 1.8369 | 2.148359 | 2.253322 | 2.129104 | 5.863583 |
| VL0150 | 1.580628 | 2.054055 | 2.050102 | 0.648338 | 0.531715 | 2.619298 |
| VL0188 | 1.13376 | 1.125293 | 1.998828 | 1.406752 | 1.544601 | 1.630482 |
| VL0496 | 0.728858 | 0.730687 | 1.933841 | 1.143417 | 0.780329 | 3.269679 |
| VL0377 | 2.206839 | 2.500936 | 1.86184 | 3.710045 | 4.156882 | 1.064139 |
| VL0105 | 0.682184 | 1.519819 | 1.845069 | 1.104327 | 1.054452 | 1.013158 |
| VL0552 | 0.918293 | 0.925285 | 1.686557 | 0.871957 | 1.764536 | 2.9408 |
| VL0626 | 0.089444 | 0 | 1.682331 | 0.344712 | 0.907399 | 0 |
| VL1071 | 2.438324 | 2.098244 | 1.679527 | 1.96772 | 1.670929 | 2.354918 |
| VL0014 | 0.335804 | 0.435906 | 1.554937 | 1.071195 | 0.940023 | 0.961424 |
| VL0703 | 0.848481 | 3.126913 | 1.454458 | 4.096578 | 1.107515 | 1.014236 |
| VL0279 | 0.047318 | 0.282033 | 1.34891 | 0.13856 | 0 | 0.397733 |
| VL0035 | 0.60895 | 0.858776 | 1.332527 | 0.637033 | 1.119235 | 0.992614 |
| VL0107 | 0.779902 | 1.33176 | 1.306248 | 2.333978 | 2.805971 | 1.189859 |
| VL0106 | 0.351077 | 0.6345 | 1.301602 | 0.669997 | 0.501688 | 0.883033 |
| VL0857 | 0.127112 | 0.174801 | 1.243526 | 0.526918 | 0.10229 | 0.956696 |
| VL1113 | 0.954345 | 0.741692 | 1.215123 | 0.629325 | 0.884435 | 1.190684 |
| VL0920 | 0 | 0.113396 | 1.082603 | 0.026823 | 0 | 0 |

**Table S33 Expression quantity (FPKM value) of the top 24 tung tree LTR families highly expressed in female flower**

| **Gene** | **Root** | **Stem** | **Leaf** | **Female flower** | **Male flower** | **Seed** |
| --- | --- | --- | --- | --- | --- | --- |
| VL1026 | 2.377122 | 2.809219 | 4.572779 | 79.75539 | 115.9007 | 1.237784 |
| VL0520 | 0.137844 | 22.16072 | 4.908813 | 38.10004 | 5.935157 | 2.382111 |
| VL0098 | 0.258193 | 0.086624 | 0.066451 | 18.21615 | 33.78163 | 0.004591 |
| VL0584 | 0.746367 | 0.069567 | 0.114307 | 8.729215 | 14.60092 | 0.017624 |
| VL1115 | 21.33359 | 7.457783 | 6.243737 | 6.065106 | 4.342405 | 0.12242 |
| VL0661 | 2.74045 | 1.578605 | 2.516884 | 5.676684 | 3.820723 | 0.203869 |
| VL0883 | 2.209019 | 2.624494 | 5.973387 | 5.386334 | 5.130156 | 7.22597 |
| VL0221 | 0.088597 | 0.041333 | 0.403978 | 5.246941 | 2.591211 | 0.365582 |
| VL0703 | 0.848481 | 3.126913 | 1.454458 | 4.096578 | 1.107515 | 1.014236 |
| VL0377 | 2.206839 | 2.500936 | 1.86184 | 3.710045 | 4.156882 | 1.064139 |
| VL0826 | 0.254741 | 0.522089 | 0.521843 | 2.50044 | 2.9506 | 2.636805 |
| VL0107 | 0.779902 | 1.33176 | 1.306248 | 2.333978 | 2.805971 | 1.189859 |
| VL0861 | 1.883799 | 1.8369 | 2.148359 | 2.253322 | 2.129104 | 5.863583 |
| VL1071 | 2.438324 | 2.098244 | 1.679527 | 1.96772 | 1.670929 | 2.354918 |
| VL0039 | 1.195776 | 0.880303 | 0.050356 | 1.928643 | 1.986594 | 0.030409 |
| VL1088 | 0.056541 | 0.084124 | 0.302071 | 1.786303 | 2.38244 | 0.325862 |
| VL0631 | 8.092319 | 8.006303 | 6.414458 | 1.706871 | 5.312487 | 11.08873 |
| VL0188 | 1.13376 | 1.125293 | 1.998828 | 1.406752 | 1.544601 | 1.630482 |
| VL0496 | 0.728858 | 0.730687 | 1.933841 | 1.143417 | 0.780329 | 3.269679 |
| VL0105 | 0.682184 | 1.519819 | 1.845069 | 1.104327 | 1.054452 | 1.013158 |
| VL0014 | 0.335804 | 0.435906 | 1.554937 | 1.071195 | 0.940023 | 0.961424 |
| VL0321 | 0.858037 | 1.428517 | 0.4515 | 1.050623 | 1.260532 | 0.119997 |
| VL0088 | 0.58881 | 0.888904 | 0.986239 | 1.034833 | 1.173811 | 3.120569 |
| VL0228 | 0.411259 | 0.148656 | 0.602734 | 1.032288 | 0.977943 | 1.374741 |

**Table S34 Expression quantity (FPKM value) of the top 27 tung tree LTR families highly expressed in male flower**

| **Gene** | **Root** | **Stem** | **Leaf** | **Female flower** | **Male flower** | **Seed** |
| --- | --- | --- | --- | --- | --- | --- |
| VL1026 | 2.377122 | 2.809219 | 4.572779 | 79.75539 | 115.9007 | 1.237784 |
| VL0098 | 0.258193 | 0.086624 | 0.066451 | 18.21615 | 33.78163 | 0.004591 |
| VL0584 | 0.746367 | 0.069567 | 0.114307 | 8.729215 | 14.60092 | 0.017624 |
| VL0520 | 0.137844 | 22.16072 | 4.908813 | 38.10004 | 5.935157 | 2.382111 |
| VL0631 | 8.092319 | 8.006303 | 6.414458 | 1.706871 | 5.312487 | 11.08873 |
| VL0883 | 2.209019 | 2.624494 | 5.973387 | 5.386334 | 5.130156 | 7.22597 |
| VL1115 | 21.33359 | 7.457783 | 6.243737 | 6.065106 | 4.342405 | 0.12242 |
| VL0377 | 2.206839 | 2.500936 | 1.86184 | 3.710045 | 4.156882 | 1.064139 |
| VL0661 | 2.74045 | 1.578605 | 2.516884 | 5.676684 | 3.820723 | 0.203869 |
| VL0826 | 0.254741 | 0.522089 | 0.521843 | 2.50044 | 2.9506 | 2.636805 |
| VL0107 | 0.779902 | 1.33176 | 1.306248 | 2.333978 | 2.805971 | 1.189859 |
| VL0221 | 0.088597 | 0.041333 | 0.403978 | 5.246941 | 2.591211 | 0.365582 |
| VL1088 | 0.056541 | 0.084124 | 0.302071 | 1.786303 | 2.38244 | 0.325862 |
| VL0861 | 1.883799 | 1.8369 | 2.148359 | 2.253322 | 2.129104 | 5.863583 |
| VL0039 | 1.195776 | 0.880303 | 0.050356 | 1.928643 | 1.986594 | 0.030409 |
| VL0552 | 0.918293 | 0.925285 | 1.686557 | 0.871957 | 1.764536 | 2.9408 |
| VL0666 | 0.132577 | 0.164701 | 0.018932 | 0.029776 | 1.700584 | 0.00065 |
| VL1071 | 2.438324 | 2.098244 | 1.679527 | 1.96772 | 1.670929 | 2.354918 |
| VL0188 | 1.13376 | 1.125293 | 1.998828 | 1.406752 | 1.544601 | 1.630482 |
| VL0321 | 0.858037 | 1.428517 | 0.4515 | 1.050623 | 1.260532 | 0.119997 |
| VL0769 | 0.004385 | 0.003187 | 0.003317 | 0.004415 | 1.241686 | 0.000311 |
| VL0088 | 0.58881 | 0.888904 | 0.986239 | 1.034833 | 1.173811 | 3.120569 |
| VL0035 | 0.60895 | 0.858776 | 1.332527 | 0.637033 | 1.119235 | 0.992614 |
| VL0703 | 0.848481 | 3.126913 | 1.454458 | 4.096578 | 1.107515 | 1.014236 |
| VL0924 | 0.006819 | 0.012854 | 0.001768 | 0.6544 | 1.06763 | 0.000242 |
| VL0105 | 0.682184 | 1.519819 | 1.845069 | 1.104327 | 1.054452 | 1.013158 |
| VL0234 | 0.382646 | 0.671551 | 0.455598 | 0.739313 | 1.010168 | 0.750374 |
