# Supplementary material for: Tung Tree (Vernicia fordii) Genome Provides A Resource for Understanding Genome Evolution and Improved Oil Production
Source: Genomics Proteomics Bioinformatics. 2020 Mar 26;17(6):558–75. doi: 10.1016/j.gpb.2019.03.006 (PMC7212303; doi:10.1016/j.gpb.2019.03.006)
Supplement: Supplementary data 4 [file mmc4.docx]

**File S4 Identification and expression of *NBS-coding* gene families**

*NBS-coding* genes in tung tree genome were identified using HMMER V3.1 [1] search analysis to screen the predicted proteome against the raw hidden Markov model (HMM) corresponding to the Pfam NBS (NB-ARC) family domain. The TIR and LRR domains in the predicted NBS-coding amino acid sequences were screened using HMMER search analysis against the HMM model Pfam TIR and LRR domains, respectively. CC motifs were analyzed using Paircoil2 [2] with a P-score cutoff of 0.025. To map the location of *NBS* genes in tung tree genome, the chromosomal distribution of *NBS* genes were generated by Mapinspect software according to their position given in the tung tree sequence [3]. The expression abundances of *NBS* genes in roots before and after *Fusarium* wilt infection were estimated according to Pertea et al [4]. Briefly, first, the HISAT2 package was applied to extract splice site and exon information from the tung tree genome annotation file and build a HISAT2 index. Then reads of each transcriptome were aligned to the tung tree genome. Second, samtools1.5 was used to sort and convert SAM files to binary BAM files. Finally, StringTie 1.3.3 was used to assemble reads to transcripts for each sample and transcripts from all samples were merged. Gene expression abundance in each sample was generated. The infection experiment design was described by Chen and colleagues [5]. The original reads data for each transcriptome were downloaded at <https://www.ncbi.nlm.nih.gov/gds/> (SRR3374614, SRR3374616, SRR3374618, SRR3374620, SRR3374622, SRR3374624, SRR3374626, SRR3374628, SRR3374630, SRR3374632, SRR3374634, and SRR3374636).

**References**

[1] Finn RD, Clements J, Eddy SR. HMMER web server: interactive sequence similarity searching. Nucleic Acids Res 2011;39:W29−37.

[2] McDonnell AV, Jiang T, Keating AE, Berger B. Paircoil2: improved prediction of coiled coils from sequence. Bioinformatics 2006;22:356−8.

[3] Zhao Y, Zhou Y, Jiang H, Li X, Gan D, Peng X, et al. Systematic analysis of sequences and expression patterns of drought-responsive members of the HD-Zip gene family in maize. PLoS One 2011;6:e28488.

[4] Pertea M, Kim D, Pertea G, Leek JT, Salzberg SL. Transcript-level expression analysis of RNA-seq experiments with HISAT, StringTie, and Ballgown. Nat Protoc 2016;11:1650−67.

[5] Chen Y, Yin H, Gao M, Zhu H, Zhang Q, Wang Y. Comparative Transcriptomics atlases reveals different gene expression pattern related to fusarium wilt disease resistance and susceptibility in two vernicia species. Front Plant Sci 2016;7:1974.
