# Supplementary material for: Tung Tree (Vernicia fordii) Genome Provides A Resource for Understanding Genome Evolution and Improved Oil Production
Source: Genomics Proteomics Bioinformatics. 2020 Mar 26;17(6):558–75. doi: 10.1016/j.gpb.2019.03.006 (PMC7212303; doi:10.1016/j.gpb.2019.03.006)
Supplement: Supplementary data 6 [file mmc6.docx]

**File S6 Identification and phylogenetic analysis of oil biosynthesis-related gene families**

To identify important genes in different species, six representative plant genomes (*V. fordii*, *J. curcas*, *R. communis*, *A. thaliana*, *S. indicum*, *and G. max*) and predicted proteome sequences were obtained from available public databases. Among them, the *A. thaliana*, *G. max*, and *R. communis* proteome sequences were downloaded from the Phytozome12 (https://phytozome.jgi.doe.gov/pz/portal.html) database, the *J. curcas* and *S. indicum* were derived from Jatropha genome database (http://www.kazusa.or.jp/jatropha/) and sinbase (http://ocri-genomics.org/Sinbase/), respectively. The tung tree proteome sequence was obtained in this study. Proteins with a PEPC domain (PF00311) were identified by the hidden Markov model-based HMMER program [1]. After local searches were performed in the proteome datasets with the PEPC domain, the resulting sequences were manually adjusted in multiple sequence alignments to correct obvious errors. Similarly, the *OLE* and *KAS* genes were also identified based on the oleosin domain (PF) and ketoacyl-synt domain (PF) by using the HMMER program [1]. The protein sequences of SAD, FAD, MAT, HAD, FAT, PP, PDCT, DAG-CPT, DGAT, and PDAT of tung tree were used as queries for a local BLASTP search against these six plant proteome datasets with an e-value cut-off of less than 1e-5. Multiple sequence alignment was performed in MUSCLE using the default parameters [2]. ML trees were constructed using FastTree with the approximate likelihood ratio test (aLRT) method [3,4] (Figures S13−S17).

**References**

[1] Prakash A, Jeffryes M, Bateman A, Finn RD. The HMMER web server for protein sequence similarity search. Curr Protoc Bioinformatics 2017;60:3.15.1−3.15.23.

[2] Edgar RC. MUSCLE: a multiple sequence alignment method with reduced time and space complexity. BMC Bioinformatics 2004;5:113.

[3] MN P, PS D, AP A. FastTree: computing large minimum evolution trees with profiles instead of a distance matrix. Mol Biol Evol 2009;26:1641−50.

[4] Price MN, Dehal PS, Arkin AP. FastTree 2 – approximately maximum-likelihood trees for large alignments. PLoS One 2009;5:e9490.


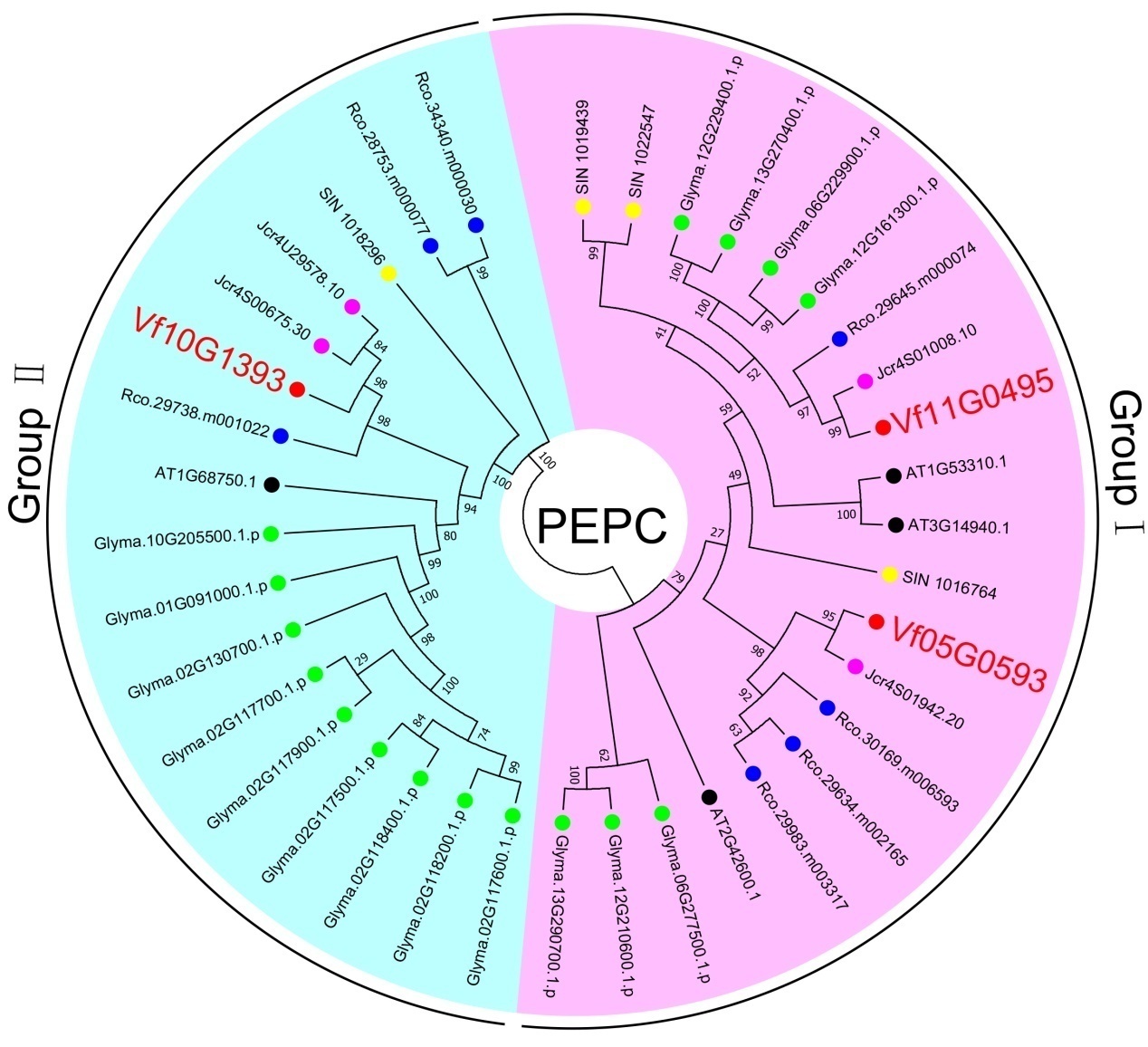


**Figure S13 Phylogenetic analysis of *PEPC* genes**

A maximum-likelihood phylogenetic tree constructed from protein sequences from *V. fordii* (red dots), *J. curcas* (pink dots), *S. indicum* (yellow dots), *R. communis* (blue dots), *G. max* (green dots), and *A. thaliana* (black dots). Different color represents different gene group generated from the tree.


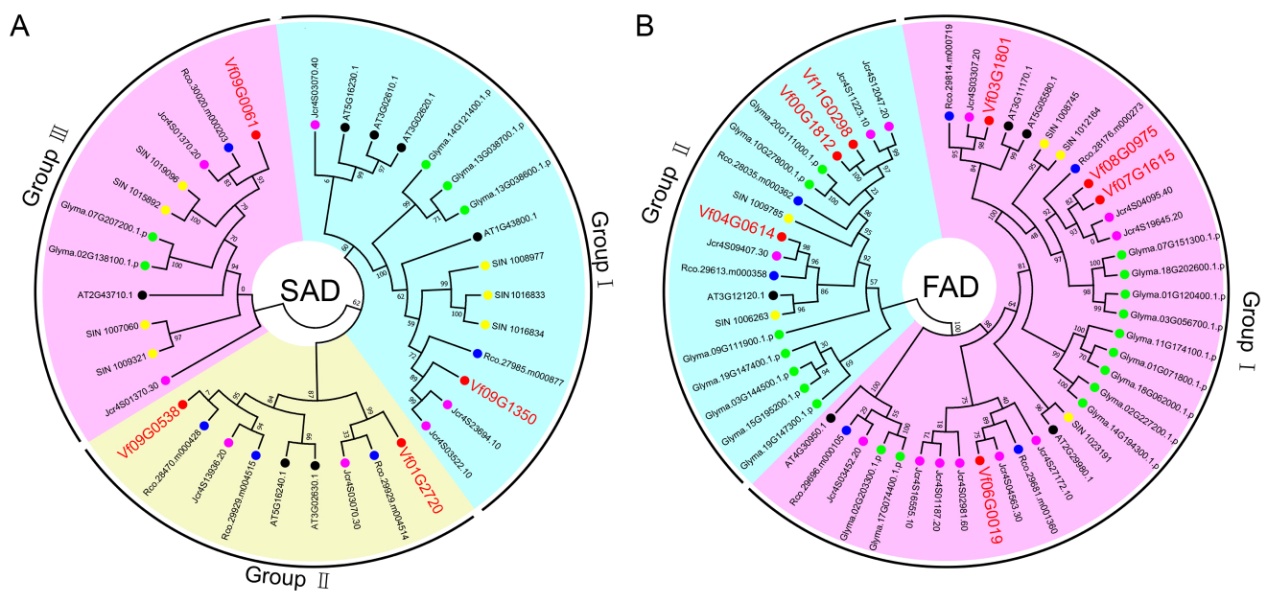


**Figure S14 Phylogenetic analysis of fatty acid desaturases**

Maximum-likelihood phylogenetic trees of SAD (**A**) and FAD (**B**) constructed from protein sequences from *V. fordii* (red dots), *J. curcas* (pink dots), *S. indicum* (yellow dots), *R. communis* (blue dots), *G. max* (green dots), and *A. thaliana* (black dots). Different color represents different gene group generated from the tree.


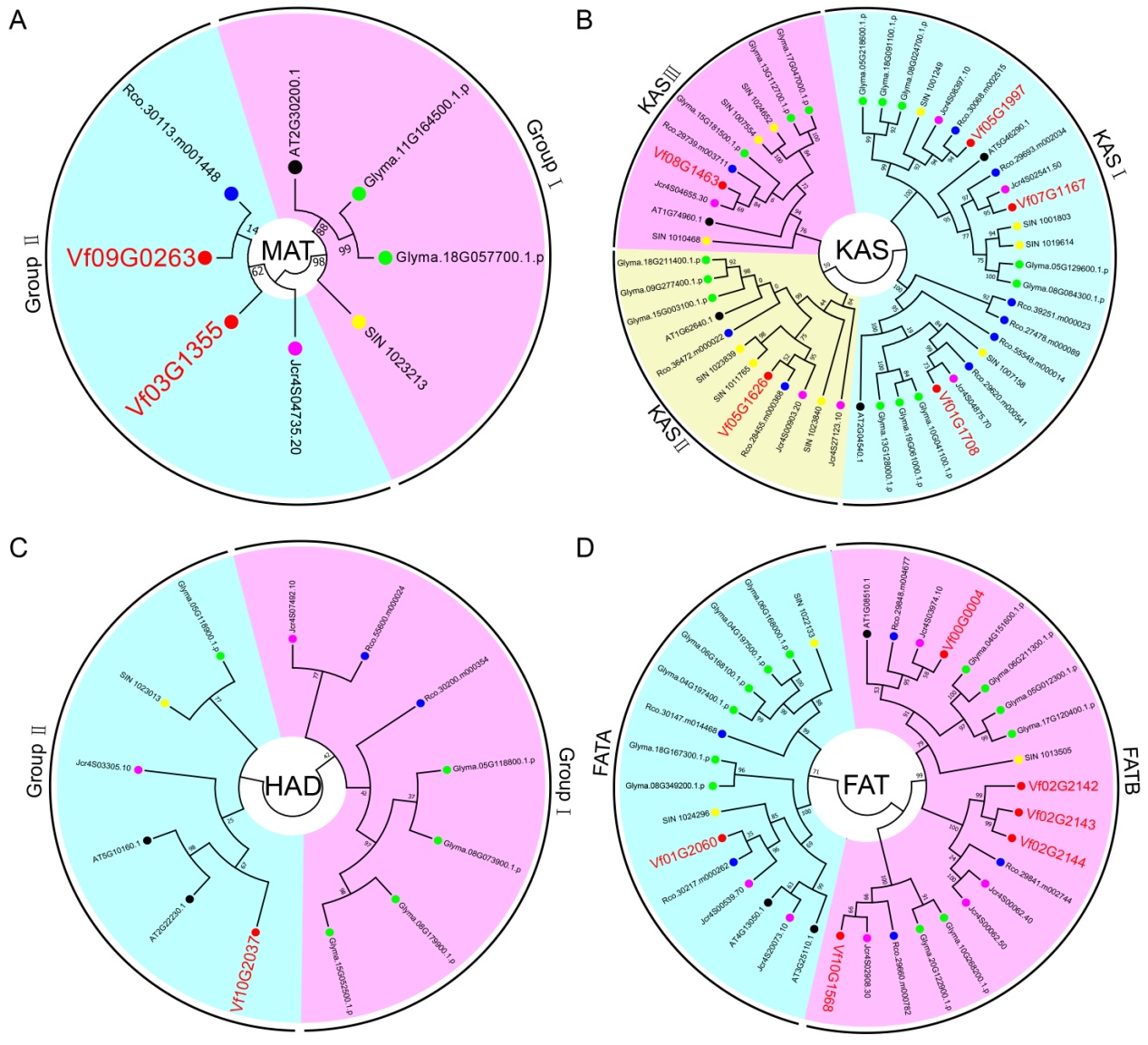


**Figure S15 Phylogenetic analysis of fatty acid synthetases**

Maximum-likelihood phylogenetic trees of MAT (**A**), KAS (**B**), HAD (**C**), and FAT (**D**) constructed from protein sequences from *V. fordii* (red dots), *J. curcas* (pink dots), *S. indicum* (yellow dots), *R. communis* (blue dots), *G. max* (green dots), and *A. thaliana* (black dots). Different color represents different gene group generated from the tree.


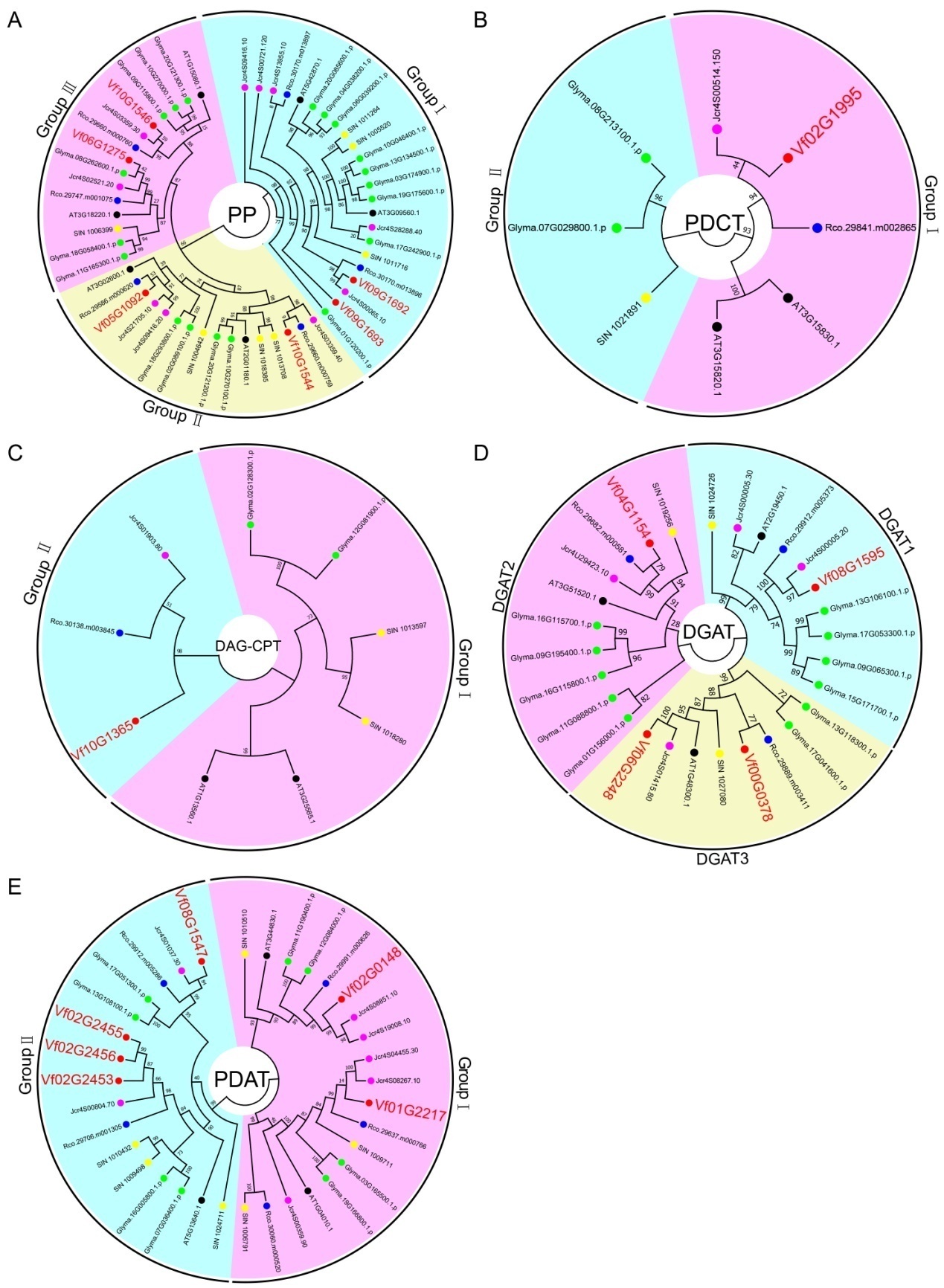


**Figure S16 Phylogenetic analysis of TAG synthesis-related genes**

Maximum-likelihood phylogenetic trees of PP (**A**), PDCT (**B**), DAG-CPT (**C**), DGAT (**D**), and PDAT (**E**) constructed from protein sequences from *V. fordii* (red dots), *J. curcas* (pink dots), *S. indicum* (yellow dots), *R. communis* (blue dots), *G. max* (green dots), and *A. thaliana* (black dots). Different color represents different gene group generated from the tree.


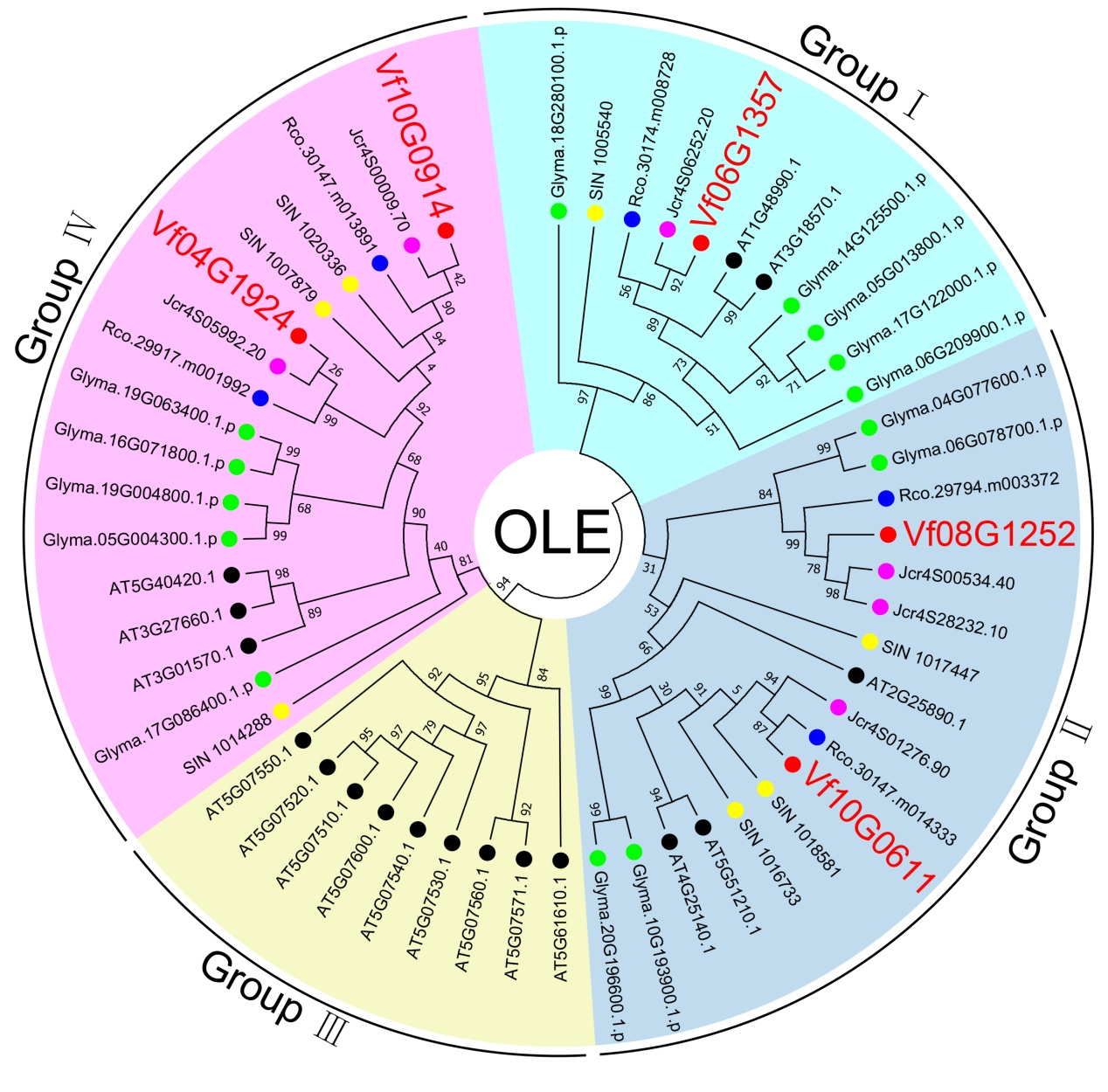


**Figure S17 Phylogenetic analysis of oleosins**

A Maximum-likelihood phylogenetic tree constructed from protein sequences from *V. fordii* (red dots), *J. curcas* (pink dots), *S. indicum* (yellow dots), *R. communis* (blue dots), *G. max* (green dots), and *A. thaliana* (black dots). Different color represents different gene group generated from the tree.
