# Supplementary material for: Tung Tree (Vernicia fordii) Genome Provides A Resource for Understanding Genome Evolution and Improved Oil Production
Source: Genomics Proteomics Bioinformatics. 2020 Mar 26;17(6):558–75. doi: 10.1016/j.gpb.2019.03.006 (PMC7212303; doi:10.1016/j.gpb.2019.03.006)
Supplement: Supplementary data 10 [file mmc10.docx]

**File S10 Data quality control and genome assembly and assessment**

The illumina raw data were filtered by FastQC [1] and NGS_QC_Toolkit [2] with the following steps: (1) Remove reads contaminated by adapters; (2) Trimming of continuous low-quality bases on both 5′ and 3′; (3) Remove reads that had Ns > 10% of the read length; (4) Remove duplicated reads caused by PCR amplification; (5) Trimming of paired-end reads if any reads with more than 50% low-quality bases. After removing low-quality reads, all the remaining data had high quality and was used for genome assembly. The whole genome assembly of tung tree was performed with a hierarchical assembly strategy due to its homozygous genome with many repetitive sequences. Firstly, Allpaths-LG was applied to assemble the clean Hiseq data, and GapCloser [3] was used to fill gaps and improve the quality of the scaffolds. Second, the paired-end or mate-pair reads were used to link the scaffolds/contigs into super-scaffold sequences using SSPACE [4]. Finally, PacBio reads were used to close gaps in scaffolds by PBJelly, an automated pipeline for gap filling and genome improvement that aligns long sequence reads to draft assembles in order to close or improve captured gaps [5]. The core eukaryotic genes (CEGs) were mapped against the genome assembly and the completeness of the CEGs were determined by Core Eukaryotic Genes Mapping Approach (CEGMA) with default parameters [6]. BUSCO analysis was also applied to validate the genome completeness [7]. In order to check the completeness of assembly, the RNA-seq reads from different tissues were mapped to the assembled genome using TopHat with default parameters [8]. Furthermore, unigenes of different transcriptomes were mapped to the assembled genome with BLAT using default parameters.

**References**

[1] Simon A. A quality control tool for high throughput seqence data. Referene Source 2010.

[2] K PR, M J. NGS QC Toolkit: a toolkit for quality control of next generation sequencing data. PLoS One 2012;7:e30619.

[3] Luo R, Liu B, Xie Y, Li Z, Huang W, Yuan J, et al. SOAPdenovo2: an empirically improved memory-efficient short-read de novo assembler. Gigascience 2012;1:18.

[4] Boetzer M, Henkel CV, Jansen HJ, Butler D, Pirovano W. Scaffolding pre-assembled contigs using SSPACE. Bioinformatics2011;29:578−9.

[5] Worley KC, English AC, Richards S, Ross-Ibarra J, Han Y, Hughes D, et al. Improving Genomes Using Long Reads and PBJelly 2. Plant and Animal Genome XXII Conference 2014.

[6] Parra G, Bradnam K, Korf I. CEGMA: a pipeline to accurately annotate core genes in eukaryotic genomes. Bioinformatics 2007;23:1061−7.

[7] Simao FA, Waterhouse RM, Ioannidis P, Kriventseva EV, Zdobnov EM. BUSCO: assessing genome assembly and annotation completeness with single-copy orthologs. Bioinformatics 2015;31:3210−2.

[8] Trapnell C, Pachter L, Salzberg SL. TopHat: discovering splice junctions with RNA-Seq. Bioinformatics 2009;25:1105−11.
