# Supplementary material for: Tung Tree (Vernicia fordii) Genome Provides A Resource for Understanding Genome Evolution and Improved Oil Production
Source: Genomics Proteomics Bioinformatics. 2020 Mar 26;17(6):558–75. doi: 10.1016/j.gpb.2019.03.006 (PMC7212303; doi:10.1016/j.gpb.2019.03.006)
Supplement: Supplementary data 13 [file mmc13.docx]

**Figure S2 Distribution of length (A) and quality (B) of PacBio raw reads**
