# Supplementary figures and images for: Tung Tree (Vernicia fordii) Genome Provides A Resource for Understanding Genome Evolution and Improved Oil Production
Source: Genomics Proteomics Bioinformatics. 2020 Mar 26;17(6):558–75. doi: 10.1016/j.gpb.2019.03.006 (PMC7212303; doi:10.1016/j.gpb.2019.03.006)

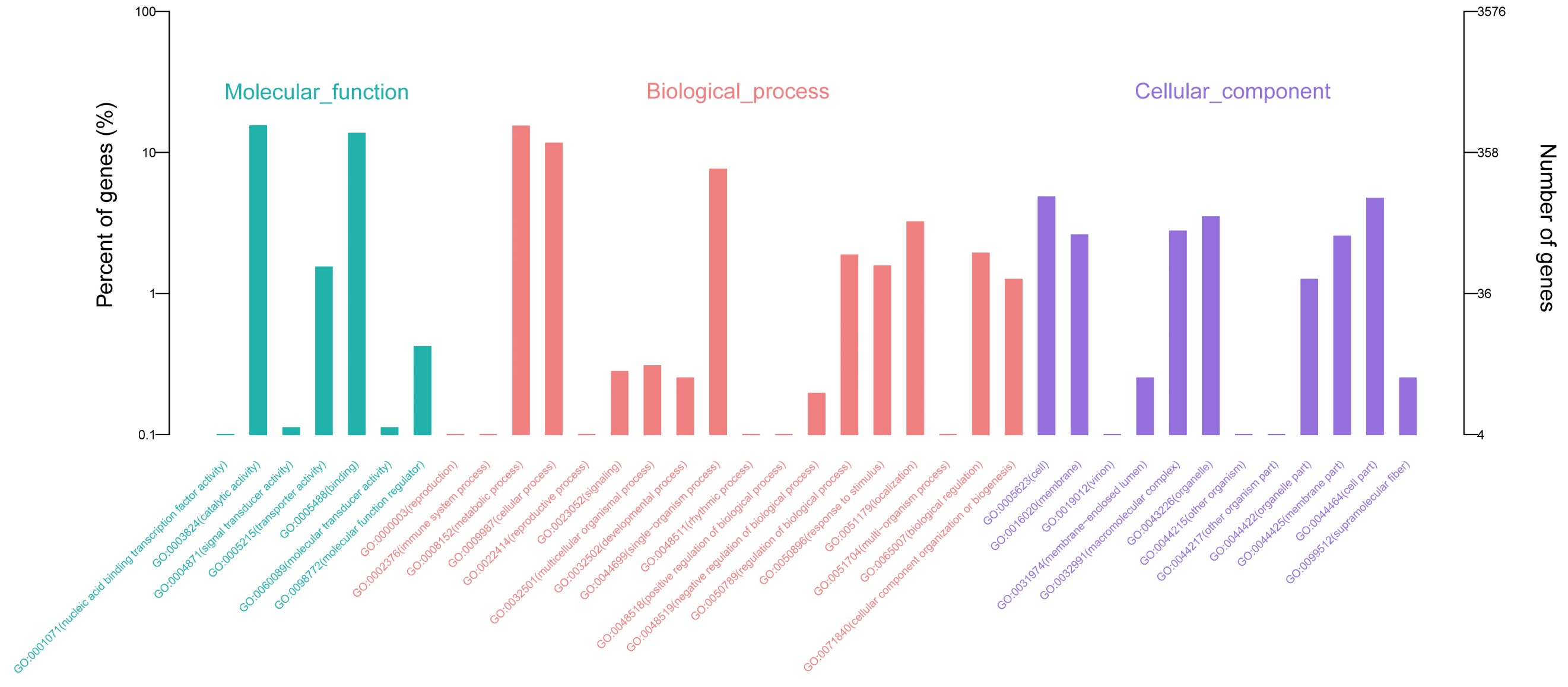


**Figure S4 GO classification of tung tree-specific gene families**

Supplement: Supplementary data 15 [file mmc15.docx]

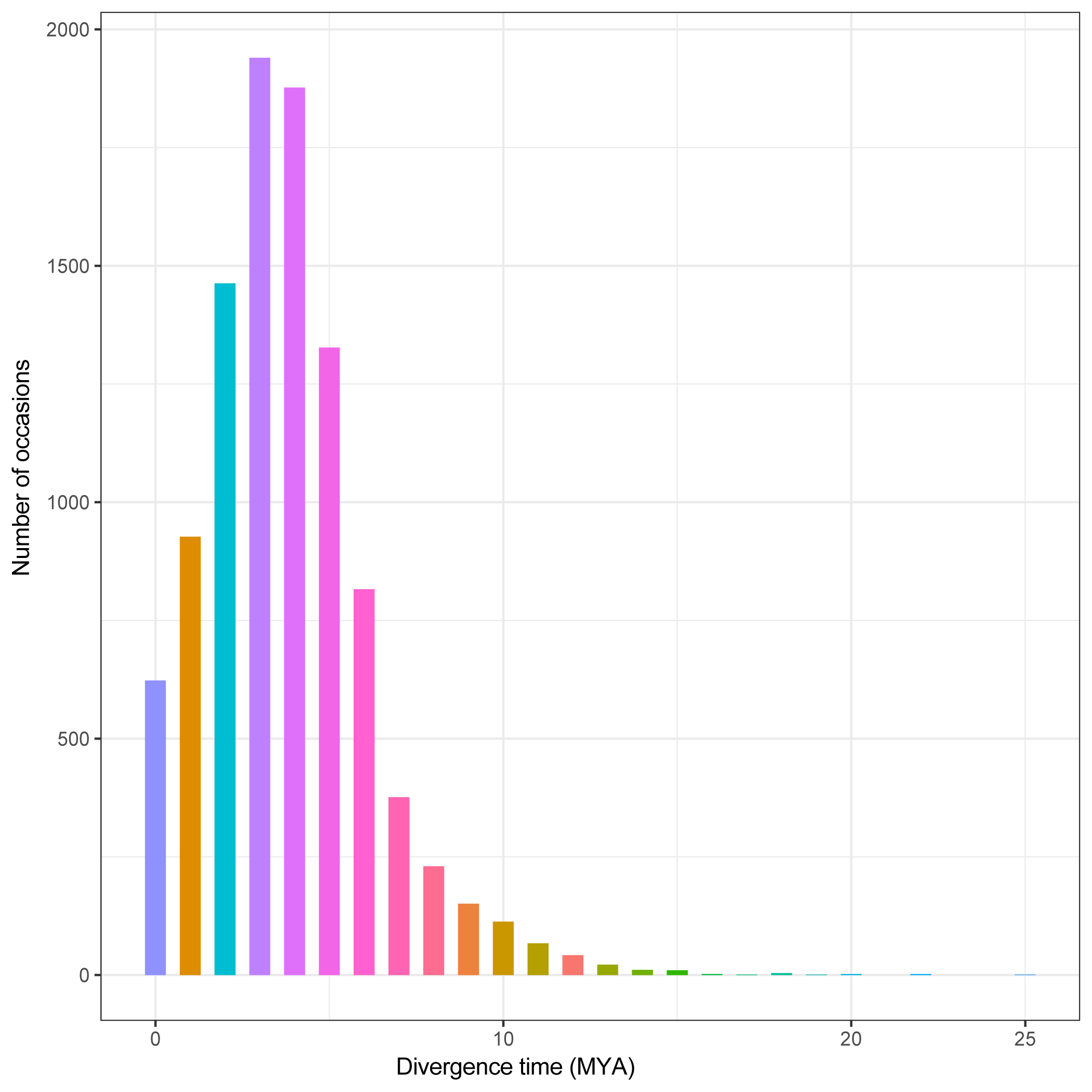


**Figure S7 Insertion time of intact LTR retrotransposons in tung tree genome**

Supplement: Supplementary data 18 [file mmc18.docx]
