# Supplementary material for: Tung Tree (Vernicia fordii) Genome Provides A Resource for Understanding Genome Evolution and Improved Oil Production
Source: Genomics Proteomics Bioinformatics. 2020 Mar 26;17(6):558–75. doi: 10.1016/j.gpb.2019.03.006 (PMC7212303; doi:10.1016/j.gpb.2019.03.006)
Supplement: Supplementary data 16 [file mmc16.docx]

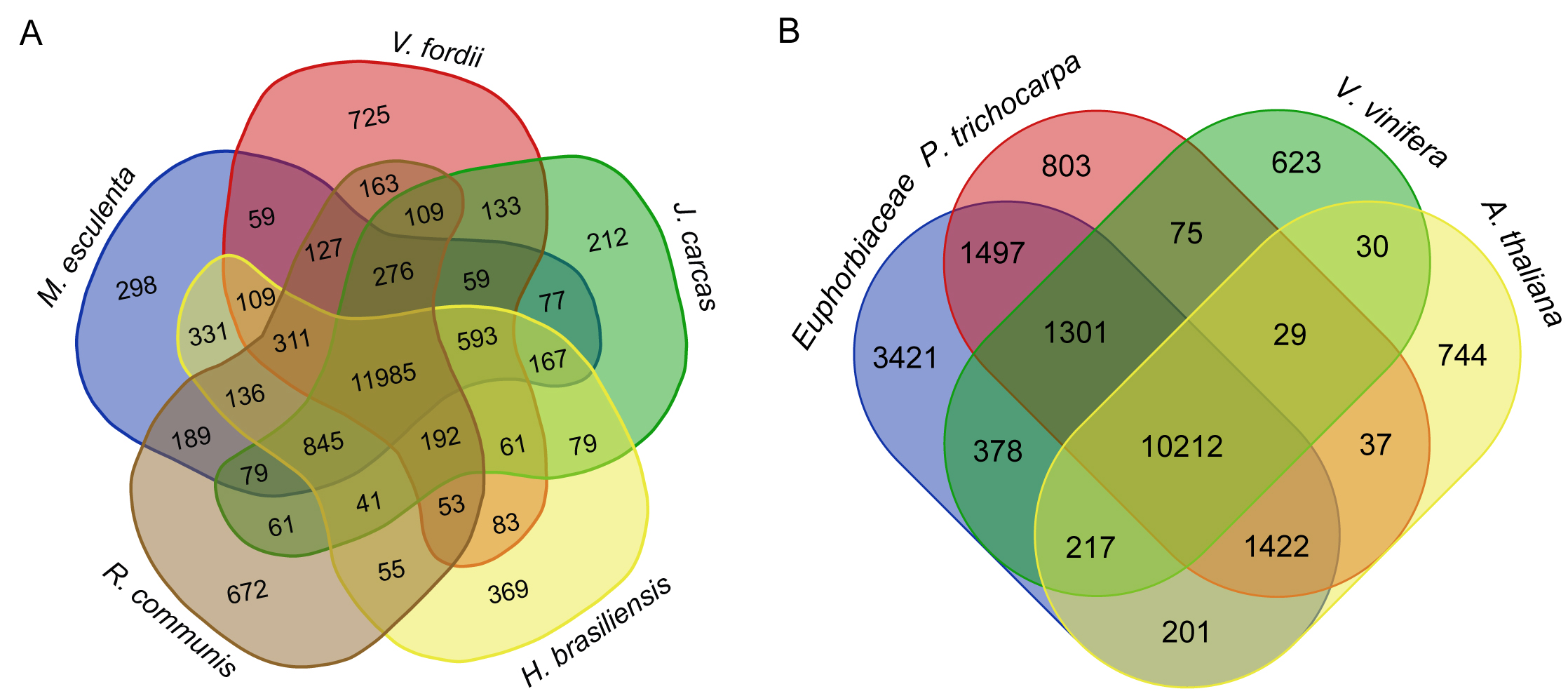


**Figure S5 Venn diagrams of cross-species comparisons of gene families**

**A.** The shared orthologues among five species in Euphorbiaceae. **B.** The shared orthologues among Euphorbiaceae, *A. thaliana*, *P. trichocarpa*, and *V. vinifera*. Each number represents a gene family number.
