# Supplementary material for: Tung Tree (Vernicia fordii) Genome Provides A Resource for Understanding Genome Evolution and Improved Oil Production
Source: Genomics Proteomics Bioinformatics. 2020 Mar 26;17(6):558–75. doi: 10.1016/j.gpb.2019.03.006 (PMC7212303; doi:10.1016/j.gpb.2019.03.006)
Supplement: Supplementary data 17 [file mmc17.docx]

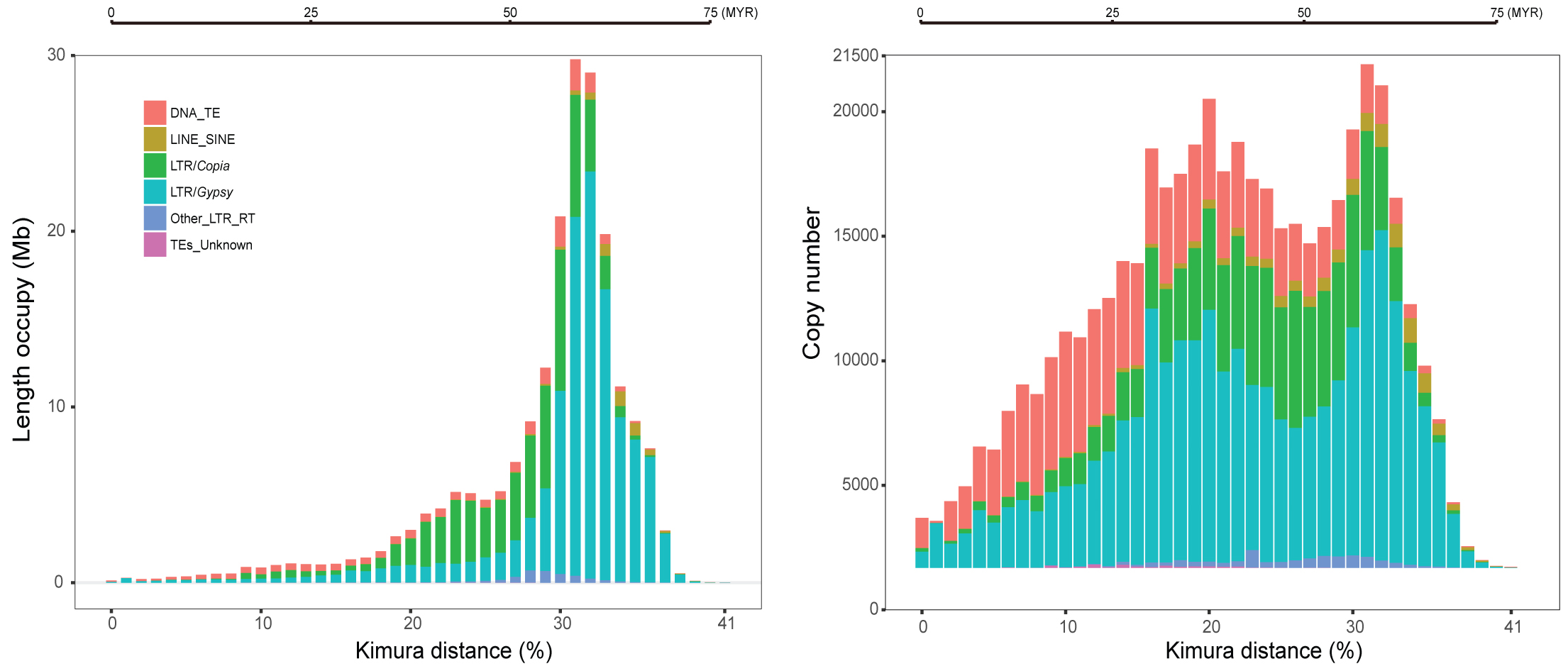


**Figure S6 Evolutionary history of TE super-families in tung tree genome**

**A.** The occupied TE lengths. **B.** copy number of TEs. Kimura distances stand for the percentages of substitutions in matching regions compared to the consensus sequences to classify TEs. TE, transposable element.
