# Supplementary material for: Tung Tree (Vernicia fordii) Genome Provides A Resource for Understanding Genome Evolution and Improved Oil Production
Source: Genomics Proteomics Bioinformatics. 2020 Mar 26;17(6):558–75. doi: 10.1016/j.gpb.2019.03.006 (PMC7212303; doi:10.1016/j.gpb.2019.03.006)
Supplement: Supplementary data 19 [file mmc19.docx]

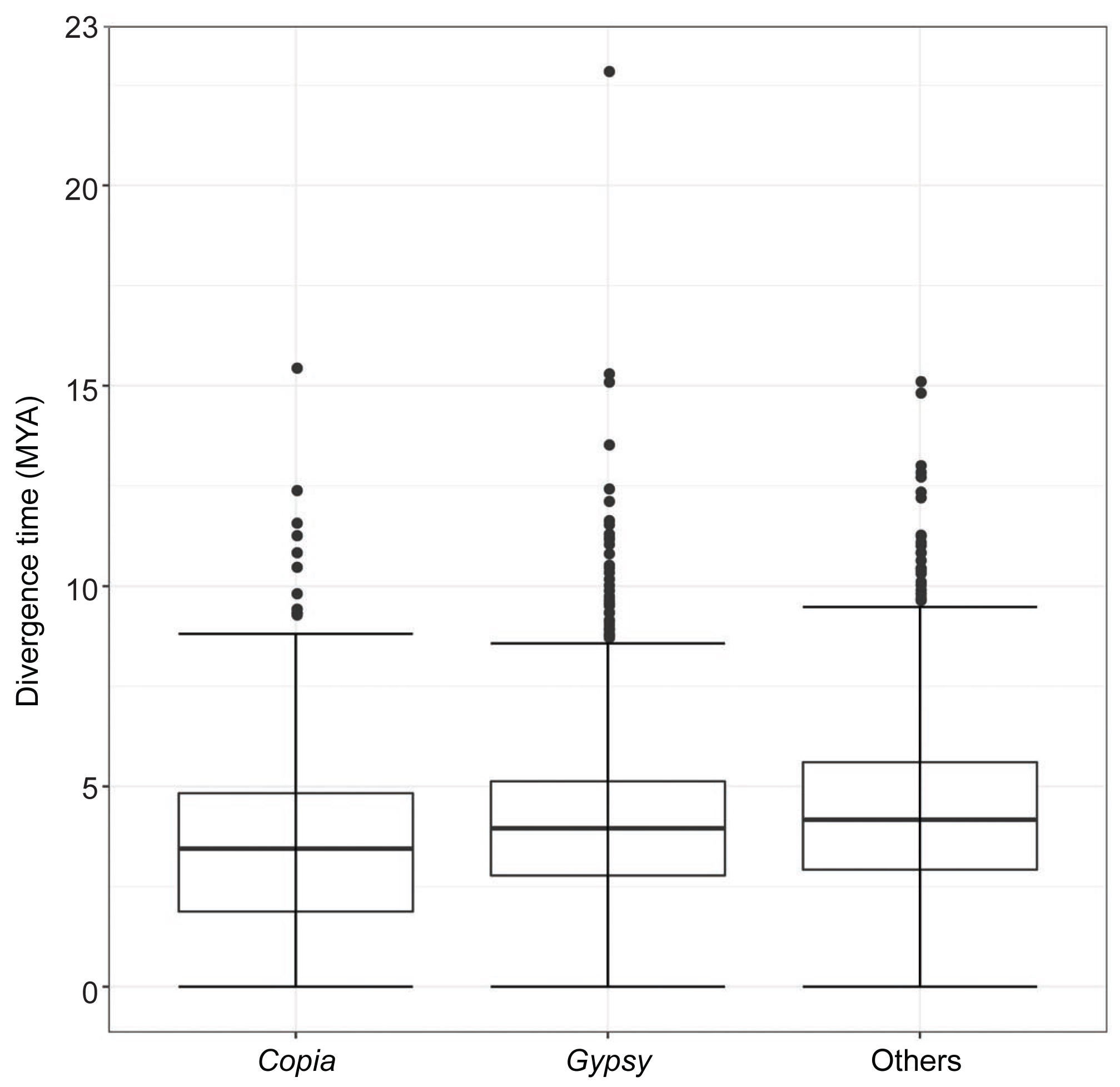


**Figure S8 Insertion time of *Ty1/Copia*, *Ty3/Gypsy*, and other LTR retrotransposon families in tung tree genome**

LTR, long terminal repeat.
