# Supplementary material for: Tung Tree (Vernicia fordii) Genome Provides A Resource for Understanding Genome Evolution and Improved Oil Production
Source: Genomics Proteomics Bioinformatics. 2020 Mar 26;17(6):558–75. doi: 10.1016/j.gpb.2019.03.006 (PMC7212303; doi:10.1016/j.gpb.2019.03.006)
Supplement: Supplementary data 20 [file mmc20.docx]

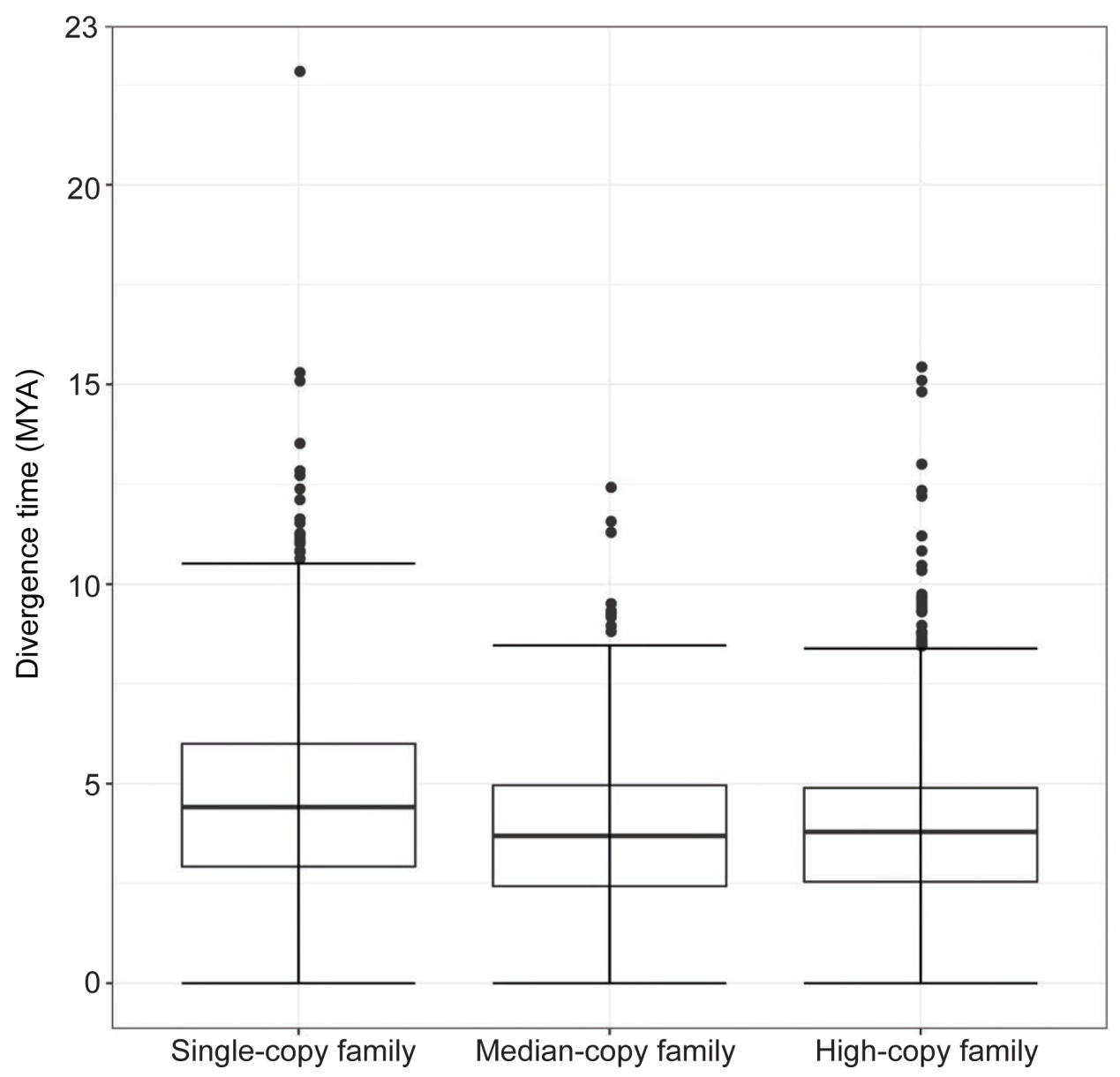


**Figure S9 Insertion time of retrotransposon families with varied copy numbers in tung tree genome**

Three types of families are shown, including single-copy families, median-copy families with 2−4 intact member and high-copy families with ≥ 5 intact member.
