# Supplementary material for: Tung Tree (Vernicia fordii) Genome Provides A Resource for Understanding Genome Evolution and Improved Oil Production
Source: Genomics Proteomics Bioinformatics. 2020 Mar 26;17(6):558–75. doi: 10.1016/j.gpb.2019.03.006 (PMC7212303; doi:10.1016/j.gpb.2019.03.006)
Supplement: Supplementary data 21 [file mmc21.docx]

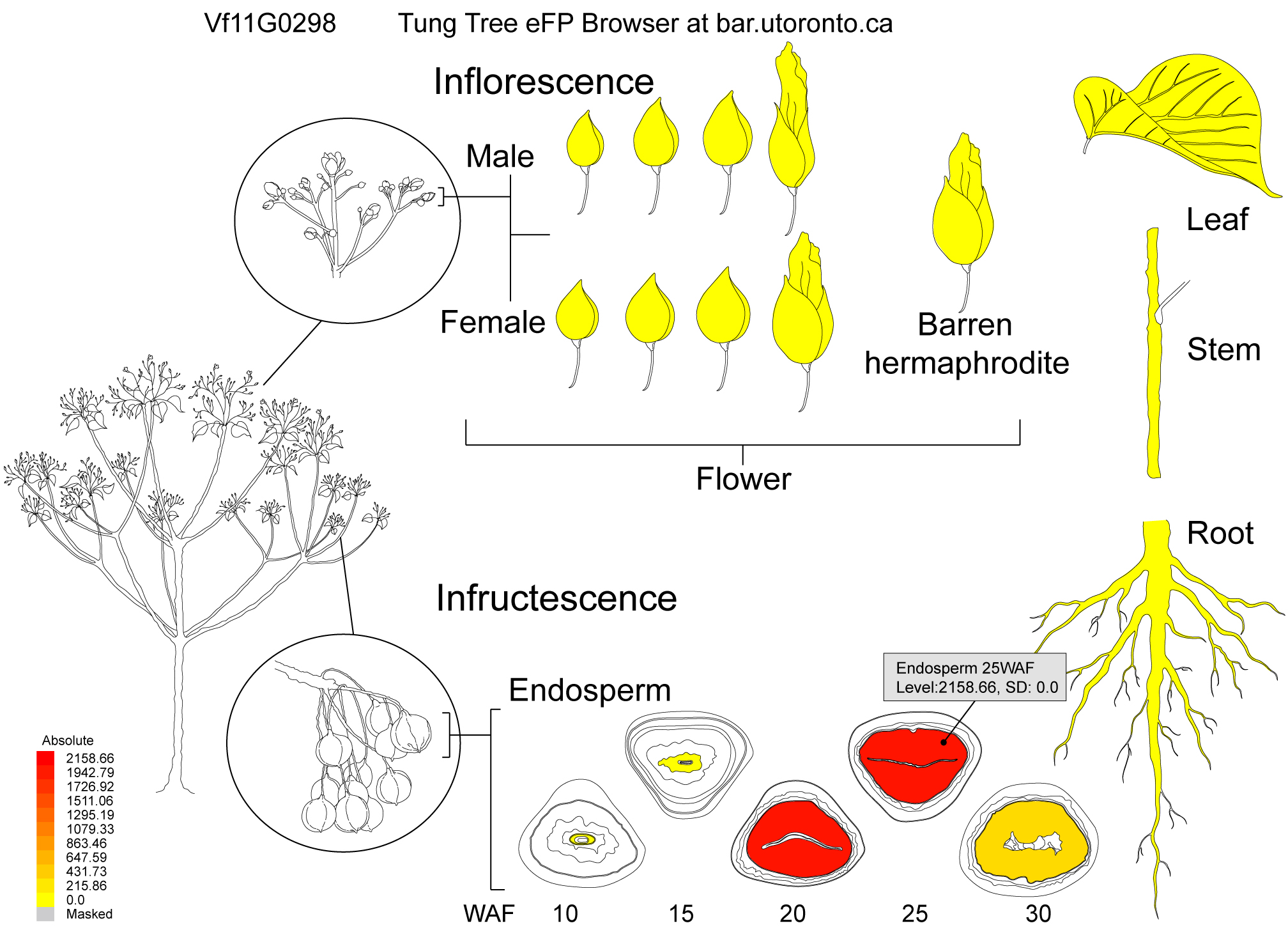


**Figure S11 eFP browser view of gene expression pattern in tung tree**

A tung tree eFP browser output image showing expression values for the *VfFADx-1* gene (Vf11G0298) is shown. Red indicates higher levels of transcript accumulation and yellow indicates a lower level of transcript accumulation. WAF, week after flower.
