# Supplementary material for: Tung Tree (Vernicia fordii) Genome Provides A Resource for Understanding Genome Evolution and Improved Oil Production
Source: Genomics Proteomics Bioinformatics. 2020 Mar 26;17(6):558–75. doi: 10.1016/j.gpb.2019.03.006 (PMC7212303; doi:10.1016/j.gpb.2019.03.006)
Supplement: Supplementary data 22 [file mmc22.docx]

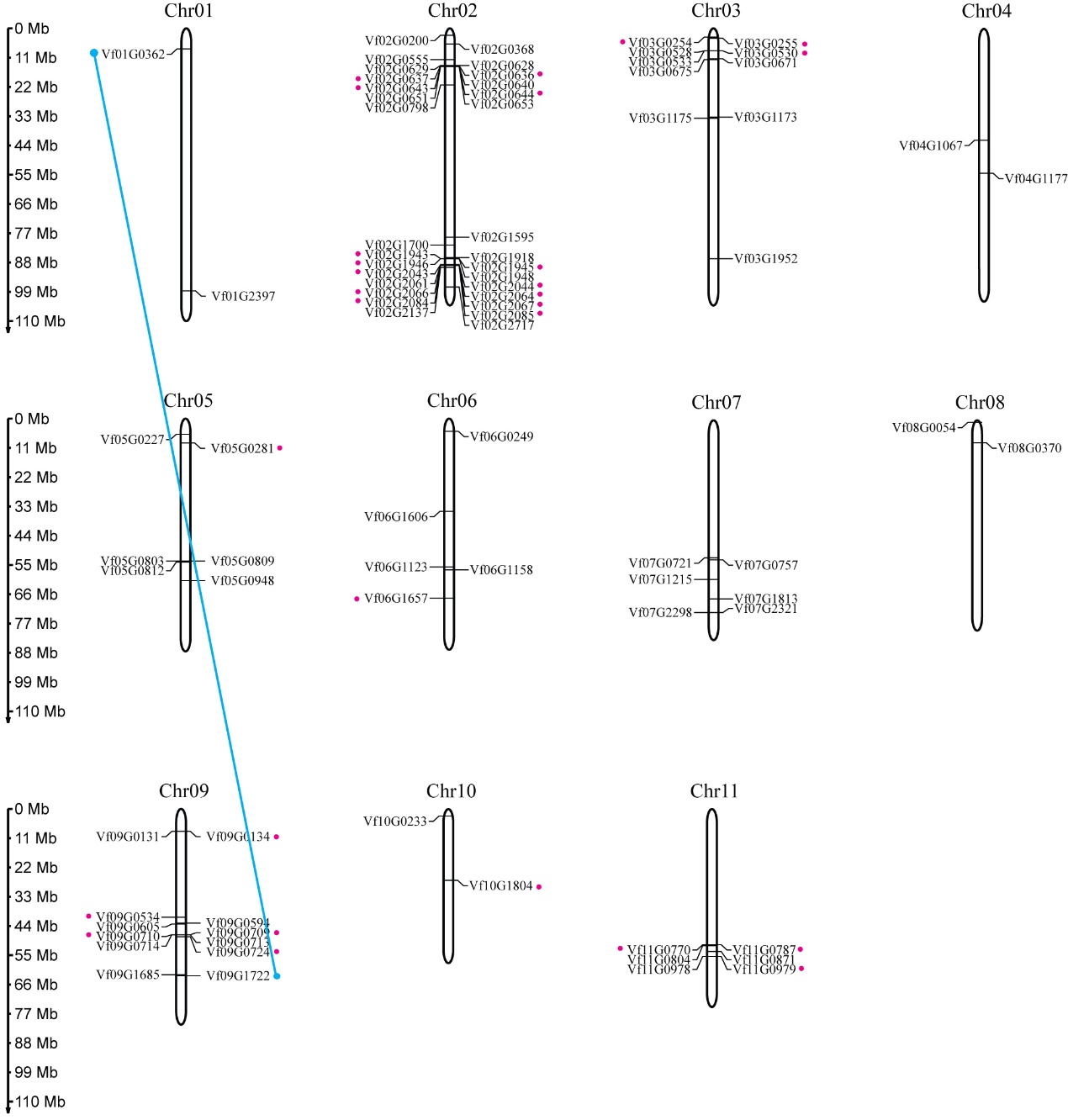


**Figure S12 Chromosomal locations and regional duplication for tung tree *NBS* genes**

The Chromosomal position of each *NBS* gene was mapped according to the tung tree genome. The chromosome number is indicated at the top of each chromosome. The scale is in mega bases (Mb). The segmental duplicated genes are indicated by blue dots and linked by blue line. The tandemly duplicated genes are indicated by pink dots. NBS, nucleotide-binding sites.
