# Supplementary material for: Tung Tree (Vernicia fordii) Genome Provides A Resource for Understanding Genome Evolution and Improved Oil Production
Source: Genomics Proteomics Bioinformatics. 2020 Mar 26;17(6):558–75. doi: 10.1016/j.gpb.2019.03.006 (PMC7212303; doi:10.1016/j.gpb.2019.03.006)
Supplement: Supplementary data 23 [file mmc23.docx]

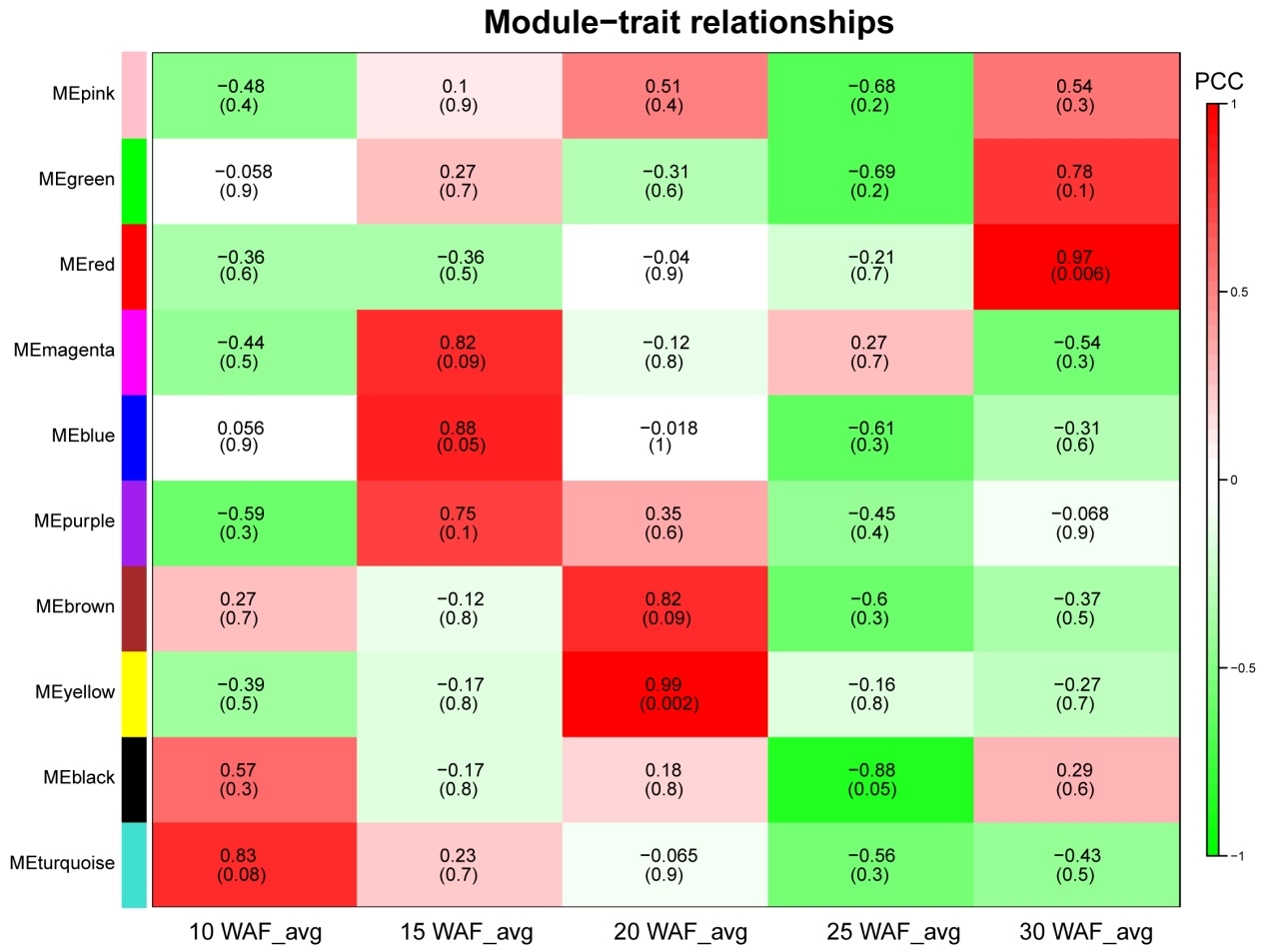


**Figure S18 Relationship between co-expression module and trait in tung tree**

The color blocks on the left side are the co-expression modules constructed from RNA-seq data of five developing seed samples. The number in each colored block represents Pearson correlation coefficient (PCC) value. The number in parentheses represents the *P* value (Student's t-test) of the correlation. ME, module; WAF, week after flower; avg, average.
