# Supplementary material for: Tung Tree (Vernicia fordii) Genome Provides A Resource for Understanding Genome Evolution and Improved Oil Production
Source: Genomics Proteomics Bioinformatics. 2020 Mar 26;17(6):558–75. doi: 10.1016/j.gpb.2019.03.006 (PMC7212303; doi:10.1016/j.gpb.2019.03.006)
Supplement: Supplementary data 24 [file mmc24.docx]

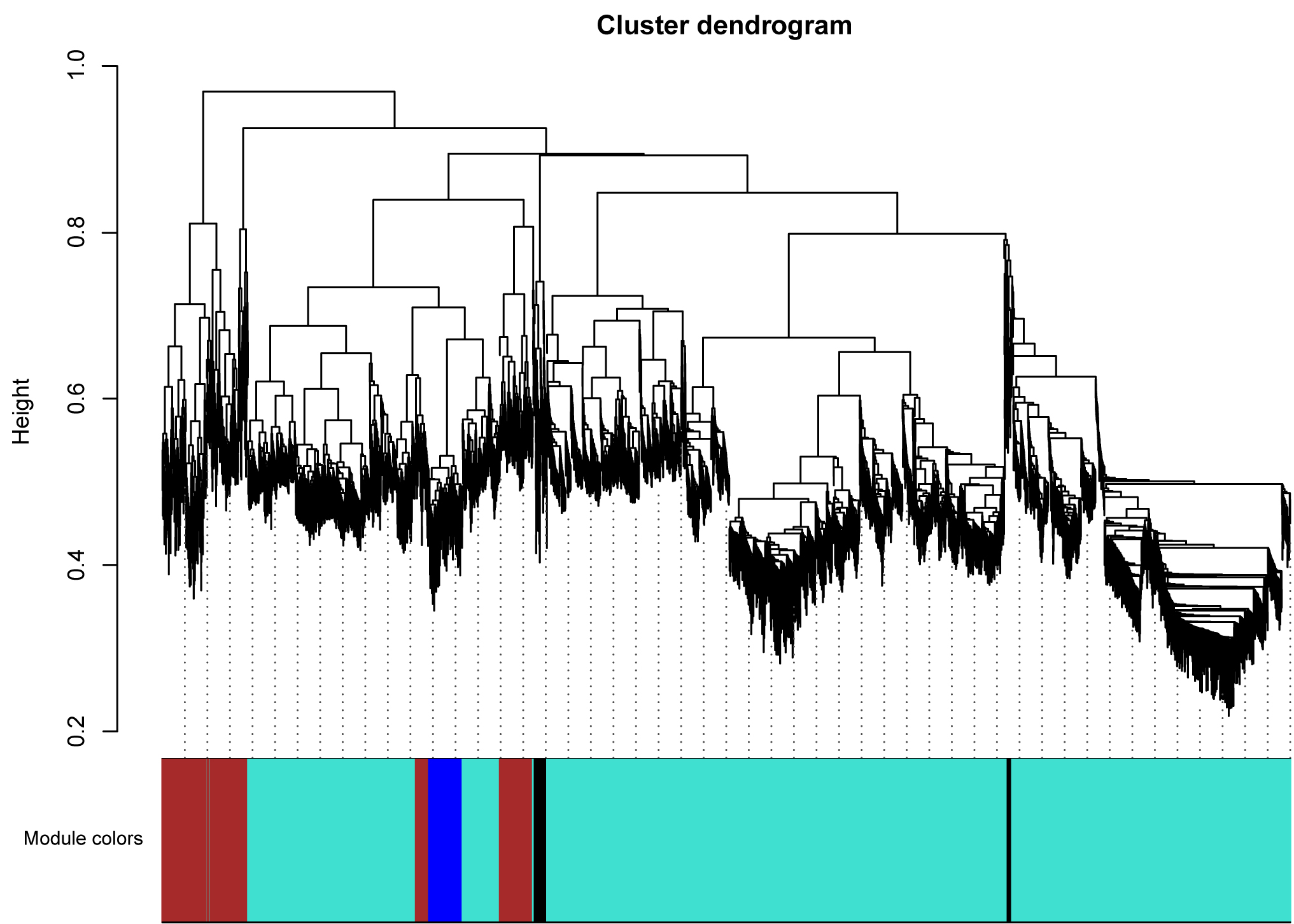


**Figure S19 Co-expression network analysis of genes in developing seeds of tung tree**

Hierarchical cluster tree showing co-expression modules identified by weighted gene co-expression network analysis of 16,732 genes whose FPKM values ≥1 in each seed sample. The major tree branches constitute 10 distinct co-expression modules labelled by different colors as indicated by a color band underneath the tree. FPKM, Fragments Per Kilobase per Million.
