# Supplementary material for: Tung Tree (Vernicia fordii) Genome Provides A Resource for Understanding Genome Evolution and Improved Oil Production
Source: Genomics Proteomics Bioinformatics. 2020 Mar 26;17(6):558–75. doi: 10.1016/j.gpb.2019.03.006 (PMC7212303; doi:10.1016/j.gpb.2019.03.006)
Supplement: Supplementary data 25 [file mmc25.docx]

**Figure S20 Yeast two-hybrid assay of transcription factors**

AD + BD, negative control plasmids; BD-Krev1 + AD-RalGDS-wt, positive control plasmids;
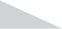
 indicates yeast concentrations from high to low; AD, Activation domain vector; BD, Binding domain vector. SC, Yeast Synthetic Medium.
