# Supplementary material for: Tung Tree (Vernicia fordii) Genome Provides A Resource for Understanding Genome Evolution and Improved Oil Production
Source: Genomics Proteomics Bioinformatics. 2020 Mar 26;17(6):558–75. doi: 10.1016/j.gpb.2019.03.006 (PMC7212303; doi:10.1016/j.gpb.2019.03.006)
Supplement: Supplementary data 27 [file mmc27.docx]

**Table S2 Statistics of the tung tree genome assembly**

| **Stat type** | **Scaffold length** | **Scaffold number** | **Contig length** | **Contig number** |
| --- | --- | --- | --- | --- |
| N50 | 803,761 | 406 | 60,554 | 5277 |
| N60 | 639,424 | 561 | 49,364 | 7219 |
| N70 | 486,231 | 761 | 38,598 | 9649 |
| N80 | 359,334 | 1028 | 28,347 | 12,844 |
| N90 | 225,380 | 1415 | 17,260 | 17,576 |
| Longest | 5,087,465 | 1 | 544,109 | 1 |
| Total | 1,118,693,778 | 4577 | 1,060,078,069 | 34,773 |
| Length > 100 bp | 1,118,693,778 | 4577 | 1,060,078,069 | 34,773 |
| Length > 2 kb | 1,116,988,597 | 3333 | 1,052,727,832 | 29,721 |
