# Supplementary material for: Tung Tree (Vernicia fordii) Genome Provides A Resource for Understanding Genome Evolution and Improved Oil Production
Source: Genomics Proteomics Bioinformatics. 2020 Mar 26;17(6):558–75. doi: 10.1016/j.gpb.2019.03.006 (PMC7212303; doi:10.1016/j.gpb.2019.03.006)
Supplement: Supplementary data 29 [file mmc29.docx]

**Table S4 Scaffold information after Hi-C assembly**

| **Scaffold number** | **Scaffold length (bp)** | **Scaffold N50 (bp)** | **Scaffold N90 (bp)** | **Scaffold max (bp)** | **Gap total length (bp)** |
| --- | --- | --- | --- | --- | --- |
| 3314 | 1,117,565,834 | 87,145,426 | 418,876 | 112,054,921 | 57,502,275 |

*Note*: Scaffold number, scaffold number with length ≥ 1 kb; Scaffold length (bp), scaffold length with length ≥ 1 kb; Scaffold N50 (bp), sequence length of scaffold N50 with length ≥1 kb; Scaffold N90 (bp), sequence length of scaffold N90 with length ≥ 1 Kb; Scaffold max (bp), sequence length of longest scaffold.
