# Supplementary material for: Tung Tree (Vernicia fordii) Genome Provides A Resource for Understanding Genome Evolution and Improved Oil Production
Source: Genomics Proteomics Bioinformatics. 2020 Mar 26;17(6):558–75. doi: 10.1016/j.gpb.2019.03.006 (PMC7212303; doi:10.1016/j.gpb.2019.03.006)
Supplement: Supplementary data 31 [file mmc31.docx]

**Table S6 Assessment for completeness of the tung tree genome by BUSCO**

| **Type** | **Number** | **Percentage (%)** |
| --- | --- | --- |
| Complete BUSCOs (C) | 1379 | 95.7 |
| Complete and single-copy BUSCOs (S) | 1338 | 92.9 |
| Complete and duplicated BUSCOs (D) | 41 | 2.8 |
| Fragmented BUSCOs (F) | 16 | 1.1 |
| Missing BUSCOs (M) | 45 | 3.2 |
| Total BUSCO groups searched | 1440 | - |
