# Supplementary material for: Tung Tree (Vernicia fordii) Genome Provides A Resource for Understanding Genome Evolution and Improved Oil Production
Source: Genomics Proteomics Bioinformatics. 2020 Mar 26;17(6):558–75. doi: 10.1016/j.gpb.2019.03.006 (PMC7212303; doi:10.1016/j.gpb.2019.03.006)
Supplement: Supplementary data 32 [file mmc32.docx]

**Table S7 Coverage of tung tree genome from unigenes of different organs**

| **Organ** | **Dataset** | **Number** | **Total length (bp)** | **Covered by assembly (%)** | **Covered > 90% in one sequence** | |  | **Covered > 50% in one sequence** | |
| --- | --- | --- | --- | --- | --- | --- | --- | --- | --- |
|  |  |  |  |  | **Number** | **Percentage (%)** |  | **Number** | **Percentage (%)** |
| Male flower | All | 65,481 | 44,918,574 | 94.97 | 59,173 | 90.36 |  | 62,058 | 94.77 |
|  | > 200 bp | 65,214 | 44,865,174 | 94.99 | 58,949 | 90.39 |  | 61,820 | 94.79 |
|  | > 500 bp | 30,053 | 33,955,900 | 98.03 | 28,216 | 93.88 |  | 29,392 | 97.8 |
|  | > 1000 bp | 13,570 | 22,216,702 | 99.12 | 12,792 | 94.26 |  | 13,405 | 98.78 |
| Female flower | All | 59,270 | 44,714,006 | 99.59 | 57,395 | 96.83 |  | 58,933 | 99.43 |
|  | > 200 bp | 59,088 | 44,677,606 | 99.59 | 57,217 | 96.83 |  | 58,754 | 99.43 |
|  | > 500 bp | 28,509 | 35,308,668 | 99.81 | 27,374 | 96.01 |  | 28,407 | 99.64 |
|  | > 1000 bp | 14,829 | 25,481,547 | 99.86 | 14,047 | 94.72 |  | 14,779 | 99.66 |
| Seed | All | 58,439 | 51,962,216 | 99.61 | 54,728 | 93.64 |  | 58,060 | 99.35 |
|  | > 200 bp | 58,246 | 51,923,616 | 99.61 | 54,539 | 93.63 |  | 57,867 | 99.34 |
|  | > 500 bp | 31,146 | 43,542,191 | 99.85 | 28,552 | 91.67 |  | 30,986 | 99.48 |
|  | > 1000 bp | 18,321 | 34,303,644 | 99.89 | 16,462 | 89.85 |  | 18,211 | 99.39 |
