# Supplementary material for: Tung Tree (Vernicia fordii) Genome Provides A Resource for Understanding Genome Evolution and Improved Oil Production
Source: Genomics Proteomics Bioinformatics. 2020 Mar 26;17(6):558–75. doi: 10.1016/j.gpb.2019.03.006 (PMC7212303; doi:10.1016/j.gpb.2019.03.006)
Supplement: Supplementary data 33 [file mmc33.docx]

**Table S8 Transcriptomic reads mapped to the tung tree genome**

| **Organ** | **All reads number** | **Mapped reads number** | **Mapping rate (%)** |
| --- | --- | --- | --- |
|  |  |  |  |
| Male flower | 51,746,368 | 45,686,156 | 88.3 |
| Female flower | 54,398,380 | 51,994,799 | 95.6 |
| Seed 1 | 52,734,436 | 49,093,204 | 93.1 |
| Seed 2 | 52,039,198 | 48,517,867 | 93.2 |
| Seed 3 | 51,886,702 | 48,538,495 | 93.5 |
