# Supplementary material for: Tung Tree (Vernicia fordii) Genome Provides A Resource for Understanding Genome Evolution and Improved Oil Production
Source: Genomics Proteomics Bioinformatics. 2020 Mar 26;17(6):558–75. doi: 10.1016/j.gpb.2019.03.006 (PMC7212303; doi:10.1016/j.gpb.2019.03.006)
Supplement: Supplementary data 34 [file mmc34.docx]

**Table S9 Comparison of gene modules between tung tree and other species**

| **Species** | **Total number of gene** | **Average trainscript length (bp)** | **Average CDS length (bp)** | **Average exons number per gene** | **Average exon length (b)** | **Average intron length (bp)** |
| --- | --- | --- | --- | --- | --- | --- |
| *V. fordii* | 28,422 | 3785.26 | 1033.92 | 4.85 | 213.11 | 714.36 |
| *A. thaliana* | 27,173 | 1876.19 | 1221.69 | 5.15 | 237 | 157.53 |
| *R. communis* | 29,957 | 2246.22 | 1019.48 | 4.26 | 239.49 | 376.66 |
| *O. sativa* | 39,044 | 2330.36 | 1064.12 | 4.12 | 258.54 | 406.38 |
| *V. vinifera* | 26,346 | 5936.60 | 1137.11 | 5.95 | 191.1 | 969.55 |
| *J. curcas* | 22,139 | 3478.87 | 1321.13 | 5.42 | 243.83 | 488.37 |
