# Supplementary material for: Tung Tree (Vernicia fordii) Genome Provides A Resource for Understanding Genome Evolution and Improved Oil Production
Source: Genomics Proteomics Bioinformatics. 2020 Mar 26;17(6):558–75. doi: 10.1016/j.gpb.2019.03.006 (PMC7212303; doi:10.1016/j.gpb.2019.03.006)
Supplement: Supplementary data 35 [file mmc35.docx]

**Table S10 GC content across the tung tree genome**

|  | **Base** | **A** | **T** | **G** | **C** | **N** | **GC** | **Total bases** |
| --- | --- | --- | --- | --- | --- | --- | --- | --- |
| Whole genome | Number (bp) | 351,420,780 | 351,480,608 | 178,680,351 | 178,496,330 | 58,615,709 | 357,176,681 | 1,118,693,778 |
|  | Percentage of genome (%) | 31.41 | 31.42 | 15.97 | 15.96 | 5.24 | 31.93 |  |
| Coding sequences | Number (bp) | 8,495,424 | 8,493,364 | 6,146,790 | 6,112,416 | 62 | 12,259,206 | 29,248,056 |
|  | Percentage of genome (%) | 29.05 | 29.04 | 21.02 | 20.9 | 0 | 41.91 |  |
| Intron regions | Number (bp) | 26,394,030 | 26,406,844 | 12,126,220 | 12,131,617 | 797,965 | 24,257,837 | 77,856,676 |
|  | Percentage of genome (%) | 33.9 | 33.92 | 15.58 | 15.58 | 1.02 | 31.16 |  |
