# Supplementary material for: Tung Tree (Vernicia fordii) Genome Provides A Resource for Understanding Genome Evolution and Improved Oil Production
Source: Genomics Proteomics Bioinformatics. 2020 Mar 26;17(6):558–75. doi: 10.1016/j.gpb.2019.03.006 (PMC7212303; doi:10.1016/j.gpb.2019.03.006)
Supplement: Supplementary data 36 [file mmc36.docx]

**Table S11 Assessment for completeness of predicted tung tree genes by BUSCO**

| **Type** | **Number** | **Percentage (%)** |
| --- | --- | --- |
| Complete BUSCOs (C) | 1290 | 89.6 |
| Complete and single-copy BUSCOs (S) | 1251 | 86.9 |
| Complete and duplicated BUSCOs (D) | 39 | 2.7 |
| Fragmented BUSCOs (F) | 38 | 2.6 |
| Missing BUSCOs (M) | 112 | 7.8 |
| Total BUSCO groups searched | 1440 | - |
