# Supplementary material for: Tung Tree (Vernicia fordii) Genome Provides A Resource for Understanding Genome Evolution and Improved Oil Production
Source: Genomics Proteomics Bioinformatics. 2020 Mar 26;17(6):558–75. doi: 10.1016/j.gpb.2019.03.006 (PMC7212303; doi:10.1016/j.gpb.2019.03.006)
Supplement: Supplementary data 37 [file mmc37.docx]

**Table S12 Functional annotation of genes in the tung tree genome**

| **Type** | | **Number** | **Percentage (%)** |
| --- | --- | --- | --- |
| Annotation | Swissprot | 18,144 | 63.8 |
|  | Trembl | 22,627 | 79.6 |
|  | Kegg | 6835 | 24.0 |
|  | GO | 12,581 | 44.3 |
|  | NR | 23,047 | 81.1 |
|  | InterProscan | 21,309 | 75.0 |
| Total | Annotated | 23,143 | 81.4 |
|  | Unannotated | 5279 | 18.6 |
|  | Gene | 28,422 | - |
