# Supplementary material for: Tung Tree (Vernicia fordii) Genome Provides A Resource for Understanding Genome Evolution and Improved Oil Production
Source: Genomics Proteomics Bioinformatics. 2020 Mar 26;17(6):558–75. doi: 10.1016/j.gpb.2019.03.006 (PMC7212303; doi:10.1016/j.gpb.2019.03.006)
Supplement: Supplementary data 39 [file mmc39.docx]

**Table S14 Non-coding RNAs in the tung tree genome**

| **Type** | | **Copy number** | **Average length (bp)** | **Total length (bp)** | **Percentage of genome (%)** |
| --- | --- | --- | --- | --- | --- |
| rRNA | rRNA | 116 | 983 | 105,204 | 0.009404 |
|  | 18S | 8 | 2384 | 19,069 | 0.001705 |
|  | 28S | 13 | 5865 | 76,247 | 0.006816 |
|  | 5.8S | 9 | 155 | 1392 | 0.000124 |
|  | 5S | 86 | 115 | 9888 | 0.000884 |
| snRNA | snRNA | 1414 | 107 | 151,107 | 0.013507 |
|  | CD-box | 1289 | 104 | 134,149 | 0.011992 |
|  | HACA-box | 39 | 125 | 4858 | 0.000434 |
|  | Splicing | 86 | 141 | 12,100 | 0.001082 |
| miRNA | | 465 | 148 | 68,972 | 0.006165 |
| tRNA | | 740 | 74 | 55,083 | 0.004924 |
