# Supplementary material for: Tung Tree (Vernicia fordii) Genome Provides A Resource for Understanding Genome Evolution and Improved Oil Production
Source: Genomics Proteomics Bioinformatics. 2020 Mar 26;17(6):558–75. doi: 10.1016/j.gpb.2019.03.006 (PMC7212303; doi:10.1016/j.gpb.2019.03.006)
Supplement: Supplementary data 40 [file mmc40.docx]

**Table S15 Statistics of gene families of *V. forrdi* and 7 other species**

| **Species** | **Genes number** | **Genes number in**  **families** | **Unclustered genes number** | **Family number** | **Unique**  **families number** | **Average genes**  **number per family** |
| --- | --- | --- | --- | --- | --- | --- |
| *A. thaliana* | 27,173 | 22,858 | 4315 | 12,892 | 744 | 1.77 |
| *H. brasiliensis* | 34,661 | 30,787 | 3874 | 15,410 | 299 | 2 |
| *J. carcas* | 22,139 | 20,785 | 1354 | 14,969 | 166 | 1.39 |
| *M. esculenta* | 33,033 | 26,921 | 6112 | 15,641 | 184 | 1.72 |
| *P. trichocarpa* | 41,335 | 32,970 | 8365 | 15,376 | 803 | 2.14 |
| *R. communis* | 29,957 | 20,499 | 9458 | 15,294 | 565 | 1.34 |
| *V. fordii* | 28,422 | 22,991 | 5431 | 15,038 | 635 | 1.53 |
| *V. vinifera* | 26,346 | 19,025 | 7321 | 12,865 | 623 | 1.48 |

*Note*: Unclustered genes, species-specific genes that were not assigned to any families. Unique family, species-specific paralogous gene families.
