# Supplementary material for: Tung Tree (Vernicia fordii) Genome Provides A Resource for Understanding Genome Evolution and Improved Oil Production
Source: Genomics Proteomics Bioinformatics. 2020 Mar 26;17(6):558–75. doi: 10.1016/j.gpb.2019.03.006 (PMC7212303; doi:10.1016/j.gpb.2019.03.006)
Supplement: Supplementary data 49 [file mmc49.docx]

**Table S24 Summary of repeat sequences in the tung tree genome**

| **Type** | **Repeat size (bp)** | **Percentage of genome (%)** |
| --- | --- | --- |
| Trf | 49,820,132 | 4.70 |
| RepeatProteinMask | 222,266,652 | 20.97 |
| RepeatMasker (Repbase) | 194,834,599 | 18.38 |
| RepeatMasker (Mips-REdat) | 209,512,020 | 19.76 |
| Denovo (RepeatModeler) | 717,652,523 | 67.70 |
| Total | 777,464,634 | 73.34 |

*Note*: Total indicates non-redundant repeat size generated by the above five methods.
