# Supplementary material for: Tung Tree (Vernicia fordii) Genome Provides A Resource for Understanding Genome Evolution and Improved Oil Production
Source: Genomics Proteomics Bioinformatics. 2020 Mar 26;17(6):558–75. doi: 10.1016/j.gpb.2019.03.006 (PMC7212303; doi:10.1016/j.gpb.2019.03.006)
Supplement: Supplementary data 50 [file mmc50.docx]

**Table S25 Annotation of repeat sequences in the tung tree genome**

|  |  |  | **Count** | **Total repeat** | **Percentage of repeat (%)** | **Percentage of genome (%)** |
| --- | --- | --- | --- | --- | --- | --- |
| Total repeat fraction |  |  | 1,105,885 | 777,464,634 | 100 | 73.34 |
| Class i: retroelement |  |  | 403,333 | 550,023,721 | 70.74581877 | 51.88520894 |
|  | LTR retrotransposon |  | 382,737 | 538,155,445 | 69.21928297 | 50.76564271 |
|  |  | *Ty1*/*Copia* | 84,180 | 117,652,898 | 15.13289388 | 11.09851259 |
|  |  | *Ty3*/*Gypsy* | 284,597 | 415,630,052 | 53.45967313 | 39.20749463 |
|  |  | Other | 13,960 | 4,872,495 | 0.626715967 | 0.459635487 |
|  | Non-LTR retrotransposon |  | 20,585 | 11,867,162 | 1.526392518 | 1.119461137 |
|  |  | LINE | 19,094 | 11,683,118 | 1.502720187 | 1.102099774 |
|  |  | SINE | 1491 | 184,044 | 0.023672331 | 0.017361363 |
|  | Retroposon |  | 11 | 1114 | 0.000143286 | 0.000105087 |
| Class ii: DNA transposon |  |  | 140,493 | 59,048,669 | 7.595029589 | 5.570218904 |
|  |  | CMC | 22,828 | 6,803,128 | 0.875040189 | 0.641757263 |
|  |  | hAT | 5560 | 1,179,911 | 0.151763945 | 0.111304161 |
|  |  | PIF/Harbinger | 653 | 364,430 | 0.046874158 | 0.034377657 |
|  |  | Other | 111,452 | 50,701,200 | 6.521351298 | 4.782779824 |
| Simple_repeat | NA | NA | 222,091 | 80,716,851 | 10.3820608 | 7.614236476 |
| Unknown | NA | NA | 351,473 | 112,227,111 | 14.43501172 | 10.58668359 |
| Satellite | NA | NA | 690 | 112,112 | 0.014420206 | 0.010575825 |
