# Supplementary material for: Tung Tree (Vernicia fordii) Genome Provides A Resource for Understanding Genome Evolution and Improved Oil Production
Source: Genomics Proteomics Bioinformatics. 2020 Mar 26;17(6):558–75. doi: 10.1016/j.gpb.2019.03.006 (PMC7212303; doi:10.1016/j.gpb.2019.03.006)
Supplement: Supplementary data 52 [file mmc52.docx]

**Table S36 Cross-species comparison of No. of *NBS-coding* gene families**

| **Type** | ***V. fordii*** | ***S. indicum*** | ***R. communis*** | ***M. esculenta*** | ***J. curcas*** | ***H. brasiliensis*** | ***V. vinifera*** | ***A. thaliana*** | ***P. trichocarpa*** | ***Z. mays*** | ***O. sativa*** |
| --- | --- | --- | --- | --- | --- | --- | --- | --- | --- | --- | --- |
| TIR-NBS | 0 | 0 | 7 | 3 | 15 | 4 | 3 | 17 | 13 | 0 | 0 |
| TIR-NBS-LRR | 0 | 0 | 25 | 27 | 40 | 15 | 17 | 79 | 78 | 0 | 0 |
| CC-NBS | 23 | 25 | 17 | 15 | 6 | 35 | 18 | 8 | 19 | 11 | 53 |
| CC-NBS-LRR | 7 | 5 | 21 | 40 | 19 | 45 | 28 | 17 | 119 | 58 | 402 |
| NBS-LRR | 16 | 23 | 67 | 124 | 91 | 186 | 121 | 20 | 120 | 31 | 74 |
| NBS | 42 | 118 | 95 | 103 | 104 | 198 | 129 | 26 | 53 | 7 | 16 |
| Total | 88 | 171 | 232 | 312 | 275 | 483 | 316 | 167 | 402 | 107 | 543 |
