# Supplementary material for: Tung Tree (Vernicia fordii) Genome Provides A Resource for Understanding Genome Evolution and Improved Oil Production
Source: Genomics Proteomics Bioinformatics. 2020 Mar 26;17(6):558–75. doi: 10.1016/j.gpb.2019.03.006 (PMC7212303; doi:10.1016/j.gpb.2019.03.006)
Supplement: Supplementary data 54 [file mmc54.docx]

**Table S38 Cross-species comparison of number of oil-related gene families**

| **Gene** | ***V. fordii*** | ***J. curcas*** | ***R. communis*** | ***A. thaliana*** | ***S. indicum*** | ***G. max*** |
| --- | --- | --- | --- | --- | --- | --- |
| *ACCase* | 9 | 6 | 6 | 7 | 6 | 10 |
| *MAT* | 2 | 1 | 1 | 1 | 1 | 2 |
| *KAS* | 5 | 6 | 9 | 4 | 10 | 14 |
| *KAR* | 3 | 3 | 3 | 6 | 7 | 7 |
| *HAD* | 1 | 2 | 2 | 2 | 1 | 5 |
| *EAR* | 1 | 2 | 2 | 1 | 4 | 5 |
| *FATA* | 1 | 2 | 2 | 2 | 2 | 6 |
| *FATB* | 5 | 4 | 3 | 1 | 1 | 6 |
| *SAD* | 4 | 7 | 5 | 7 | 7 | 5 |
| *FAD2* | 1 | 1 | 1 | 1 | 1 | 5 |
| *FAD3/7* | 4 | 9 | 4 | 4 | 3 | 11 |
| *FADx/FAH12* | 2 | 2 | 1 | 0 | 1 | 2 |
| *LACS* | 8 | 2 | 2 | 2 | 9 | 22 |
| *DAG-CPT* | 1 | 1 | 1 | 2 | 2 | 2 |
| *PDCT* | 1 | 1 | 1 | 2 | 1 | 2 |
| *PP* | 6 | 8 | 6 | 6 | 7 | 19 |
| *LPAT* | 7 | 5 | 6 | 5 | 7 | 11 |
| *GPAT* | 9 | 8 | 9 | 7 | 15 | 27 |
| *PDAT* | 6 | 7 | 5 | 3 | 6 | 8 |
| *DGAT* | 4 | 4 | 3 | 3 | 3 | 11 |
| *OLE* | 5 | 6 | 5 | 17 | 7 | 14 |
| *PEPC* | 3 | 4 | 7 | 4 | 4 | 16 |
| Total | 88 | 91 | 84 | 87 | 105 | 210 |
