# Supplementary material for: Tung Tree (Vernicia fordii) Genome Provides A Resource for Understanding Genome Evolution and Improved Oil Production
Source: Genomics Proteomics Bioinformatics. 2020 Mar 26;17(6):558–75. doi: 10.1016/j.gpb.2019.03.006 (PMC7212303; doi:10.1016/j.gpb.2019.03.006)
Supplement: Supplementary data 55 [file mmc55.docx]

**Table S39 Colinear gene pairs in poplar (*P. trichocarpa*)**

| **BLOCK_NO** | **BLOCK_SCORE** | **E_VALUE** | **LOCUS_1** | **LOCUS_2** | **Ka** | **Ks** |
| --- | --- | --- | --- | --- | --- | --- |
| 37 | 486 | 8.00E−15 | Potri.001G013700 | Potri.006G191700 | 0.7001 | 3.2078 |
| 37 | 486 | 2.00E−128 | Potri.001G013800 | Potri.006G191400 | 0.3004 | 1.5427 |
| 37 | 486 | 2.00E−51 | Potri.001G013200 | Potri.006G191900 | 0.312 | 1.0684 |
| 37 | 486 | 2.00E−177 | Potri.001G012500 (FADx) | Potri.006G192000 (FAD2) | 0.1704 | 2.2608 |
| 37 | 486 | 1.00E−179 | Potri.001G010900 | Potri.006G193900 | 0.2506 | 1.5559 |
| 37 | 486 | 2.00E−161 | Potri.001G010500 | Potri.006G194100 | 0.2015 | 2.7668 |
| 37 | 486 | 4.00E−69 | Potri.001G009100 | Potri.006G195300 | 0.3406 | 1.7346 |
| 37 | 486 | 6.00E−45 | Potri.001G008500 | Potri.006G195700 | 0.6179 | 3.8136 |
| 37 | 486 | 0 | Potri.001G007900 | Potri.006G196000 | 0.1915 | 1.2513 |
| 37 | 486 | 0 | Potri.001G007800 | Potri.006G196100 | 0.0579 | 1.2053 |
| 37 | 486 | 1.00E−160 | Potri.001G007000 | Potri.006G196900 | 0.2857 | 1.6713 |
| 37 | 486 | 1.00E−52 | Potri.001G006400 | Potri.006G197000 | 0.3281 | 2.0095 |
| 104 | 286 | 2.00E−132 | Potri.001G018900 | Potri.016G052700 | 0.2472 | 1.018 |
| 104 | 286 | 6.00E−180 | Potri.001G018700 | Potri.016G051900 | 0.3734 | 1.3881 |
| 104 | 286 | 2.00E−79 | Potri.001G018600 | Potri.016G051700 | 0.1742 | 1.2215 |
| 104 | 286 | 0 | Potri.001G015700 | Potri.016G049100 | 0.0993 | 1.6092 |
| 104 | 286 | 9.00E−137 | Potri.001G013800 | Potri.016G047600 | 0.2824 | 1.5449 |
| 104 | 286 | 0 | Potri.001G013500 | Potri.016G046700 | 0.1247 | 1.3067 |
| 104 | 286 | 2.00E−119 | Potri.001G013200 | Potri.016G046600 | 0.2814 | 1.1618 |
| 104 | 286 | 0 | Potri.001G012500 (FADx) | Potri.016G046200 (FAD2) | 0.1753 | 3.1035 |
